# Thioester deprotection using a biomimetic NCL approach

**DOI:** 10.3389/fchem.2022.934376

**Published:** 2022-08-22

**Authors:** Valentina Villamil, Cecilia Saiz, Graciela Mahler

**Affiliations:** ^1^ Laboratorio de Química Farmacéutica (DQO), Facultad de Química, Universidad de la República, Montevideo, Uruguay; ^2^ Graduate Program in Chemistry, Facultad de Química, Universidad de la República (UdelaR), Montevideo, Uruguay

**Keywords:** thioester deprotection, transthioesterification, native chemical ligation, bisthiazolidine, thiol

## Abstract

The reversibility of the thiol-thioester linkage has been broadly employed in many fields of biochemistry (lipid synthesis) and chemistry (dynamic combinatorial chemistry and material science). When the transthioesterification is followed by a S-to-N acyl transfer to give an amide bond, it is called Native Chemical Ligation (NCL), a high-yield chemoselective process used for peptide synthesis. Recently, we described thioglycolic acid (TGA) as a useful reagent for thioester deprotection both in solution and anchored to a solid-support under mild conditions. Inspired by NCL, in this work, we extended this approach and explored the use of 2-aminothiols for the deprotection of thiols bearing an acyl group. The best results were obtained using cysteamine or L-cysteine in an aqueous buffer pH 8 at room temperature for 30 min. The described approach was useful for S-acetyl, S-butyryl, and S-benzoyl heterocycles deprotection with yields up to 84%. Employing this methodology, we prepared six new analogs **2** of mercaptomethyl bisthiazolidine **1**, a useful inhibitor of a wide-range of Metallo-β-Lactamases (MBLs). Compared with the previous methodologies (TGA polymer supported and TGA in solution), the biomimetic deprotection herein described presents better performance with higher yields, shorter reaction times, less time-consuming operations, easier setup, and lower costs.

## Introduction

The thiol-thioester exchange occurs smoothly in neutral aqueous media at room temperature and is a highly chemoselective process. Due to these ideal features, this exchange reaction is found in many natural processes, including energy metabolism, mitosis, and autophagy (Pietrocola et al., 2015). Moreover, the abundance of thioester-mediated processes in nature has led to speculation regarding the role of these derivatives in the origin of life (Chandru et al., 2016; Vallee et al., 2017).

Besides the importance of the thiol-thioester exchange in biochemical processes, the reversibility of this linkage has been broadly used in the field of dynamic combinatorial chemistry (Mondal and Hirsch, 2015) and, more recently, applied in material science (Worrell et al., 2018).

**FIGURE 1:**
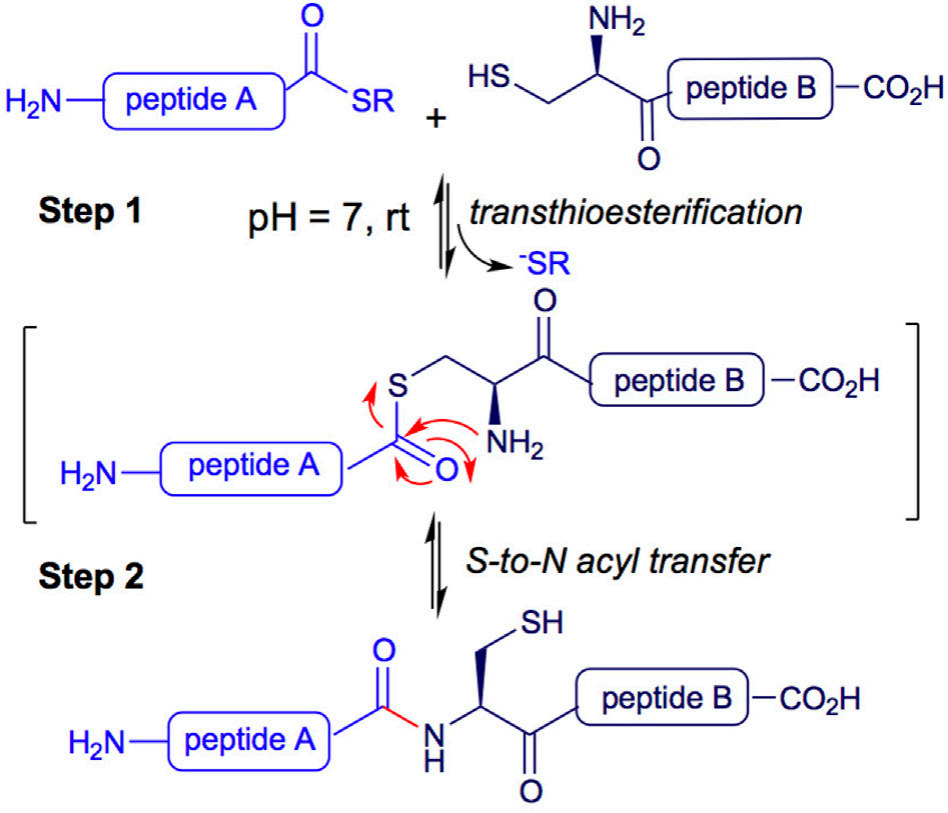
Native chemical ligation peptide synthesis.

In particular, Native Chemical Ligation (NCL) is a methodology used for peptide synthesis. It is a high-yielding chemoselective process, successfully applied to peptide and protein synthesis, chemical modification of proteins, protein-protein ligation, and the development of probes and molecular machines (Dawson et al., 1994; Gui et al., 2016; Burke et al., 2017). The NCL process begins with a reversible transthioesterification between two peptides. The mercapto group of an N-terminal cysteine residue of a peptide B attacks the C-terminal thioester of a second unprotected peptide A in an aqueous buffer at pH 7.0 (see Figure 1, Step 1). A second step consists of an S-to-N acyl transfer, leading to the formation of an amide bond between both peptides A and B (Figure 1, Step 2) (Serra et al., 2020).

**FIGURE 2:**
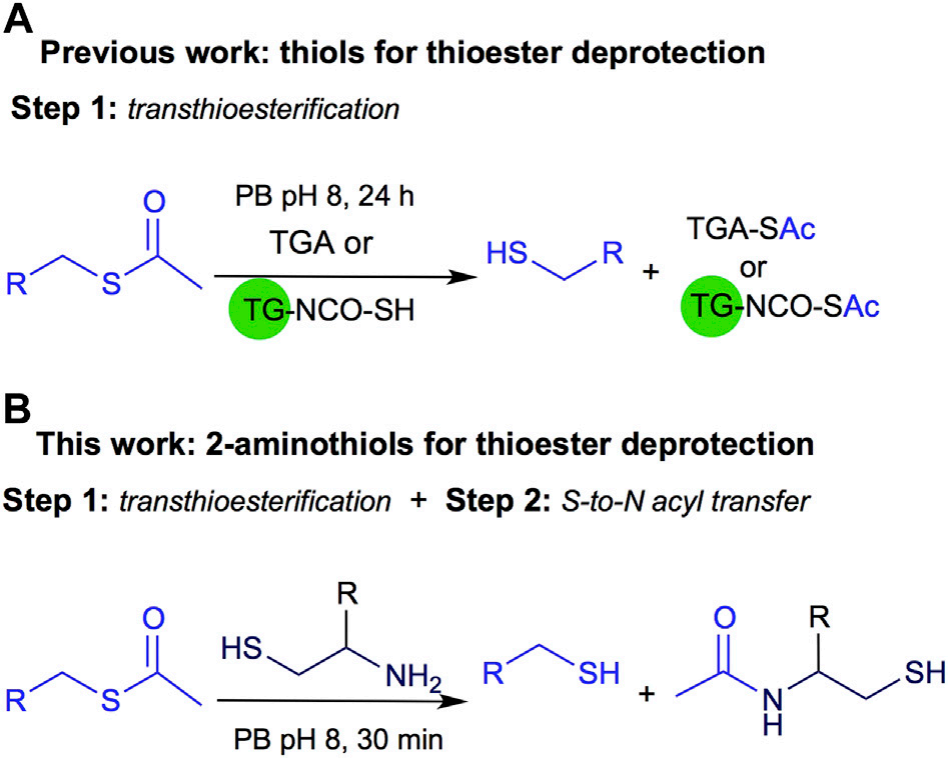
**(A)** Previous work on thioester deprotection using Method A polymer supported TGA (TG-NCO-SH) or Method B TGA in solution. **(B)** This work uses 2-aminothiols for thioacetyl deprotection.

Recently, we described another application of this reversible process applied to thiol deprotection in solution under mild conditions. This methodology is a valuable tool, especially when the substrate does not support harsh conditions like strong basic media. The deprotection is carried out using thioglycolic acid anchored to a solid support like TentaGel® resin (TG-NCO-SH, Method A) or in solution (Method B), Figure 2A (Villamil et al., 2021). Both homogeneous and heterogeneous approaches were conveniently carried out at room temperature, in aqueous buffer at pH 8, and the methods were useful for thioacetyl deprotection of several substrates, affording the free thiol after 24 h of reaction.

**FIGURE 3:**
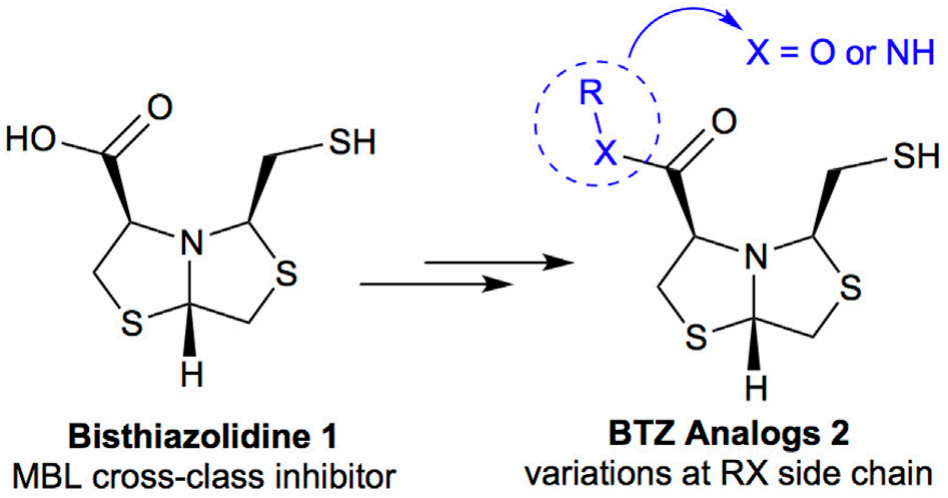
Bisthiazolidines **1** and BTZ analogs **2** with variations at RX.

Bisthiazolidines **1** (BTZ) are cross-class metallo-β-lactamases (MBLs) inhibitors active *in vitro* and against MBL-expressing bacterial pathogens, Figure 3. These compounds were designed as penicillin analogs and behaved as competitive inhibitors of MBLs from all subclasses, with *K_i_* values in the micromolar range. BTZ were effective against NDM-1, VIM-2, L1, and IMP-1, all MBLs of clinical importance (Mojica et al., 2015; González et al., 2016; Hinchliffe et al., 2016).

Previous studies showed that the mercaptomethyl group present in **1** is essential for MBL inhibition (Hinchliffe et al., 2016). With this in mind, we aimed to prepare new bisthiazolidine analogs **2** with variations on the carboxylic acid (R) in order to improve the inhibition activity. The synthetic route proposed includes thiol deprotection as the key step, see Figure 3.

Inspired by the NCL approach, in this work we propose to use 2-aminothiols for the deprotection of the thioacetyl derivatives as shown in Figure 2B. Although NCL is widely used in several fields of organic chemistry, to the best of our knowledge, it has not been explored for thioester deprotection in solution.

## Results and Discussion

**SCHEME 1:**
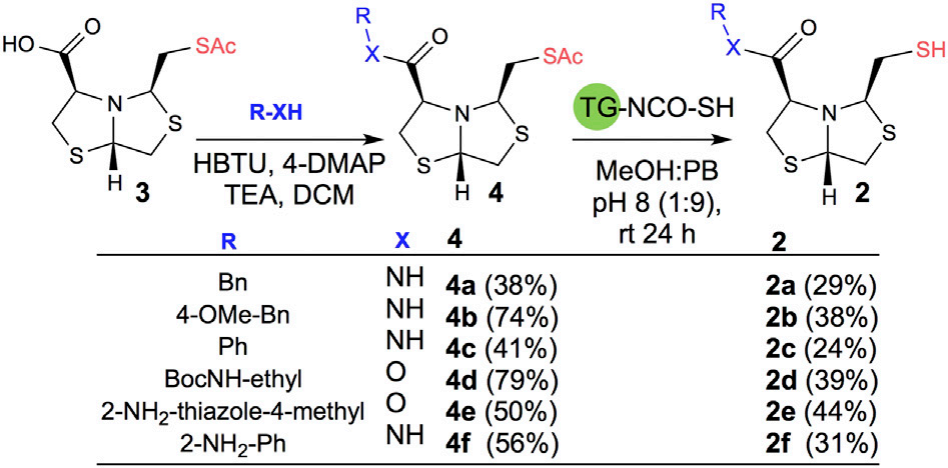
Synthesis of **2a-f** using acetyl deprotection: Method A: TG-NCO-SH (2 eq), MeOH:PB pH 8 (1:9), 24 h.

In order to prepare new BTZ analogs **2**, first we synthesized S-acetylated bisthiazolidine **3** as previously described (Villamil et al., 2021). Then, amides **4a-c**, **4f,** and esters **4d-e** were obtained starting from **3** and the corresponding amine or alcohol (R-XH), using HBTU as a coupling agent in DCM with yields ranging from 38 to 79%, see Scheme 1.

Based on our previous results, we performed the deprotection of thioacetylated compounds **4a-f** using TGA anchored to a TentaGel® resin (TG-NCO-SH), Method A. In this reaction, a transthioesterification process occurs between the thioester and the thiol. The mercaptomethyl derivatives **2a-f** were obtained after chromatographic column purification with yields ranging from 24 to 44%, as shown in Scheme 1.

Inspired by the Native Chemical Ligation (NCL) mechanism, where a transthioesterification reaction is followed by a 1-5 S,N-acyl transfer to form an amide and shift the equilibrium toward the product (Figure 1; Agouridas et al., 2019) and the report for the use of cysteamine (Cym) in acetonitrile under reflux for the conversion of simple and unfunctionalized thioesters into thiols (Endo et al., 1974), we envisioned that 2-aminothiols **5a-c** could be useful for the deprotection of acyl thioesters, as shown in Figure 2B.

In order to rapidly evaluate several sets of reaction conditions, we used reaction miniaturization, a concept developed by High Throughput Experimentation (HTE) to find the best reagents and conditions for a desired product. HTE is a tool that allows to perform a large number of experiments in parallel. The advantages of the reaction miniaturization studies is that they require a few milligrams of reagents (microscale) and shorter manipulation times compared to traditional experimentation (Wong and Cernak, 2018; Mennen et al., 2019).

Experimental and computational studies support the notion of the thiolate as the effective nucleophilic species in NCL (Wang et al., 2011; Diemer et al., 2022). In this sense, the aminothiols **5a-c** to be screened were selected basically based on the pKa value of the thiol, dismissing other possible ionic species (Maguire et al., 2020).

We reasoned that L-Cysteine ethyl ester (L-CysOEt, **5a**) bearing a thiol with a pKa = 6.5 could be first explored since it is close to neutral pH (Friedman et al., 1965). According to the Henderson-Hasselbach equation, the ratio of the L-CysOEt species SH/S^−^ is 1/2.5 in H_2_O at pH 7.30 (Henderson, 1908). A different aspect to consider is that thiolates are susceptible to oxidation (leading to disulfide species), a process that is accelerated when the pH of the solution is close to or above the thiol pKa.

Taking these considerations into account, the deprotection reaction of **3** was screened in aqueous buffers pH range from 5 to 8, aminothiol **5a** equivalents from 1 to 10 and the time from 0.5 to 6 h, see Supplementary Table S1. Aliquots were taken at different times and diluted for HPLC analysis. Overall, the best conditions for **3** deprotection were achieved using 2 eq L-CysOEt at pH 8, during 2 h to give **1** in 90% yield (see Supplementary Table S1). Since the thiol−thioester exchange is an equilibrium (Bongiardina et al., 2021), an excess of the deprotecting agent **5a** is required to shift the equilibrium toward the desired product. The reactions performed at pH 5 and 6 provided the less favorable yields, even when using more equivalents of 2-aminothiol or longer reaction times.

Based on these results, we extended the screening of 2-aminothiols including L-Cysteine (L-Cys, **5b**) and Cysteamine (Cym, **5c**) for the deprotection of thioacetyl bisthiazolidine **3**. These 2-aminothiols bear a thiol pKa value of 8.3 and 8.2 respectively (Pitman and Morris, 1979). Thus, to ensure a significant amount of thiolate, the reaction was performed at pH 7 (Table 1 entry 1–3) and pH 8 (Table 1, entry 4–7), and the reaction time was screened from 0.25 to 2 h using 2 equivalents of **5b/c**.

**TABLE 1:** Optimization of thioester deprotection using different 2-aminothiols **5a-c** for the deprotection of **3**.

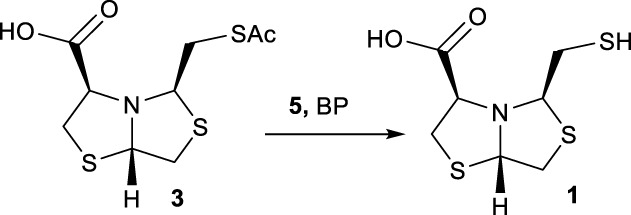					
			HPLC 1 yield (%)^[a]^		
	pH	Time (h)	L-CysOEt 5a	L-Cys 5b	Cym 5c
1	7	0.5	nd	64	69
2		1	nd	80	79
3		2	76	90	84
4	8	0.25	nd	62	62
5		0.5	68	83	89
6		1	nd	78	73
7		2	90	nd	nd

^[a]^Yields were determined using a validated HPLC technique in which BTZ **1** was used as external standard.

Both 2-aminothiols, L-Cys **5b** and Cym **5c,** gave similar yields compared to L-Cys-OEt **5a**. Overall, Cym **5c** was the best deprotecting agent, using 2 equivalents at pH 8 after only 30 min, affording **1** in 89% HPLC yield, Table 1, entry 5.

The HTE allowed us to find the best reaction conditions by running several experiments simultaneously rather than carrying out single ones, reducing the cost and time of the experiments. Overall, we performed 23 reactions in aqueous media, using less than 30 mg of compound **3** to find the optimal conditions for the deprotection reaction. This example shows the potential of the reaction miniaturization tool, mainly due to waste reduction and shorter time-consuming operations.

### Reaction scale-up for the deprotection of thioacetyl 3

Once the best reaction conditions were established, the reaction was scaled-up for deprotection of **3** using 2-aminothiols L-CysOEt **5a**, L-Cys **5b**, and Cym **5c** at pH 8 for 30 min. The isolated yields of **1** were 65%, 75%, and 78%, respectively, see Table 2, entries 1, 2, and 3. These results correlate well with the yields determined by HPLC at 68%, 83%, and 89%, respectively (Table 1), validating the results obtained by HTE. The three 2-aminothiols employed afford the free thiols in better yields than previously described TGA in solution (56% yield after 30 min), Table 2. The yield values for TGA in solution was obtained in a previous work (Villamil et al., 2021).

**TABLE 2:** Deprotection of thioester **3** under optimized conditions MeOH:BP (1:9), pH 8, rt, 30 min, aminothiol **5** (2 eq) or TGA (2 eq).

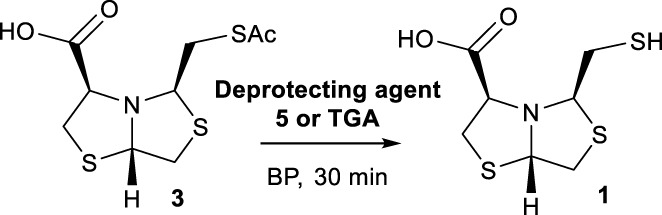		
Entry	Deprotecting agent (2 eq)	1 Yield (%)^[a]^
1	L-CysOEt **5a**	65
2	L-Cys **5b**	75
3	Cym **5c**	78
4	TGA^[b]^	56^[b]^

^[a]^Yields correspond to purified compounds by column chromatography on flash SiO_2_.

^[b]^ values were obtained from reference (Villamil et al., 2021).

Deprotection of thioacetyl bicycles **4a-f** using the optimized conditions (Method C: Cym **5c** pH 8, 30 min at rt) led to analogs **2a-f** in yields ranging from 50 to 78%, Table 3. The average yield was 65%, remarkably higher than those obtained using the TGA solid-supported approach (Method A, average yield of 34%). In addition, the reaction time decreased from 24 h to 30 min. This aspect is crucial since products **2a-f**, containing a free thiol, are easily oxidized in aqueous buffer. 2-aminothiols **5** allowed to set up reactions in shorter reaction times compared with TGA, affording better yields, probably by avoiding product oxidation.

**TABLE 3:** Thiol deprotection yield (%) using Method A, B and C of different substituted BTZ. Method A: TG-NCO-SH (2 eq) MeOH:BP pH 8 (1:9), 24 h; Method B: TGA (2 eq), MeOH:PB pH 8 (1:9), 0.5 or 24 h; Method C: Cym (2 eq), MeOH:PB pH 8 (1:9)0.5 h.

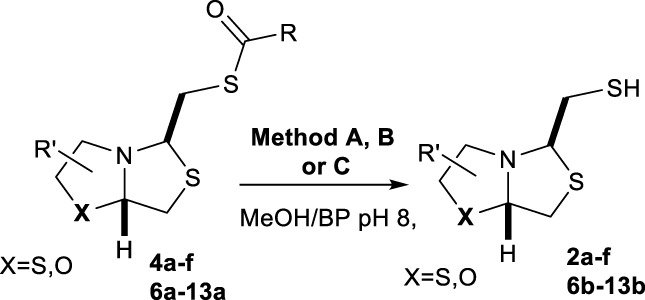						
Entry	Starting Material		Method, Yield %, (time h)			
			Product	A, (24 h)	B, (0.5 or 24 h)	C, (0.5 h)
1	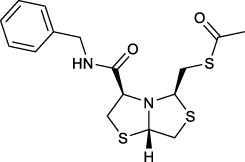	4a	2a	29	15 (0.5)	64
2	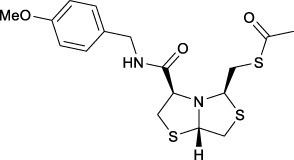	4b	2b	38	10 (0.5)	50
3	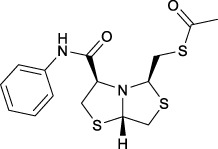	4c	2c	24	10 (0.5)	58
4	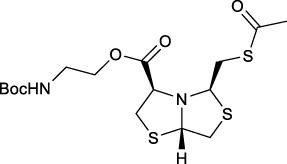	4d	2d	39	28 (0.5)	70
5	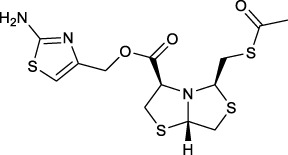	4e	2e	44	24 (0.5)	68
6	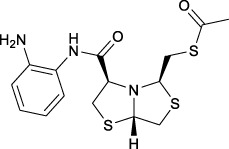	4f	2f	31	20 (0.5)	78
7	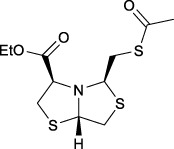	6a	6b	6,610	56 (24)*	59
8	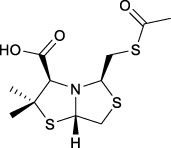	7a	7b	9,210	71 (24)*	84
9	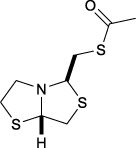	8a	8b	7,910	77 (24)*	80
10	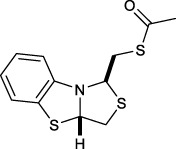	9a	9b	6,610	51 (24)*	59
11	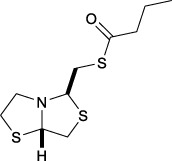	10a	8b	6,110	58 (24)*	51
12	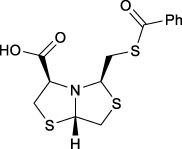	11a	1	7,610	75 (24)*	78
13	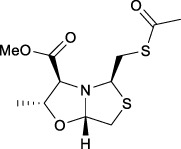	12a	12b	6,810	69 (24)*	80
14	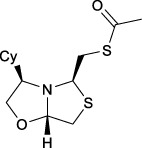	13a	13b	6,510	53 (24)*	61

Yields correspond to purified compounds by column chromatography on flash SiO_2_.

*: value extracted from (Villamil et al., 2021)..

In order to highlight the best performance of Cym regarding TGA in solution, we carried out the deprotection reaction of the new derivatives **4a-f** using method B (TGA in solution) after 30 min instead of 24 h, to compare the yields with method C (Cym in solution). The average yields were methods B and C, was 18% and 65%, respectively, significantly higher when using Cym, Table 3. The yield values for methods A and B (24 h) were obtained in a previous work (Villamil et al., 2021).

### Reaction scope

To extend the methodology scope, different S-acyl bisthiazolidines **6a-11a**, and S-acetyl oxazolidines **12a** and **13a** were assayed using Cym **5c** (Method C) and compared with our previous results using TGA solid-supported and TGA in solution (Method A and B, respectively) (Villamil et al., 2021), see Table 3.

Method C smoothly deprotected S-acetyl bisthiazolidines/oxazolidines **6a**-**9a**, **12a,** and **13a** with yields ranging from 59 to 84%, Table 3, entries 7–10, 13, and 14. In addition, different acyl side chains like propyl thioester **10a** and benzoyl thioester **11a** were deprotected in moderate to good yields, 51 and 78%, respectively, in Table 3, entries 11 and 12.

Altogether, the results obtained with our previous approaches (Methods A and B) led to slightly lower average yields compared to Method C (56, 64, and 67%, respectively) and shorter reaction times, 24 h vs 30 min. In addition, regarding method A, Cym is a common reagent in organic laboratories and is less expensive than Tentagel® resin.

Overall, Method C herein reported provides better yields in a shorter time with a lower-cost reagent and easier setup.

### Mechanistic insights

As described in NCL, the presence of a 2-aminothiol enables an intramolecular *S*-to-*N*-acyl migration, releasing the thiol and displacing the equilibrium toward the products (Wang et al., 2011). This fact suggests that when using Cym for thioester deprotection, an analogous process is occurring. In order to confirm the deprotection mechanism, NMR experiments were performed. Three sets of conditions were assayed in PB pH 8 using different deprotecting agents: 1) thioglycolic acid (TGA, 2 eq); 2) ethylenediamine (ETN, 2 eq); and 3) Cysteamine **5c** (2 eq).

**FIGURE 4:**
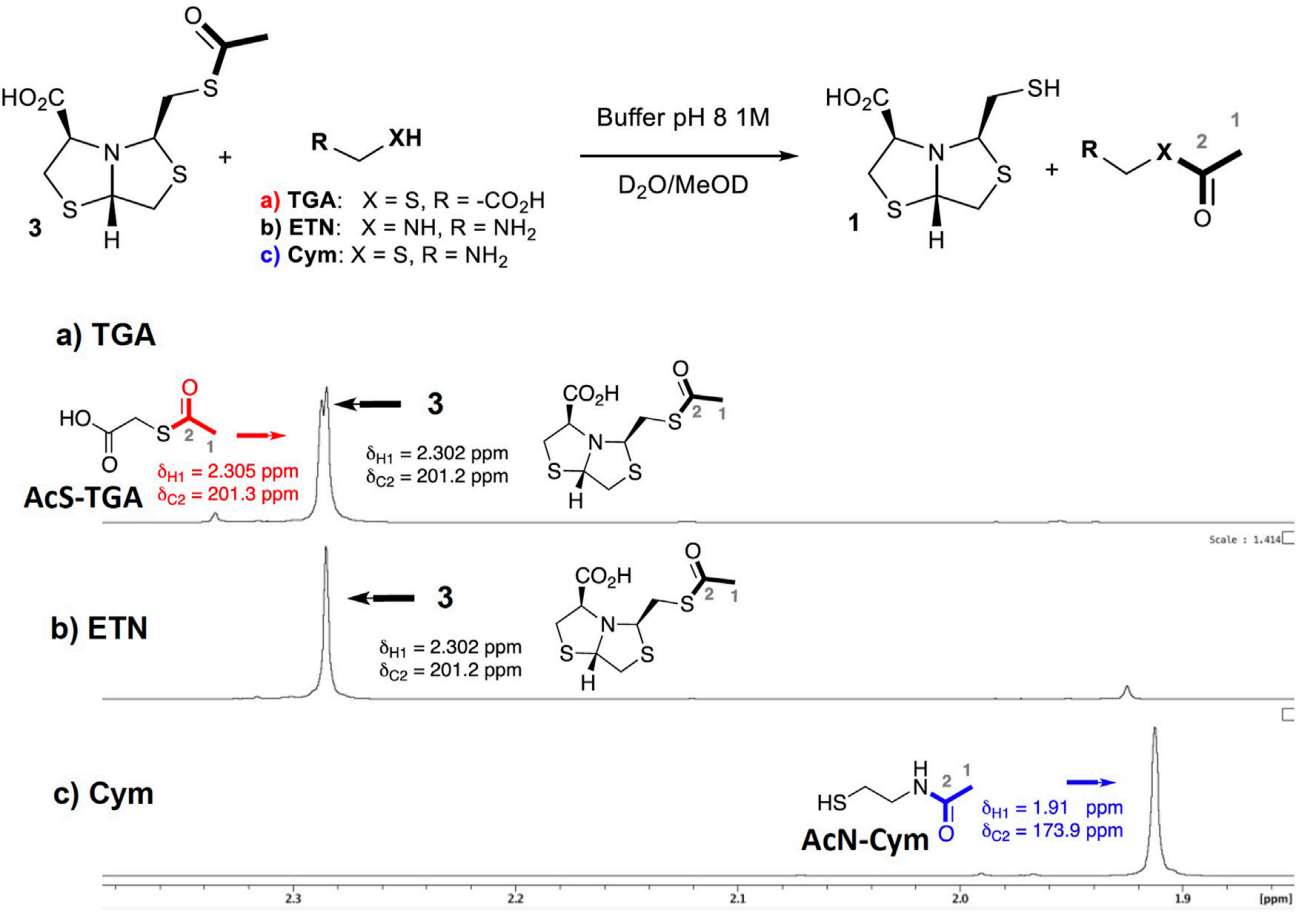
Comparison of ^1^H-NMR spectra for the deprotection reaction of **3** using different additives. Reaction of **3** (0.04 mmol) in D_2_O, PB [1 M] at pH 8, and MeOD-d4 (10%) at 27°C after 40 min: **(A)** TGA (0.08 mmol); **(B)** ethylenediamine (0.08 mmol); **(C)** Cym **5c** (0.08 mmol). The ^13^C chemical shift of C2 atoms was obtained from ^1^H,^13^C-HMBC correlations to the respective acetyl protons.

In the first experiment, when using TGA for thiol deprotection, after 40 min of reaction, 50% of the deprotection product was observed and thioacetylated TGA could be detected, Figure 4A.

Transthioesterification is the main deprotection mechanism in these conditions. A complete deprotection was observed after 9 h of incubation, Supplementary Figure S1.

On the other hand, when ethylenediamine (ETN) is used, the deprotection reaction is not observed by ^1^H NMR after 40 min, Figure 4B. This result indicates that only an amine group is not able to deprotect the thiol.

Finally, when Cym **5c** is used as a deprotecting agent, a complete thiol deprotection is observed after 40 min of incubation. Only N-acetylated cysteamine and free-thiol **1** were found in the ^1^H-NMR spectra, while S-acetylated cysteamine was not detected, see Figure 4C. The ^1^H-NMR spectra at t = 40 min comparison of experiments 1) and c) shows that the reaction rate is faster for c), observing a complete deprotection of **3**.

**FIGURE 5:**
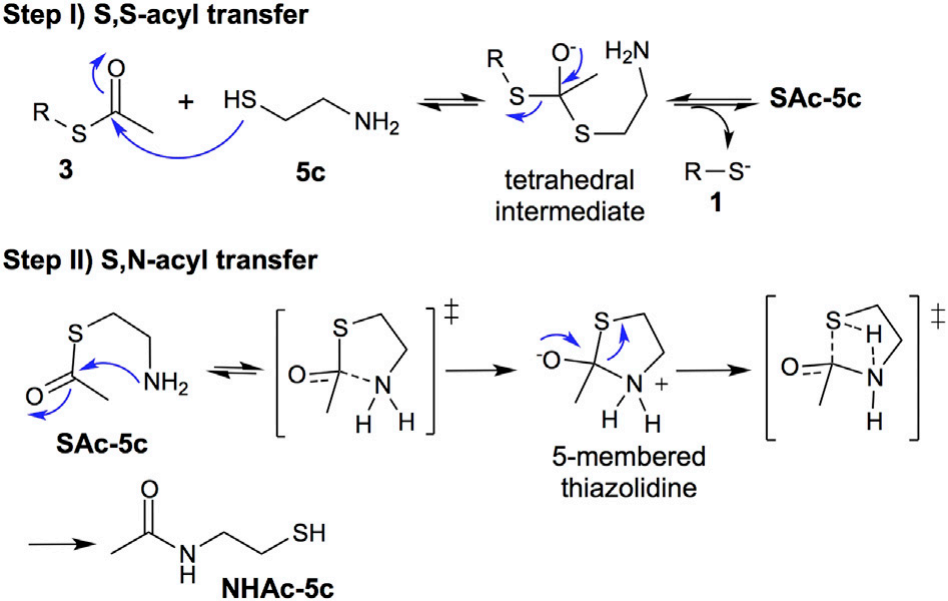
Mechanism proposal: step (I) reversible thiol−thioester exchange through a tetrahedral intermediate; step (II) irreversible thiazolidine intermediate to S,N-acyl transfer process.

Based on the obtained results and the numerous studies of the NCL mechanism (Barnett and Jencks, 1969; Wang et al., 2011), we propose, as a first step, a reversible S,S-acyl transfer involving an anionic concerted S_*N*_2 displacement mechanism with the formation of a tetrahedral intermediate, Figure 5, Step I (Barnett and Jencks, 1969). This is a common intermediate for the deprotection of thioesters using 2-aminothiols or thiols lacking the amino group.

The second step involves the *S,N*-acyl transfer, which could proceed by an *5-endo-trig* cyclization to form a thiazolidine intermediate, an irreversible step faster than the thiol−thioester exchange (Figure 5, step II).

In summary, the thiol-thioester exchange step is reversible, whereas the S,N-acyl transfer is irreversible, displacing the thiol-thioester exchange equilibrium toward the product **1** and N-acetylated cysteamine (NHAc-**5c**), increasing the deprotection reaction rate.

## Conclusion

In summary, we found that 2-aminothiols are suitable deprotecting agents for acyl thioester groups under aqueous mild conditions, mimicking a NCL approach. The reversibility of thiol−thioester exchange and the high efficacy of the intramolecular S,N-acyl migration process are driving forces to increase the reaction rate, affording shorter reaction times. In addition to this, the mild experimental conditions, near neutral pH, disfavor oxidation processes or bisthiazolidine-ring opening, increasing the deprotection reaction yields.

The new Cym deprotection methodology allowed us to prepare six new analogs of BTZ **1** with different substitutions at carboxylic acid (**2a-f**) to further be evaluated as MBL inhibitors.

The biomimetic deprotection herein described presents better performance than previous reported methodologies (TGA polymer supported and TGA in solution), with higher yields, less time-consuming operation, shorter reaction times, easier setup, and lower costs. We envision that these conditions could provide a simple and practical method for labile thioester hydrolysis.

## Experimental section

General Methods. All reactions were carried out in dry, freshly distilled solvents under anhydrous conditions unless otherwise stated. Reactions were monitored by analytical thin layer chromatography (TLC) on 0.25 mm silica gel coated plastic sheets (SIL G/UV 254). Flash chromatography on Silica gel 60 (40 µm average particle diameter) was used to purify the crude reaction mixtures. Yields are reported for chromatographic and spectroscopically (^1^H and ^13^C NMR) pure compounds unless otherwise stated. ^1^H and ^13^C NMR spectra were recorded on a Bruker Avance 400 instrument at 400 and 100 MHz, respectively. Chemical shifts (δ) are expressed in ppm downfield from TMS as an internal standard unless otherwise stated. Multiplicities are indicated as s (singlet), d (doublet), t (triplet), q (quartet), m (multiplet), b (broad). Assignments of ^1^H and ^13^C NMR peaks were made based on a combination of COSY, HSQC, and HMBC spectra. Electrospray ESI high-resolution mass spectra (HRMS) were recorded on a MicroTOF-Q spectrometer from Bruker Daltronics. Optical rotation was measured using a Jasco p-2000 polarimeter with a 2.0 ml cell, optical path length of 100 mm and sodium lamp (λ = 589 nm) at room temperature. The concentration c is given as g/100 ml.

HPLC Equipment and Method Validation. The liquid chromatography analysis was performed using Waters HPLC equipment, with binary pumps (Waters 1,525) and a photodiode array detector (Waters 2,996), with a loop injection of 20 μL (Rheodyne 1727). A reverse phase C18 separation column was used (Kinetex NUCLEOSIL® C18, 150 mm × 4 mm, 5 µm) with detection at λ = 205 nm for all compounds at 37°C. The eluent consisted of TFA 0.003 M (mobile phase A) and MeCN (mobile phase B) at a flow rate of 1.2 ml/min. The injection volume was 20 µl. Initial conditions 70/30 (mobile phase A/mobile phase B) changed in 2 min–55/45, maintained for 3.5 min, and changed in 0.1 min–5/95, maintained for 2.4 min. Data and chromatograms were collected and analyzed using the Empower System program by Waters Corporation, 2002. System linearity was verified in the concentration range: 0.01, 0.041, 0.102, 0.163, 0.203, and 0.244 mg/ml, prepared from a stock solution of BTZ **1**, 1 mM in MeOH. Linearity was established from the calibration curve using least squares linear regression analysis and a correlation coefficient (*R* ^2^) value of 0.9991 was found. A standard of 0.203 mg/ml was injected five times to evaluate the precision system, and a % RSD value of 1.0% was found.

### Synthetic procedures

Compounds **1**, (González et al., 2016; Hinchliffe et al., 2016), **3**, (Villam il et al., 2021), **6–13** (Saiz et al., 2014; Villamil et al., 2021) were prepared according to previous reports.

#### Method A: General method for deprotection using TG-NCO-SH

A solution of **4a** (0.0167 g, 0.0454 mmol) in MeOH (0.5 ml) and degassed BP pH 8 1M (1.0 ml) was added to the polymer-supported deprotecting agent (TG-NCO-SH, 0.222 g, 0.0908 mmol) prepared as previously described (Villamil et al., 2021) and stirred for 24 h at room temperature under a nitrogen atmosphere. Then, the polymer was filtered and washed with MeOH. The organic layer was dried over Na_2_SO_4_, filtered and evaporated. The mixture was poured into a NaCl saturated solution and extracted with EtOAc (3 × 30 ml). The combined organic layers were dried, filtered, and concentrated to dryness. The crude was purified by column chromatography on flash SiO_2_ (CH_2_Cl_2_/nHex 3:1) to yield **2a** (0.0043 g, 29%) as a colorless oil.

#### Method B: As previously described

(Villamil et al., 2021)

#### Method C: General method for deprotection using Cym

In a two-necked flask, **4a** (0.04 g, 0.11 mmol) was dissolved in MeOH (2 ml) and BP pH 8 1M (18 ml) and cysteamine hydrochloride **5c** (0.026 g, 0.22 mmol) was added. The reaction mixture was stirred for 30 min at room temperature, then poured into a NaCl saturated aqueous solution (30 ml) and extracted with EtOAc (3 × 30 ml). The combined organic layers were dried, filtered, and concentrated to dryness. The crude was purified by column chromatography on flash SiO_2_ (CH_2_Cl_2_/nHex 3:1) to yield **2a** (0.023 g, 64%) as a colorless oil.

### Optimization of the deprotection conditions of 3 using L-Cys-OEt 5a

Compound **3** was incubated into separate 2 ml Eppendorf® tubes at different pH and L-Cys-OEt **5a** concentrations, as described: **3** (100 µL of a solution of 42 mM in MeOH) was added to aqueous buffer 1M (pH 5, 6, 7 or 8, final volume 1 ml) and L-Cys-OEt **5a** (10, 20, 60 or 100 µl of a solution 420 mM in MeOH, corresponding to 1, 2, 6 or 10 equivalents) was added. The reaction solution was kept at 25°C and at suitable time intervals, aliquots of 100 µl were taken and acetonitrile (400 µl) was added. Then, the solution was vortexed, filtered, and chromatographed (Supplementary Table S1).

Optimization of the deprotection conditions of **3** using L-Cys **5b** and Cym **5c** was carried out using the same methodology as described for L-Cys-OEt **5a** (Table 1).

### Representative procedure for deprotection of 3 using L-Cys-OEt 5a in preparative scale

(Table 2) In a two-necked flask, compound **3** was dissolved (0.05 g, 0.18 mmol) in MeOH (1 ml) and added BP at pH 8 1M (19 ml) and L-Cys-OEt **5a** hydrochloride (0.067 g, 0.36 mmol). The reaction mixture was stirred for 30 min at room temperature. It was poured into HCl 5% and extracted with EtOAc (3 × 30 ml). The combined organic extracts were dried, filtered, and concentrated to dryness, and the crude was purified by column chromatography on flash SiO_2_ (nHex/EtOAc/AcOH 7:3:0.01) to yield **1** (0.028 g, 65%).

Deprotection of **3** using thiols **5b**, **5c,** and TGA was carried out using the same methods as described for **5a** (Table 2).

### Representative procedure for the synthesis of thioacetylated BTZ 4a-f

#### S-(((3R,5R,7aS)-3-(benzylcarbamoyl)tetrahydro-2H-thiazolo [4,3-b]thiazol-5-yl)methyl) ethanethioate (4a)

Into a two-necked flask under nitrogen atmosphere, **3** (0.1 , 0.36 mmol) was dissolved in dry CH_2_Cl_2_ (8 ml) and cooled in an ice bath. To the mixture was added HBTU (0.163 g, 0.43 mmol), DIPEA (0.139 g, 1.08 mmol), 4-DMAP (0.005 g, 0.036 mmol) and benzylamine (0.043 g, 0.4 mmol) and warmed to room temperature with stirring for 2 h. Then it was poured into a NaCl saturated aqueous solution (30 ml) and extracted with CH_2_Cl_2_ (3 × 30 ml). The combined organic layers were dried, filtered, and concentrated *in vacuo* to dryness. The crude product was purified by column chromatography on flash SiO_2_ (*n*Hex/EtOAc 3:1) to yield **4a** (0.051 g, 38%) as a white solid: MP = 61–65°C [α]_D_^20^ -54.9 (*c* = 0.91 in CH_2_Cl_2_); ^1^H NMR (400 MHz, CDCl_3_) δ 7.51 (br, 1H, NH), 7.37–7.28 (m, 5H, ArH), 4.94 (dd, *J* = 5.8, 4.1 Hz, 1H, CH), 4.53 (dd, *J* = 14.9, 6.3 Hz, 1H, CH-H), 4.44 (dd, *J* = 14.9, 5.7 Hz, 1H, CH-H), 4.33 (dd, *J* = 8.1, 5.2 Hz, 1H, CH), 4.09 (dd, *J* = 7.1, 2.9 Hz, 1H, CH), 3.55 (dd, *J* = 11.9, 5.8 Hz, 1H, CH-H), 3.52 (dd, *J* = 11.5, 2.5 Hz, 1H, CH-H), 3.27 (dd, *J* = 11.1, 7.1 Hz, 1H, CH-H), 3.19 (dd, *J* = 14.0, 5.2 Hz, 1H, CH-H), 3.13 (dd, *J* = 14.0, 8.1 Hz, 1H, CH-H), 3.09 (dd, *J* = 11.9, 4.0 Hz, 1H, CH-H), 2.14 (s, 3H, CH_3_); ^13^C{^1^H} NMR (100 MHz, CDCl_3_) δ 195.1, 170.3, 138.2, 128.9, 127.8, 127.7, 73.3, 72.8, 72.0, 43.6, 39.7, 37.8, 33.6, 30.6, HRMS (ESI/Q-TOF) m/z: [M + Na]^+^ Calcd for C_16_H_20_N_2_O_2_NaS_3_ 391.0585, found 391.0585.

#### S-(((3R,5R,7aS)-3-((4-methoxybenzyl)carbamoyl)tetrahydro-2H-thiazolo [4,3-b]thiazol-5-yl)methyl) ethanethioate (4b)

Prepared in an analogous route as described for **4a,** starting from **3** (0.12 g, 0.43 mmol) and 4-methoxybenzylamine (0.065 g, 0.47 mmol), purified by column chromatography on flash silica gel (*n*Hex/EtOAc 2:1) to yield **4b** (0.127 g, 74%) as a colorless oil [α]_D_^20^ −73.6 (*c* = 1.96 in CH_2_Cl_2_); ^1^H NMR (400 MHz, CDCl_3_) δ 7.43 (t, *J* = 5.9 Hz, 1H, ArH), 7.22 (dt, *J* = 4.9, 2.9 Hz, 2H, ArH), 6.88 (dt, *J* = 5.0, 3.0 Hz, 2H, ArH), 4.92 (dd, *J* = 5.8, 4.1 Hz, 1H, CH), 4.46 (dd, *J* = 14.6, 6.3 Hz, 1H, CH-H), 4.36 (dd, *J* = 14.6, 5.6 Hz, 1H, CH-H), 4.31 (dd, *J* = 7.9, 5.4 Hz, 1H, CH), 4.07 (dd, *J* = 7.1, 2.9 Hz, 1H, CH), 3.80 (s, 3H, CH_3_), 3.54 (dd, *J* = 11.9, 5.8 Hz, 1H, CH-H), 3.50 (dd, *J* = 11.1, 2.9 Hz, 1H, CH-H), 3.26 (dd, *J* = 11.1, 7.1 Hz, 1H, CH-H), 3.17 (dd, *J* = 13.6, 5.0 Hz, 1H, CH-H), 3.12 (dd, *J* = 13.7, 7.5 Hz, 1H, CH-H), 3.08 (dd, *J* = 11.9, 4.1 Hz, 1H CH-H), 2.17 (s, 3H, CH_3_); ^13^C{^1^H} NMR (100 MHz, CDCl_3_) δ 195.1, 170.1, 159.2, 130.3, 129.2, 114.2, 73.3, 72.7, 71.9, 55.4, 43.1, 39.6, 37.8, 33.6, 30.6. HRMS (ESI/Q-TOF) m/z: [M + Na]^+^ Calcd for C_17_H_22_N_2_O_3_NaS_3_ 421.0690, found 421.0687.

#### S-(((3R,5R,7aS)-3-(phenylcarbamoyl)tetrahydro-2H-thiazolo [4,3-b]thiazol-5-yl) methyl) ethanethioate (4c)

Prepared in an analogous route as described for **4a,** starting from **3** (0.15 g, 0.54 mmol) and aniline (0.055 g, 0.59 mmol), purified by column chromatography on flash SiO_2_ (*n*Hex/EtOAc 5:2) to yield **4c** (0.077 g, 41%) as a colorless oil [α]_D_^20^ −177.5 (*c* = 1.84 in CH_2_Cl_2_); ^1^H NMR (400 MHz, CDCl_3_) δ 9.03 (br, 1H, NH), 7.65 (d, *J* = 7.7 Hz, 2H, ArH), 7.35 (t, *J* = 7.9 Hz, 2H, ArH), 7.13 (t, *J* = 7.4 Hz, 1H, ArH), 5.03 (dd, *J* = 5.7, 4.6 Hz, 1H, CH), 4.42 (dd, *J* = 9.0, 4.2 Hz, 1H, CH), 4.12 (dd, *J* = 7.2, 3.1 Hz, 1H, CH), 3.63 (dd, *J* = 11.9, 5.9 Hz, 1H, CH-H), 3.54 (dd, *J* = 11.2, 3.0 Hz, 1H, CH-H), 3.33 (dd, *J* = 14.4, 3.9 Hz, 1H CH-H), 3.32 (dd, *J* = 11.7, 7.9 Hz, 1H, CH-H), 3.20 (dd, *J* = 14.1, 9.1 Hz, 1H, CH-H), 3.12 (dd, *J* = 11.9, 4.3 Hz, 1H, CH-H), 2.25 (s, 3H, CH_3_); ^13^C{^1^H} NMR (100 MHz, CDCl_3_) δ 195.3, 168.3, 137.6, 129.2, 124.7, 119.7, 73.2, 73.1, 71.9, 39.8, 37.8, 33.5, 30.8; HRMS (ESI/Q-TOF) m/z: [M + Na]^+^ Calcd for C_15_H_18_N_2_O_2_NaS_3_ 377.0428, found 377.0428.

#### S-((acetylthio)methyl)tetra hydro-2H-thiazolo [4,3-b]thiazole-3-carboxylate (4d)

Prepared in an analogous route as described for **4a** starting from **3** (0.1 g, 0.36 mmol) and N-boc-ethanolamine (0.069 g, 0.43 mmol), purified by column chromatography on flash SiO_2_ (*n*Hex/EtOAc 7:3) to yield **4d** (0.12 g, 79%) as a white powder: MP = 65–71°C [α]_D_^20^ -74.0 (*c* = 3.25 in CH_2_Cl_2_); ^1^H NMR (400 MHz, CDCl_3_) δ 5.15 (dd, *J* = 5.3, 3.4 Hz, 1H, CH), 5.05 (br, 1H, NH), 4.31 (t, *J* = 6.7 Hz, 1H, CH), 4.25–4.22 (m, 3H, CH, CH_2_), 3.58, (dd, *J* = 11.9, 5.4 Hz, 1H, CH-H), 3.45–3.43 (m, 2H, CH_2_), 3.34–3.26 (m, 3H, CH_2_, CH-H), 3.10 (dd, *J* = 11.6, 3.7 Hz, 1H, CH-H), 3.08 (dd, *J* = 13.9, 7.4 Hz, 1H, CH-H), 2.36 (s, 3H, CH_3_), 1.45 (s, 9H, (CH_3_)_3_); ^13^C{^1^H} NMR (100 MHz, CDCl_3_) δ 195.8, 170.5, 155.9, 79.7, 74.1, 71.6, 70.0, 64.9, 39.6, 38.4, 37.7, 34.3, 30.7, 28,5; HRMS (ESI/Q-TOF) m/z: [M + Na]^+^ Calcd for C_16_H_26_N_2_O_5_NaS_3_ 445.0902, found 445.0906.

#### (3R,5R,7aS)-(2-aminothiazol-5-yl)methyl 5-((acetylthio) methyl)tetrahydro-2H-thiazolo [4,3-b]thiazole-3-carboxylate (4e)

Prepared in an analogous route as described for **4a** starting from **3** (0.2 g, 0.7 mmol) and (2-aminothiazol-4-yl)methanol (0.1 g, 0.77 mmol), purified by column chromatography on flash silica gel (nHex/EtOAc 2:3) to yield **4e** (0.137 g, 50%) as a colorless oil [α]_D_^20^ −33.8 (*c* = 2.3 in CH_2_Cl_2_); ^1^H NMR (400 MHz, CDCl_3_) δ 6.51 (s, 1H, H (4)-thiazol), 5.64 (br, 2H, NH_2_), 5.18 (dd, *J* = 5.3, 3.3 Hz, 1H, CH), 5.06 (d, *J* = 12.6 Hz, 1H, CH-H), 5.02 (d, *J* = 12.6 Hz, 1H, CH-H), 4.29 (t, *J* = 6.8 Hz, 1H, CH), 4.28 (dd, *J* = 6.5, 5.0 Hz, 1H, CH), 3.58 (dd, *J* = 11.9, 5.4 Hz, 1H, CH-H), 3.34–3.24 (m, 3H, CH-H, CH_2_), 3.12–3.06 (m, 2H, CH_2_), 2.34 (s, 3H, CH_3_); ^13^C{^1^H} NMR (100 MHz, CDCl_3_) δ 195.7, 170.3, 168.7, 146.1, 107.6, 74.1, 71.5, 69.9, 62.7, 38.5, 37.7, 34.5, 30.7; HRMS (ESI/Q-TOF) m/z: [M + Na]^+^ Calcd for C_13_H_17_N_3_O_3_NaS_4_ 414.0050, found 414.0041.

#### *S-(((3R,5R,7aS)-3-((2-aminophenyl)carbamoyl)tetrahydro-2H-thiazolo[4,3-b] thiazol-5-yl)methyl) ethanethioate* (4f)

Prepared in an analogous route as described for **4a** starting from **3** (0.15 g, 0.54 mmol) and *o*-phenylenediamine (0.203 g, 1.88 mmol), purified by column chromatography on flash SiO_2_ (CH_2_Cl_2_/EtOAc 9:1) to yield **4f** (0.111 g, 56%) as a colorless oil: [α]_D_^20^ −100.7 (*c* = 4.5 in CH_2_Cl_2_); ^1^H NMR (400 MHz, CDCl_3_) δ 8.87 (br, 1H, NH), 7.23 (d, *J* = 7.8 Hz, 1H, ArH), 7.06 (t, *J* = 7.6 Hz, 1H, ArH), 6.80 (t, *J* = 7.5 Hz, 2H, ArH), 5.07 (t, *J* = 5.0 Hz, 1H, CH), 4.43 (dd, *J* = 8.1, 5.1 Hz, 1H, CH), 4.16 (dd, *J* = 7.0, 2.4 Hz, 1H, CH), 3.90 (br, 2H, NH_2_), 3.57 (dd, *J* = 11.9, 5.9 Hz, 1H, CH-H), 3.51 (dd, *J* = 11.3, 2.3 Hz, 1H, CH-H), 3.30–3.18 (m, 3H, CH_2_, CH-H), 3.09 (dd, *J* = 11.9, 4.1 Hz, 1H, CH-H), 2.26 (s, 3H, CH_3_); ^13^C{^1^H} NMR (100 MHz, CDCl_3_) δ 195.4, 169.1, 140.8, 127.5, 125.4, 123.4, 119.4, 117.9, 73.2, 72.9, 72.0, 39.7, 37.7, 33.6, 30.7; HRMS (ESI/Q-TOF) m/z: [M + Na]^+^ Calcd for C_15_H_19_N_3_O_2_NaS_3_ 392.0537, found 392.0540.

### Synthesis of analogs 2a-g

#### (3R,5R,7aS)-N-benzyl-5-(mercaptomethyl)tetrahydro-2H-thiazolo [4,3-b]thiazole-3-carboxamide (2a)

Yield: Method A (29%): Method C (64%)

^1^H NMR (400 MHz, CDCl_3_) δ 7.77 (br, 1H, NH), 7.36–7.28 (m, 5H, ArH), 4.88 (dd, *J* = 5.8, 5.0 Hz, 1H, CH), 4.49 (qd, *J* = 14.8, 6.0 Hz, 2H, CH_2_), 4.31 (dd, *J* = 8.1, 5.3 Hz, 1H, CH), 4.07 (dd, *J* = 7.3, 3.7 Hz, 1H, CH), 3.49 (dd, *J* = 11.0, 3.9 Hz, 1H, CH-H), 3.46 (dd, *J* = 11.6, 5.7 Hz, 1H, CH-H), 3.34 (dd, *J* = 11.2, 7.3 Hz, 1H, CH-H), 3.06 (dd, *J* = 11.8, 4.8 Hz, 1H, CH-H), 2.77 (ddd, *J* = 13.4, 8.1, 5.3 Hz, 1H, CH-H), 2.69 (dd, *J* = 13.9, 8.4 Hz, 1H, CH-H), 1.55 (t, *J* = 8.4 Hz, 1H, SH); ^13^C{^1^H} NMR (100 MHz, CDCl_3_) δ 170.3, 138.2, 128.9, 127.9, 127.7, 74.9, 72.9, 72.1, 43.6, 39.5, 33.8, 33.3; HRMS (ESI/Q-TOF) m/z [M + Na]^+^ Calcd for C_14_H_18_N_2_ONaS_3_ 349.0479, found 349.0478; [α]_D_^20^ -80.0 (*c* = 0.66 in CH_2_Cl_2_).

#### (3R,5R,7aS)-5-(mercaptomethyl)-N-(4-methoxybenzyl) tetrahydro-2H-thiazolo [4,3-b]thiazole-3-carboxamide (2b)

Prepared in an analogous route as described for **2a** starting from **4b** (0.0181 g, 0.0454 mmol), purified by column chromatography on flash SiO_2_ (nHex/EtOAc 3:1) to yield **2b** (Method A: 38%, Method C: 50%) as a colorless oil [α]_D_^20^ -108.5 (*c* = 1.0 in CH_2_Cl_2_); ^1^H NMR (400 MHz, CDCl_3_) δ 7.69 (br, 1H, NH), 7.23–7.19 (m, 2H, ArH), 6.88–6.85 (m, 2H, ArH), 4.86 (dd, *J* = 5.8, 4.9 Hz, 1H, CH), 4.47–4.36 (m, 2H, CH_2_), 4.29 (dd, *J* = 8.1, 5.3 Hz, 1H, CH), 4.05 (dd, *J* = 7.3, 3.8 Hz, 1H, CH), 3.80 (s, 3H, CH_3_), 3.46 (dd, *J* = 11.2, 3.8 Hz, 1H, CH-H), 3.44 (dd, *J* = 11.8, 6.0 Hz, 1H, CH-H), 3.32 (dd, *J* = 11.2, 7.3 Hz, 1H, CH-H), 3.05 (dd, *J* = 11.8, 4.8 Hz, 1H, CH-H), 2.76 (ddd, *J* = 13.6, 8.1, 5.3 Hz, 1H, CH-H), 2.68 (dt, *J* = 14.0, 8.4 Hz, 1H, CH-H), 1.56 (t, *J* = 8.4 Hz, 1H, SH); ^13^C{^1^H} NMR (100 MHz, CDCl3) δ 170.2, 159.2, 130.3, 129.2, 114.3, 74.9, 72.8, 72.1, 55.4, 43.0, 39.5, 33.8, 33.3; HRMS (ESI/Q-TOF) m/z: [M + Na]^+^ Calcd for C_15_H_20_N_2_O_2_NaS_3_ 379.0585, found 379.0591.

#### (3R,5R,7aS)-5-(mercaptomethyl)-N-phenyltetrahydro-2H-thiazolo [4,3-b]thiazole-3-carboxamide (2c)

Prepared in an analogous route as described for **2a** starting from **4c** (0.0161 g, 0.0454 mmol), purified by column chromatography on flash SiO_2_ (nHex/EtOAc 3:1) to yield **2c** (Method A: 24%, Method C: 58%) as a colorless oil [α]_D_^20^ −116.6 (*c* = 0.25 in CH_2_Cl_2_); ^1^H NMR (400 MHz, CDCl_3_) δ 9.44 (br, 1H, NH), 7.65 (dd, *J* = 8.5, 1.0 Hz, 2H, ArH), 7.36–7.31 (m, 2H, ArH), 7.15–7.11 (m, 1H, ArH), 4.99 (dd, *J* = 5.9, 5.2 Hz, 1H, CH), 4.43 (dd, *J* = 8.4, 4.7 Hz, 1H, CH), 4.11 (dd, *J* = 7.5, 3.5 Hz, 1H, CH), 3.57 (dd, *J* = 11.5, 3.7 Hz, 1H, CH-H), 3.54 (dd, *J* = 12.0, 5.9 Hz, 1H, CH-H), 3.38 (dd, *J* = 11.4, 7.5 Hz, 1H, CH-H), 3.12 (dd, *J* = 11.8, 5.0 Hz, 1H, CH-H), 2.96 (ddd, *J* = 14.0, 7.8, 4.7 Hz, 1H, CH-H), 2.84 (dd, *J* = 14.1, 8.5 Hz, 1H, CH-H), 1.82 (t, *J* = 8.2 Hz, 1H, SH); ^13^C{^1^H} NMR (100 MHz, CDCl_3_) δ 168.6, 137.7, 129.2, 124.7, 119.8, 74.6, 72.8, 72.4, 39.5, 33.6, 33.3. HRMS (ESI/Q-TOF) m/z: [M + Na]^+^ Calcd for C_13_H_16_N_2_ONaS_3_ 335.0322, found 335.0321.

#### (3R,5R,7aS)-2-((tert-butoxycarbonyl)amino)ethyl 5-(mercaptomethyl)tetrahydro-2H-thiazolo [4,3-b]thiazole-3-carboxylate (2d)

Prepared in an analogous route as described for **2a** starting from **4d** (0.0192 g, 0.0454 mmol), purified by column chromatography on flash SiO_2_ (nHex/EtOAc 8:2) to yield **2d** (Method A: 39%, Method C: 70%) as a colorless oil [α]_D_^20^ −35.9 (*c* = 2.13 in CH_2_Cl_2_); ^1^H NMR (400 MHz, CDCl_3_) δ 5.08 (dd, *J* = 5.3, 3.8 Hz, 1H, CH), 4.83 (br, 1H, NH), 4.31 (dd, *J* = 7.8, 5.9 Hz, 1H, CH), 4.25 (dd, *J* = 6.6, 4.8 Hz, 1H, CH), 4.24 (t, *J* = 5.3 Hz, 2H, CH_2_), 3.54 (dd, *J* = 12.0, 5.4 Hz, 1H, CH-H), 3.43 (t, *J* = 4.9 Hz, 2H, CH_2_), 3.34 (dd, *J* = 11.0, 4.9 Hz, 1H, CH-H), 3.27 (dd, *J* = 11.0, 6.6 Hz, 1H, CH-H), 3.09 (dd, *J* = 11.9, 3.8 Hz, 1H, CH-H), 2.88 (dt, *J* = 13.6, 7.5 Hz, 1H, CH-H), 2.64 (ddd, *J* = 13.6, 9.6, 5.9 Hz, 1H, CH-H), 1.97 (dd, *J* = 9.6, 7.2 Hz, 1H, SH), 1.45 (s, 9H, (CH_3_)_3_); ^13^C{^1^H} NMR (100 MHz, CDCl_3_) δ 170.6, 155.9, 79.9, 75.0, 73.5, 70.6, 64.8, 39.7, 39.3, 34.3, 33.9, 28.5; HRMS (ESI/Q-TOF) m/z: [M + Na]^+^ Calcd for C_14_H_24_N_2_O_4_NaS_3_ 403.0796, found 403.0796.

#### (3R,5R,7aS)-(2-aminothiazol-4-yl)methyl 5-(mercaptomethyl) tetrahydro-2H-thiazolo [4,3-b]thiazole-3-carboxylate (2e)

Prepared in an analogous route as described for **2a** starting from **4e** (0.0178 g, 0.0454 mmol), purified by column chromatography on flash SiO_2_ (nHex/EtOAc 2:3) to yield **2e** (Method A: 44%, Method C: 68%) as a colorless oil [α]_D_^20^ −33.4 (*c* = 1.63 in CH_2_Cl_2_); ^1^H NMR (400 MHz, CDCl_3_) δ 6.52 (s, 1H, H (4)-thiazole), 5.38 (br, 2H, NH_2_), 5.09 (dd, *J* = 5.2, 4.1 Hz, 1H, CH), 5.06 (d, *J* = 12.6 Hz, 1H, CH-H), 5.03 (d, *J* = 12.6 Hz, 1H, CH-H), 4.32 (dd, *J* = 7.2, 6.4 Hz, 1H, CH), 4.26 (dd, *J* = 6.6, 5.3 Hz, 1H, CH), 3.52 (dd, *J* = 11.9, 5.4 Hz, 1H, CH-H), 3.35 (dd, *J* = 11.0, 5.2 Hz, 1H, CH-H), 3.28 (dd, *J* = 11.0, 6.7 Hz, 1H, CH-H), 3.07 (dd, *J* = 11.9, 4.0 Hz, 1H, CH-H), 2.87 (dt, *J* = 13.7, 7.4 Hz, 1H, CH-H), 2.62 (ddd, *J* = 13.6, 9.4, 6.3 Hz, 1H, CH-H), 1.97 (dd, *J* = 9.4, 7.6 Hz, 1H, SH); ^13^C{^1^H} NMR (100 MHz, CDCl_3_) δ 170.4, 168.5, 146.2, 108.0, 75.2, 73.5, 70.4, 62.8, 39.2, 34.3, 33.8; HRMS (ESI/Q-TOF) m/z: [M + Na]^+^ Calcd for C_11_H_15_N_3_O_2_NaS_4_ 371.9945, found 371.9945.

#### (3R,5R,7aS)-N-(2-aminophenyl)-5-(mercaptomethyl) tetrahydro-2H-thiazolo [4,3-b]thiazole-3-carboxamide (2f)

Prepared in an analogous route as described for **2a** starting from **4f** (0.0168 g, 0.0454 mmol), purified by column chromatography on flash SiO_2_ (CH_2_Cl_2_/EtOAc 9:1) to yield **2f** (Method A: 31%, Method C: 78%) as a colorless oil [α]_D_^20^ -68.3 (*c* = 1.85 in CH_2_Cl_2_); ^1^H NMR (400 MHz, CDCl_3_) δ 9.19 (br, 1H, NH), 7.29 (dd, *J* = 8.2, 1.3 Hz, 1H, ArH), 7.08 (td, *J* = 7.8, 1.4 Hz, 1H, ArH), 6.84–6.80 (m, 2H, ArH), 5.06 (dd, *J* = 5.6, 4.7 Hz, 1H, CH), 4.45 (dd, *J* = 8.2, 5.0 Hz, 1H, CH), 4.24 (dd, *J* = 7.2, 2.7 Hz, 1H, CH), 3.60 (dd, *J* = 11.3, 2.7 Hz, 1H, CH-H), 3.55 (dd, *J* = 12.0, 5.9 Hz, 1H, CH-H), 3.35 (dd, *J* = 11.3, 7.2 Hz, 1H, CH-H), 3.13 (dd, *J* = 11.9, 4.5 Hz, 1H, CH-H), 2.97–2.80 (m, 2H, CH_2_), 1.84 (t, *J* = 8.2 Hz, 1H, SH); ^13^C{^1^H} NMR (100 MHz, CDCl_3_) δ 169.1, 140.4, 127.5, 125.1, 123.9, 119.8, 118.3, 75.0, 73.2, 73.1, 39.6, 33.7, 33.5, HRMS (ESI/Q-TOF) m/z: [M + Na]^+^ Calcd for C_13_H_17_N_3_ONaS_3_ 350.0431, found 350.0430.

Deprotection of **6–13** was carried out using the same approach as described for **4a**, Method C).

## Data Availability

The raw data supporting the conclusion of this article will be made available by the authors, without undue reservation.
