# Supplementary material for: Thioester deprotection using a biomimetic NCL approach
Source: Front Chem. 2022 Aug 22;10:934376. doi: 10.3389/fchem.2022.934376 (PMC9441695; doi:10.3389/fchem.2022.934376)
Supplement: Supplementary file 1 [file DataSheet1.PDF]

Supplementary Material

Table of contents **Page**

1) Experimental Section

General Methods……………………………………..………………………*S2*
HPLC Equipment and Method Validation…..…………………..………..………*S2*

2) Tables

Table S1: Deprotection reaction of S-acetyl-**3** using Cys-OEt...................................... *S3*

3) Figures

Figure S1………....……………………..…………..…………….………*S4*

4) ^{1}H NMR and $^{13}C\{^{1}H\}$ NMR Spectra

^{1}H NMR and $^{13}C\{^{1}H\}$ NMR Spectra of **4a-f**.. *S5*
^{1}H NMR and $^{13}C\{^{1}H\}$ NMR Spectra of **2a-f**... *S11*

5) Calibration curve and HPLC chromatograms

Calibration curve……………………………….…………………..……*S17*
HPLC chromatograms…………………………….…………………...……*S18*

Experimental Section

General Methods. All reactions were carried out in dry, freshly distilled solvents under anhydrous conditions unless otherwise stated. Reactions were monitored by analytical thin layer chromatography (TLC) on 0.25 mm silica gel coated plastic sheets (SIL G/UV 254). Flash chromatography on Silica gel 60 (40 µm average particle diameter) was used to purify the crude reaction mixtures. Yields are reported for chromatographic and spectroscopically (^{1}H and ^{13}C NMR) pure compounds unless otherwise stated. ^{1}H and ^{13}C NMR spectra were recorded on a Bruker Avance 400 instrument at 400 and 100 MHz respectively. Chemical shifts (δ) are expressed in ppm downfield from TMS as an internal standard unless otherwise stated. Multiplicities are indicated as s (singlet), d (doublet), t (triplet), q (quartet), m (multiplet), b (broad). Assignments of ^{1}H and ^{13}C NMR peaks were made based on a combination of COSY, HSQC, and HMBC spectra. Electrospray ESI high-resolution mass spectra (HRMS) were recorded on a MicroTOF-Q spectrometer from Bruker Daltronics. Optical rotation was measured using a Jasco p-2000 polarimeter with a 2.0 mL cell, optical path length of 100 mm and sodium lamp (λ=589 nm) at room temperature. The concentration c is given as g/100mL.

HPLC Equipment and Method Validation. The liquid chromatography analysis was performed using Waters HPLC equipment, with binary pumps (Waters 1525) and photodiode array detector (Waters 2996), with a loop injection of 20 µL (Rheodyne 1727). A reverse phase C18 separation column was used (Kinetex NUCLEOSIL® C18, 150 mm × 4 mm, 5 µm) with detection at λ = 205 nm for all compounds at 37°C. The eluent consisted of TFA 0.003 M (mobile phase A) and MeCN (mobile phase B) at a flow rate of 1.2 mL/min. The injection volume was 20 µL. Initial conditions 70/30 (mobile phase A/mobile phase B) changed in 2 min to 55/45, maintained for 3.5 minutes and changed in 0.1 min to 5/95, maintained for 2.4 minutes. Data and chromatograms were collected and analyzed using the Empower System program Waters Corporation, 2002. System linearity was verified in the concentration range: 0.01, 0.041, 0.102, 0.163, 0.203 and 0.244 mg/mL prepared from a stock solution of BTZ **1**, 1 mM in MeOH. Linearity was established from calibration curve using least squares linear regression analysis and a correlation coefficient (R^2) value of 0.9991 was found. A standard 0.203 mg/mL was injected five times to evaluate precision system, and a % RSD value of 1.0 % was found.

Eq. \ Time \ pH	pH 5		pH 6		pH 7		pH 8	
	Yield (%)							
	2h	6h	2h	6h	2h	6h	0.5 h	2h
1	4	14	16	26	53	36	39	71
2	9	26	20	30	76	60	68	90
6	22	53	23	40	76	58	ND	ND
10	38	71	25	43	77	62	ND	ND
0	0	0	0	0	0	0	0	0

Table S1. Yields as a function of pH, time and equivalents for deprotection reaction of S-acetyl-**3** using buffer and Cys-OEt. Color code: red 0-19% yield, orange 20-39%, yellow 40-59%, light green 60-80%, green 80-100. Yields were determined by HPLC. ND = Not determined.

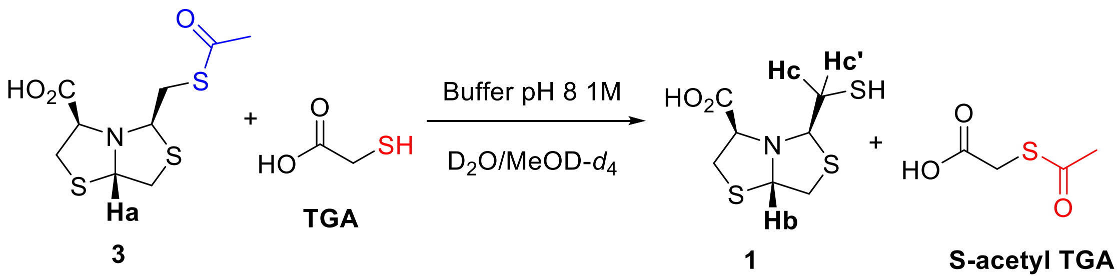

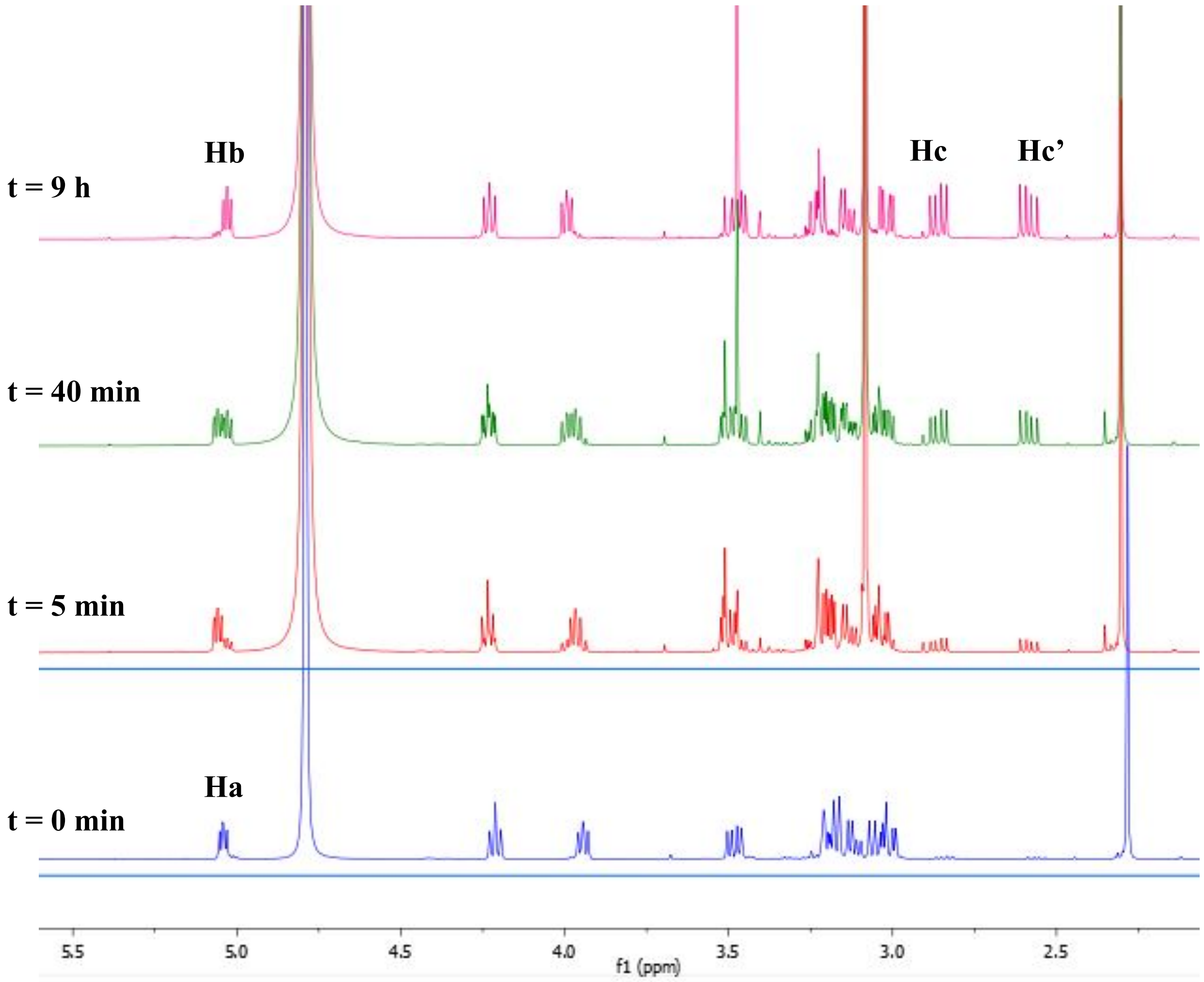

Figure S1: Time-course ^{1}H-NMR spectra of the reaction of **3** (0.04 mmol) in D_2O, PB [1 M] at pH 8, and MeOD-d_4 (10%) at 27 °C using TGA (0.08 mmol) as catalyst.

^{1}H NMR (CDCl$_3$ with 0.05% TMS, 400 MHz)

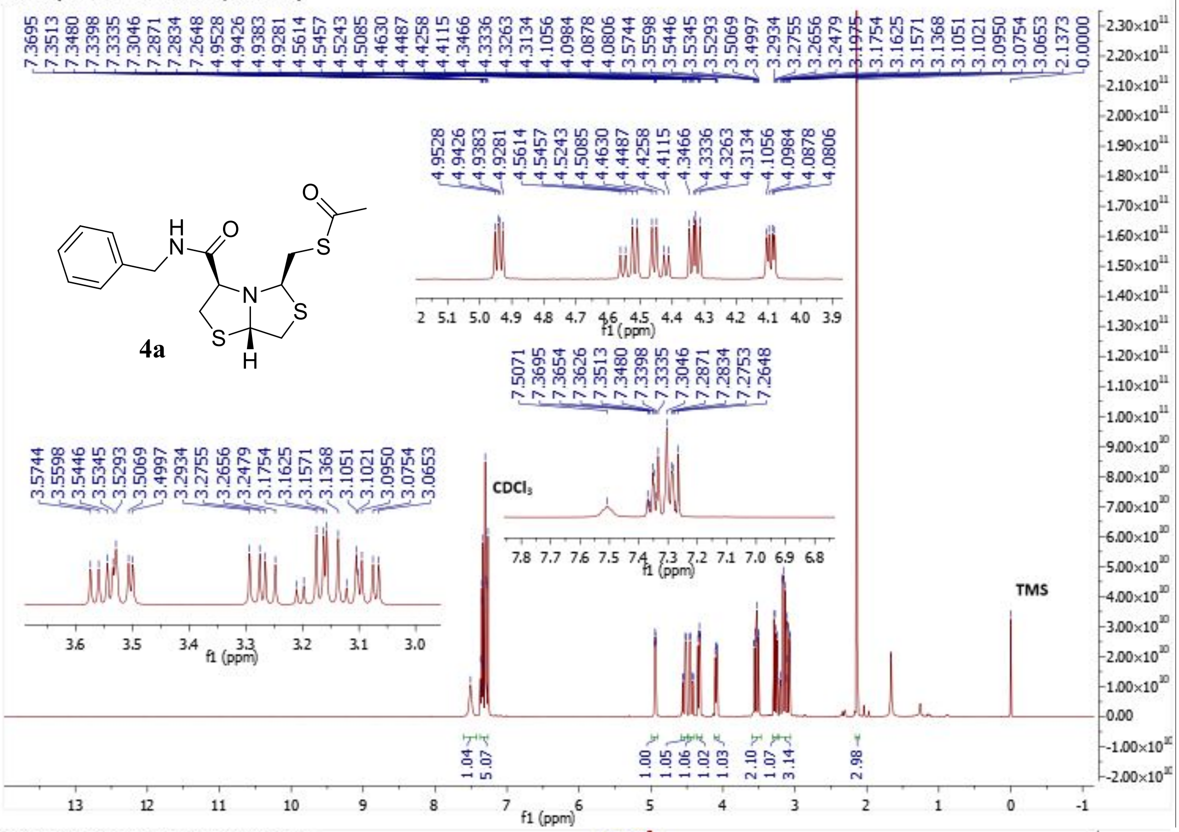

^{13}C NMR (CDCl$_3$ with 0.05% TMS, 100 MHz)

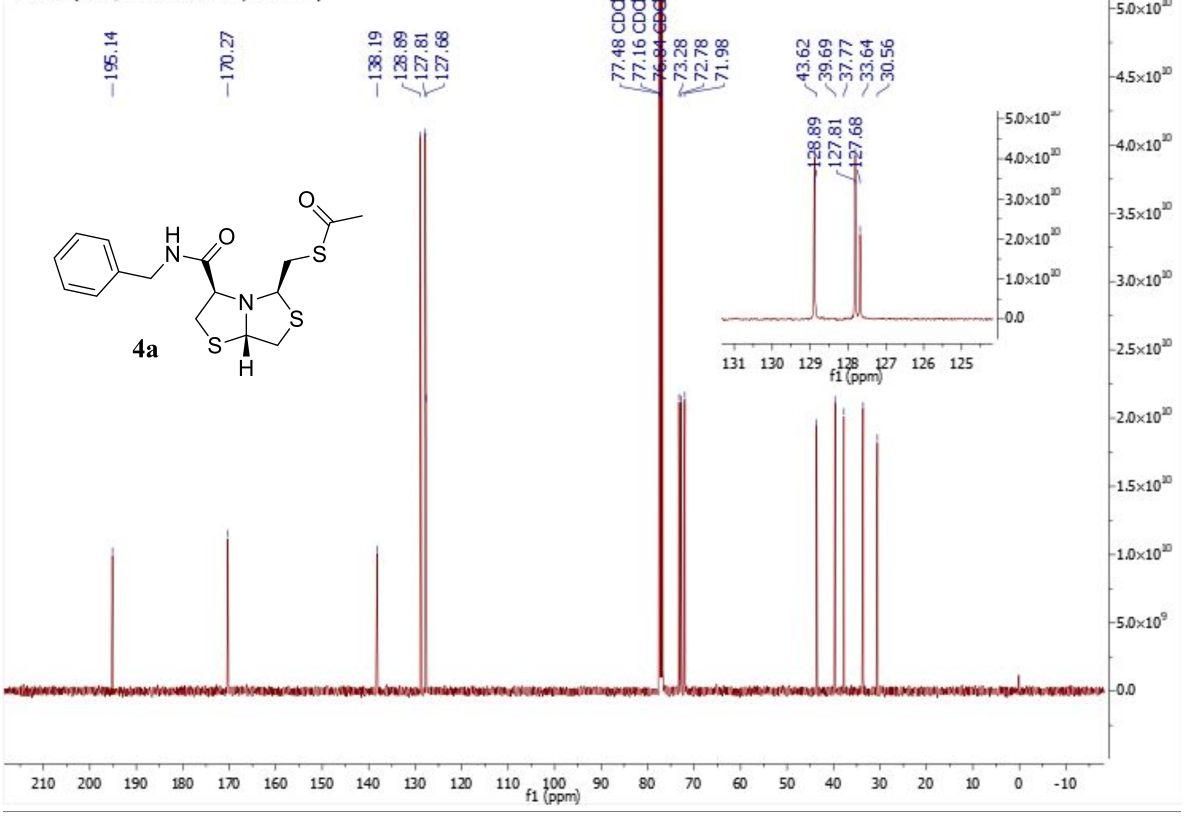

^{1}H NMR ($CDCl_3$ with 0.05% TMS, 400 MHz)

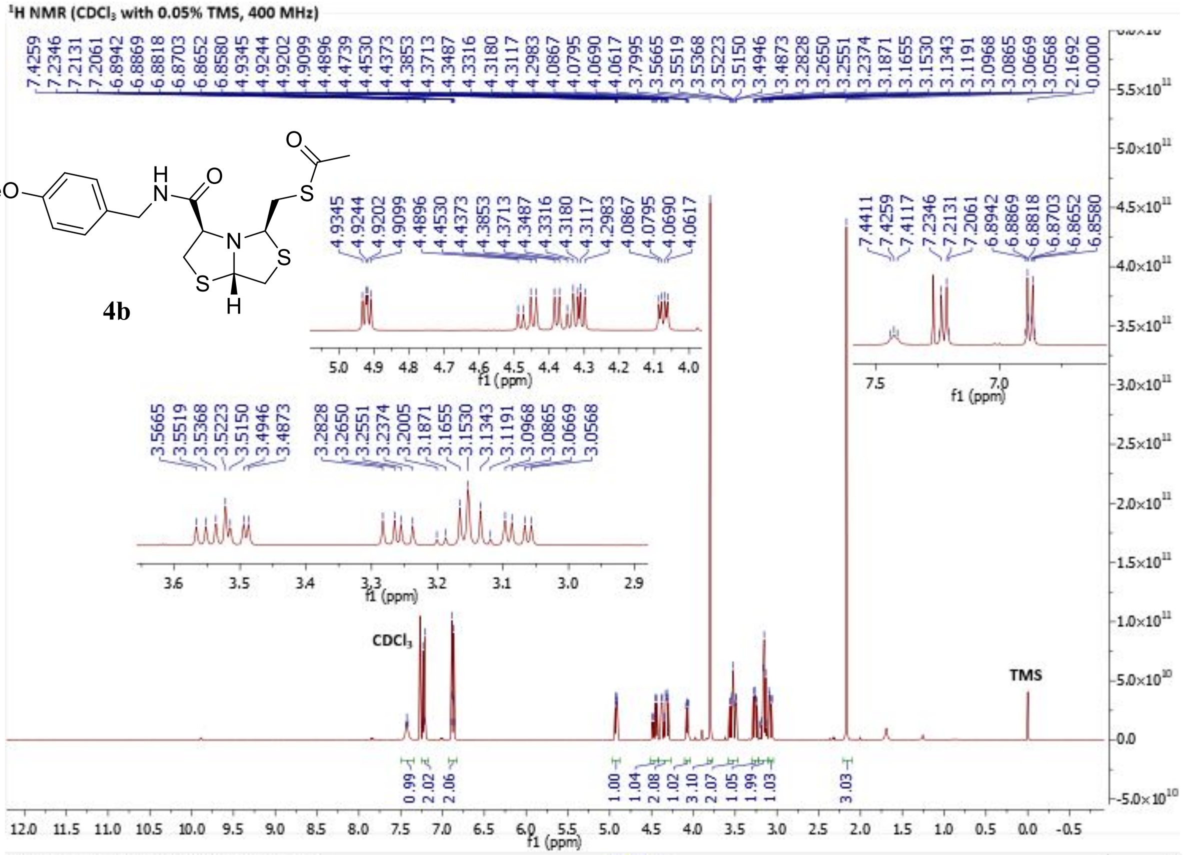

^{13}C NMR ($CDCl_3$ with 0.05% TMS, 100 MHz)

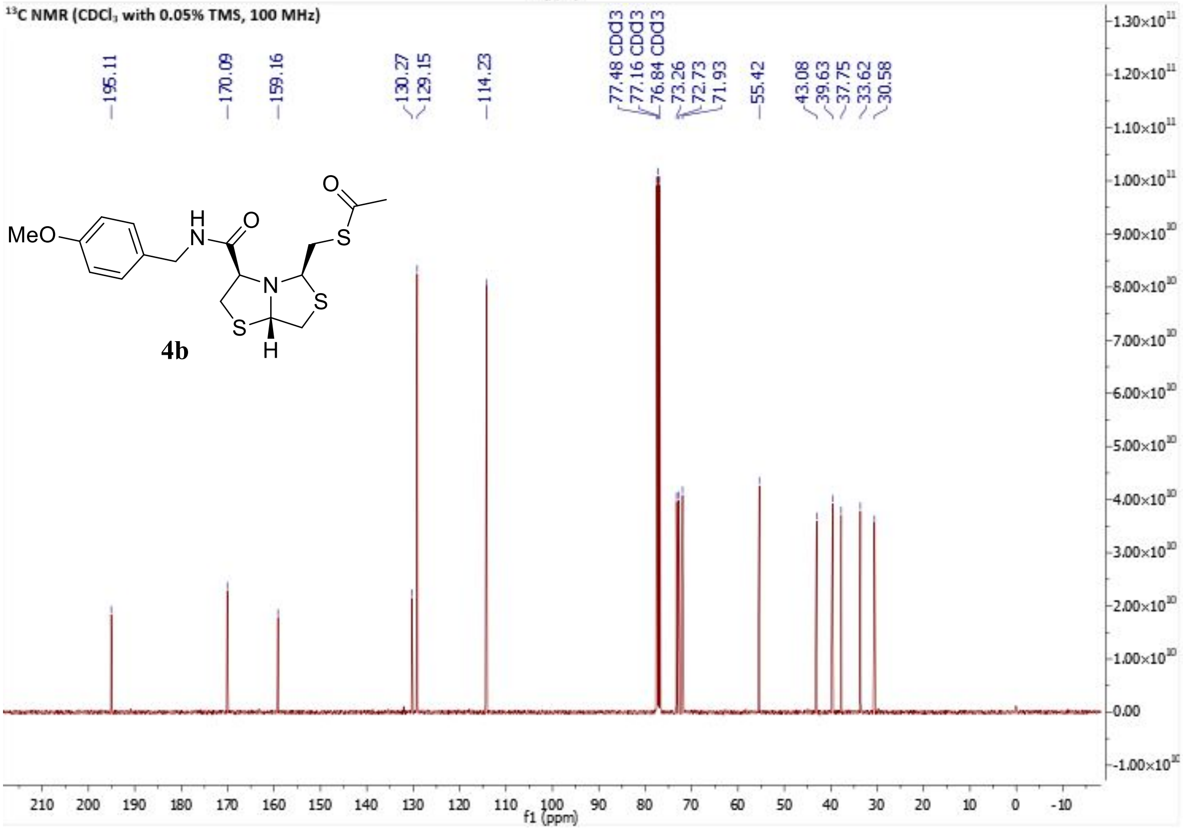

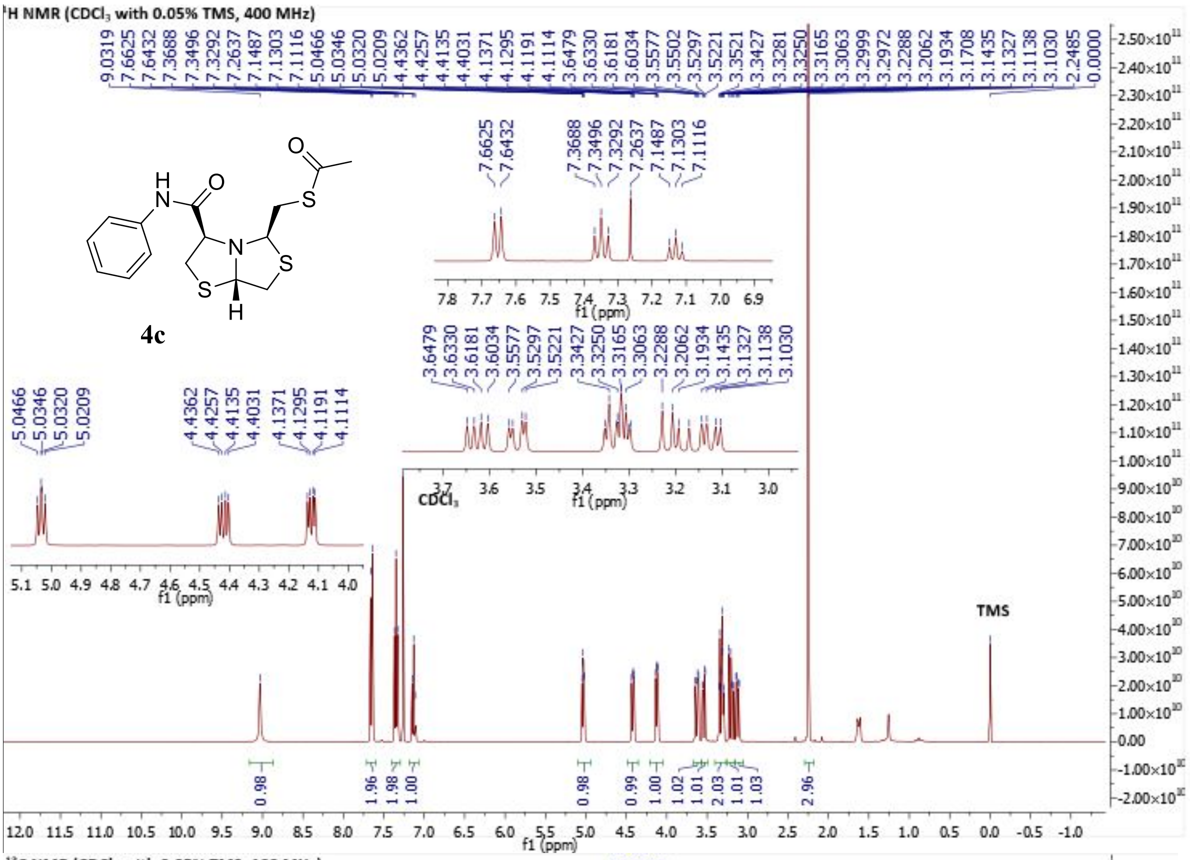
¹H NMR (CDCl₃ with 0.05% TMS, 400 MHz)
4c
CDCl₃
TMS
f1 (ppm)

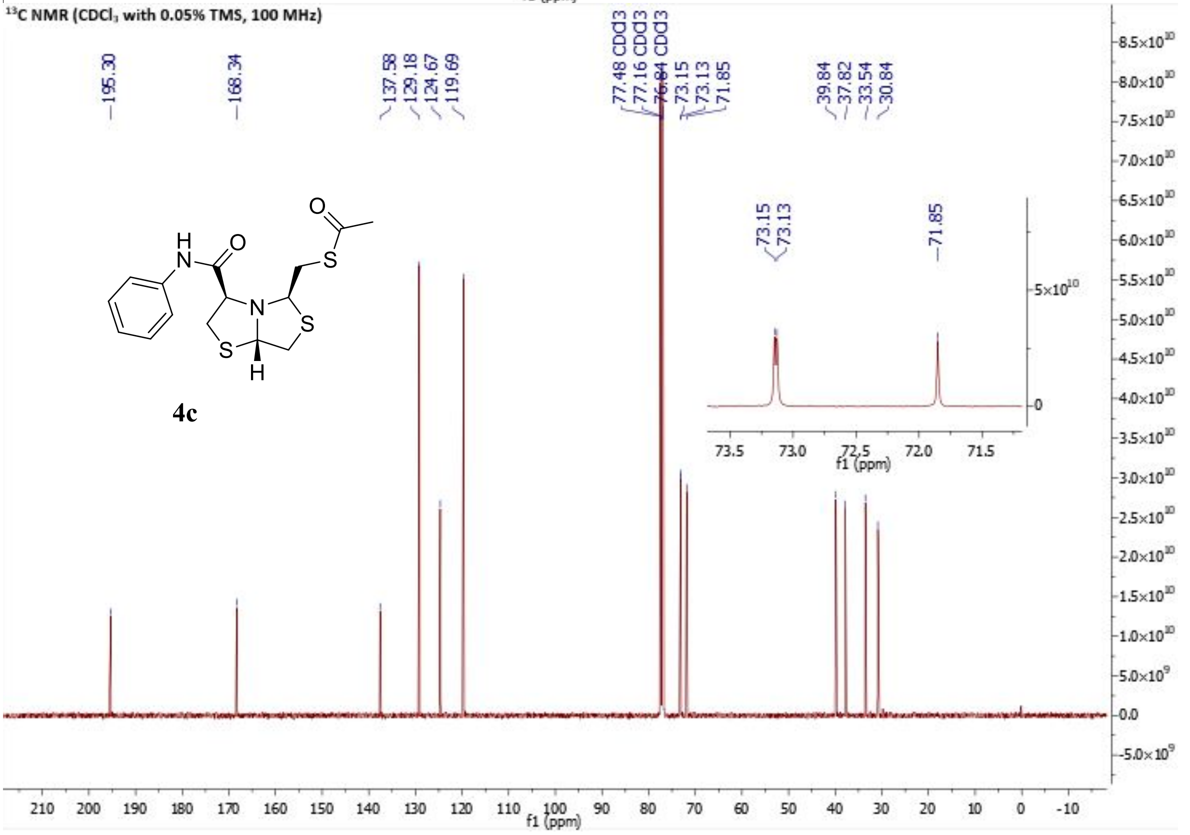
¹³C NMR (CDCl₃ with 0.05% TMS, 100 MHz)
4c
f1 (ppm)

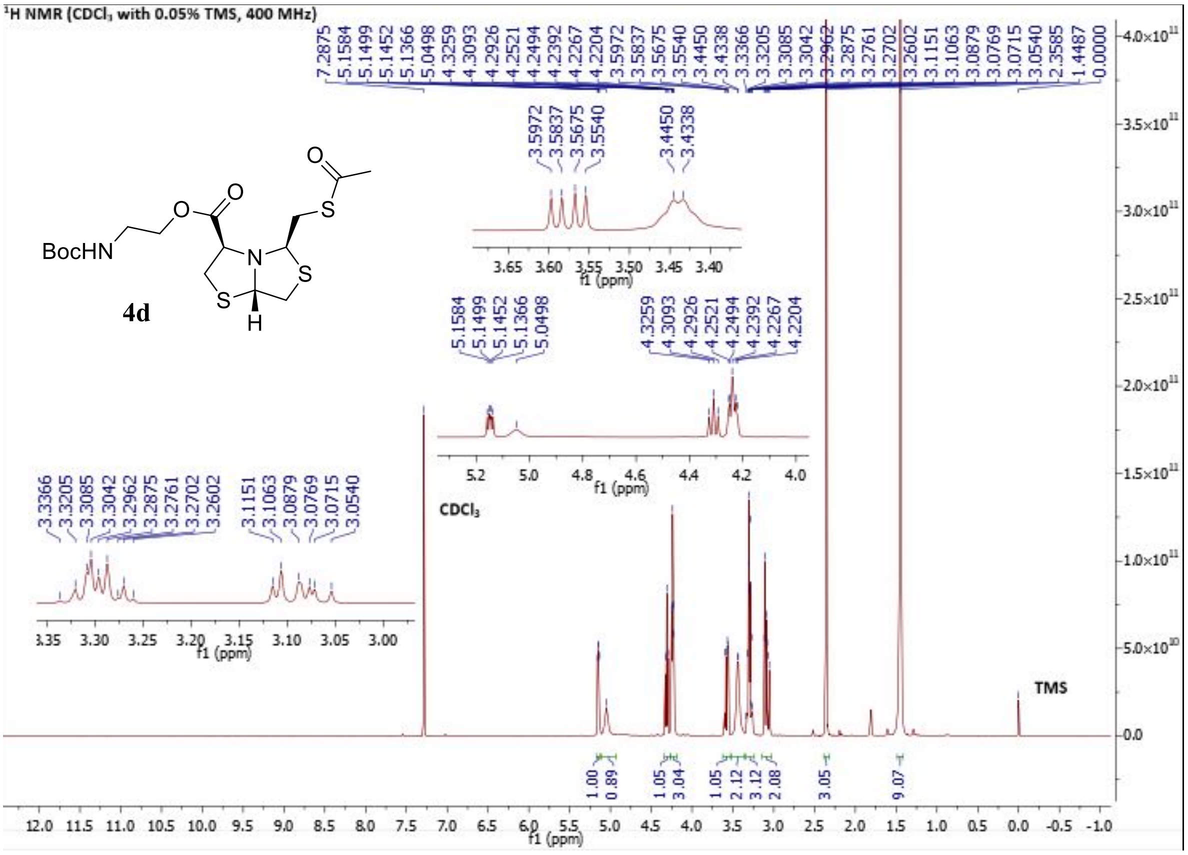

¹H NMR (CDCl₃ with 0.05% TMS, 400 MHz)
7.2875 5.1584 5.1499 5.1452 5.1366 5.0498 4.3259 4.3093 4.2926 4.2521 4.2494 4.2392 4.2267 4.2204 3.5972 3.5837 3.5675 3.5540 3.4450 3.4338 3.3366 3.3205 3.3085 3.3042 3.2962 3.2875 3.2761 3.2702 3.2602 3.1151 3.1063 3.0879 3.0769 3.0715 3.0540 2.3585 1.4487 0.0000
BocHN
4d
CDCl₃
TMS
1.00 0.89 1.05 3.04 1.05 2.12 3.12 2.08 3.05 9.07
f1 (ppm)

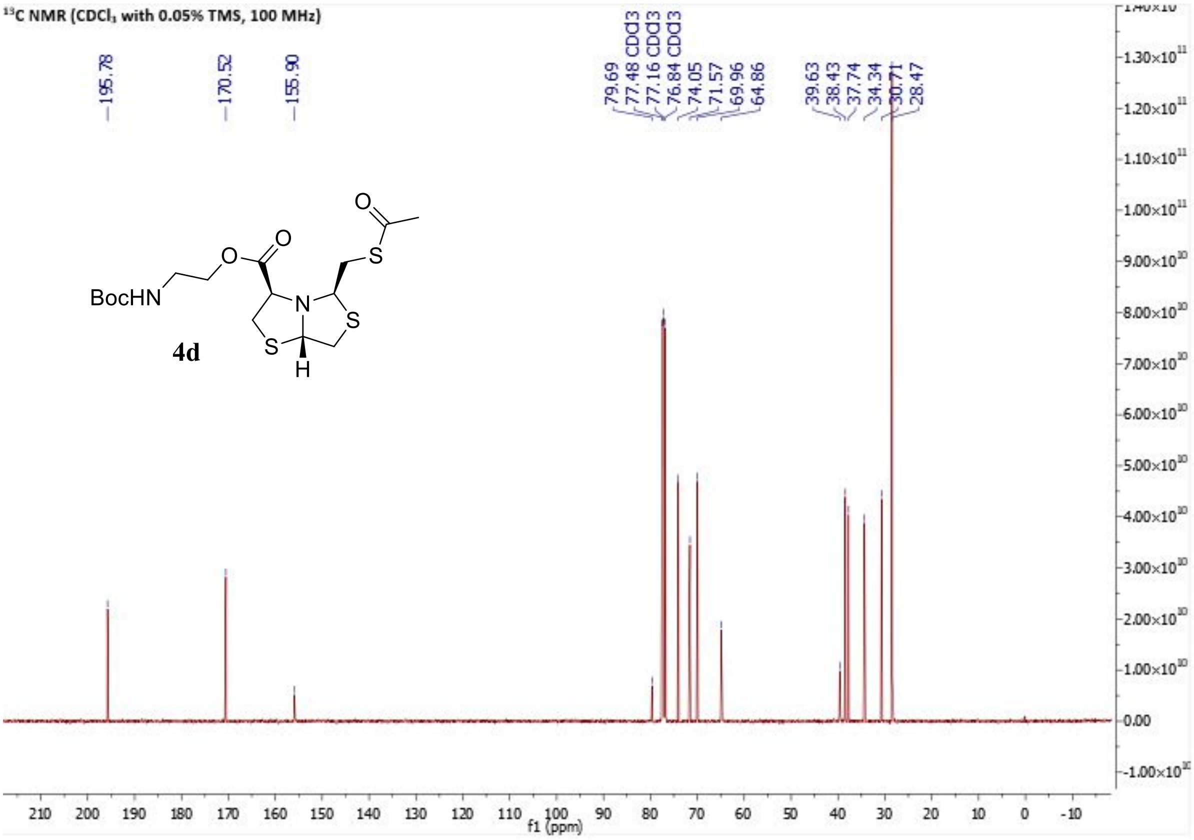

¹³C NMR (CDCl₃ with 0.05% TMS, 100 MHz)
195.78 170.52 155.90
79.69 77.48 CDCl3 77.16 CDCl3 76.84 CDCl3 74.05 71.57 69.96 64.86
39.63 38.43 37.74 34.34 30.71 28.47
BocHN
4d
f1 (ppm)

^{1}H NMR (CDCl$_3$ with 0.05% TMS, 400 MHz)

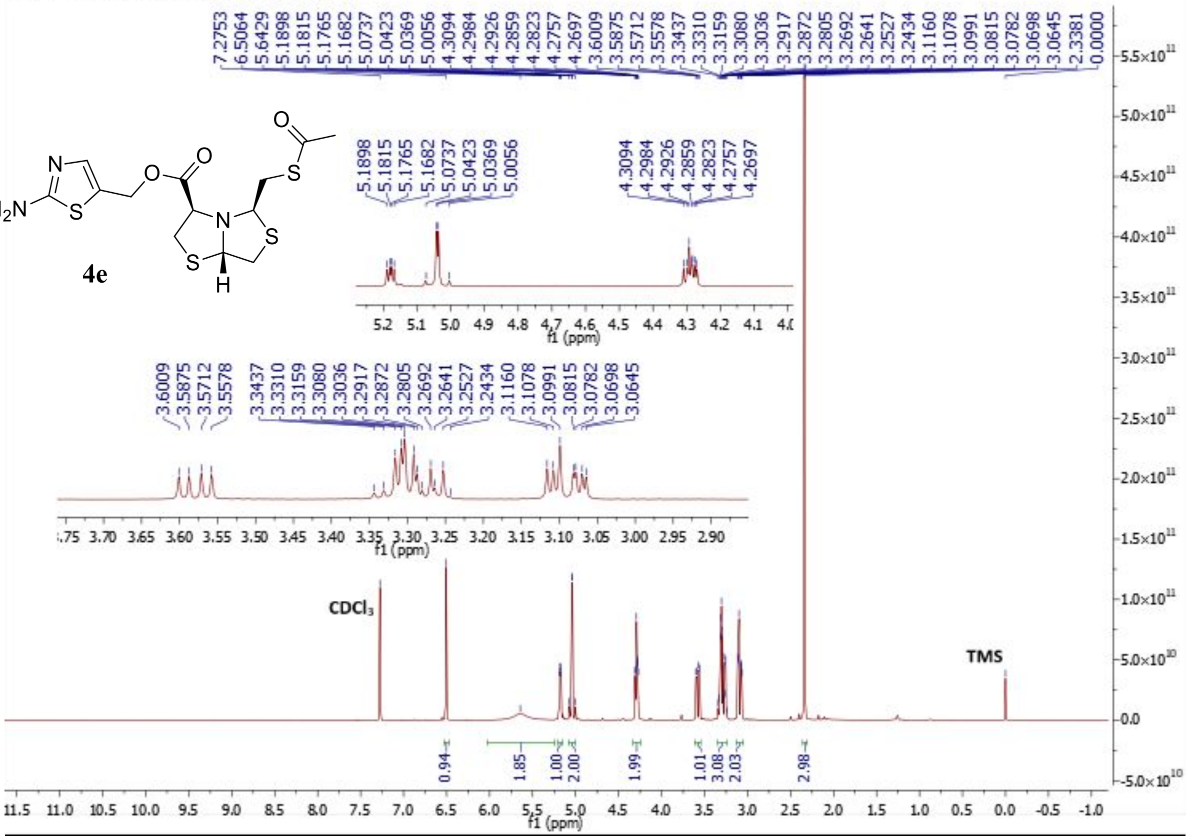

^{13}C NMR (CDCl$_3$ with 0.05% TMS, 100 MHz)

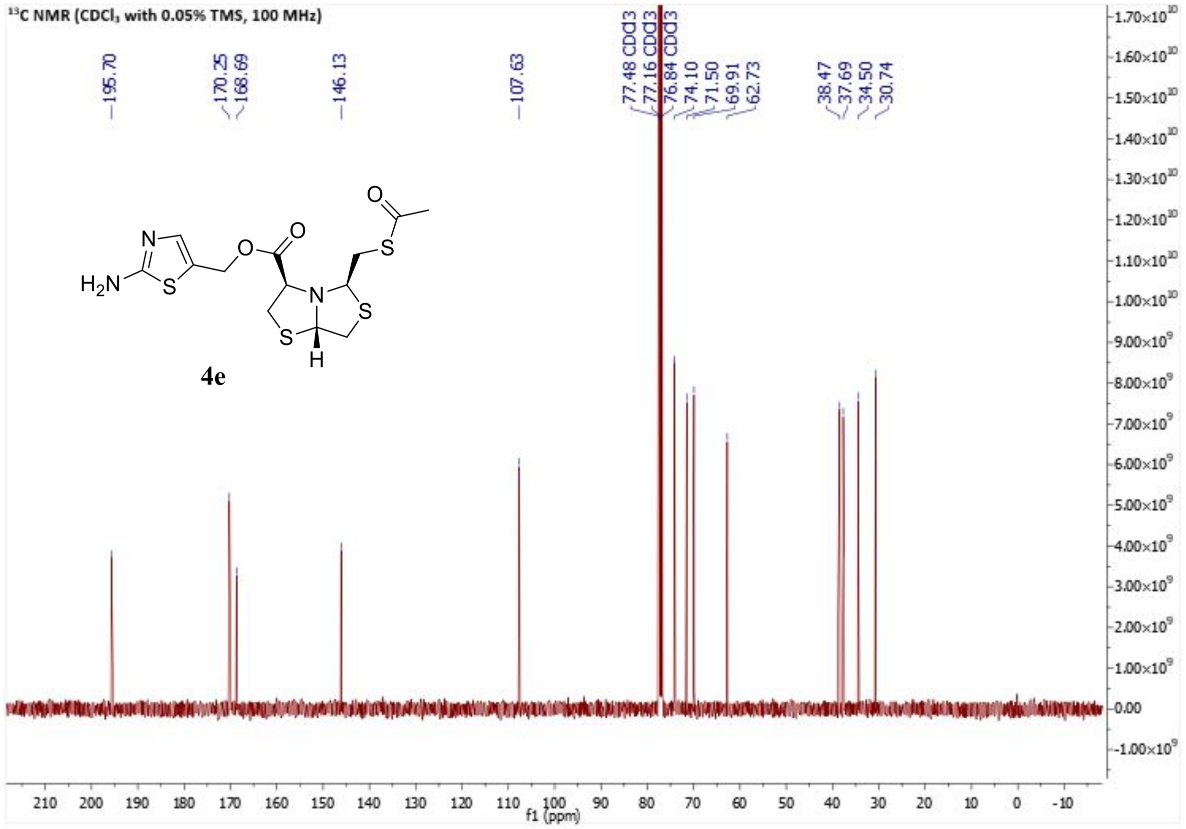

^{1}H NMR (CDCl$_3$ with 0.05% TMS, 400 MHz)

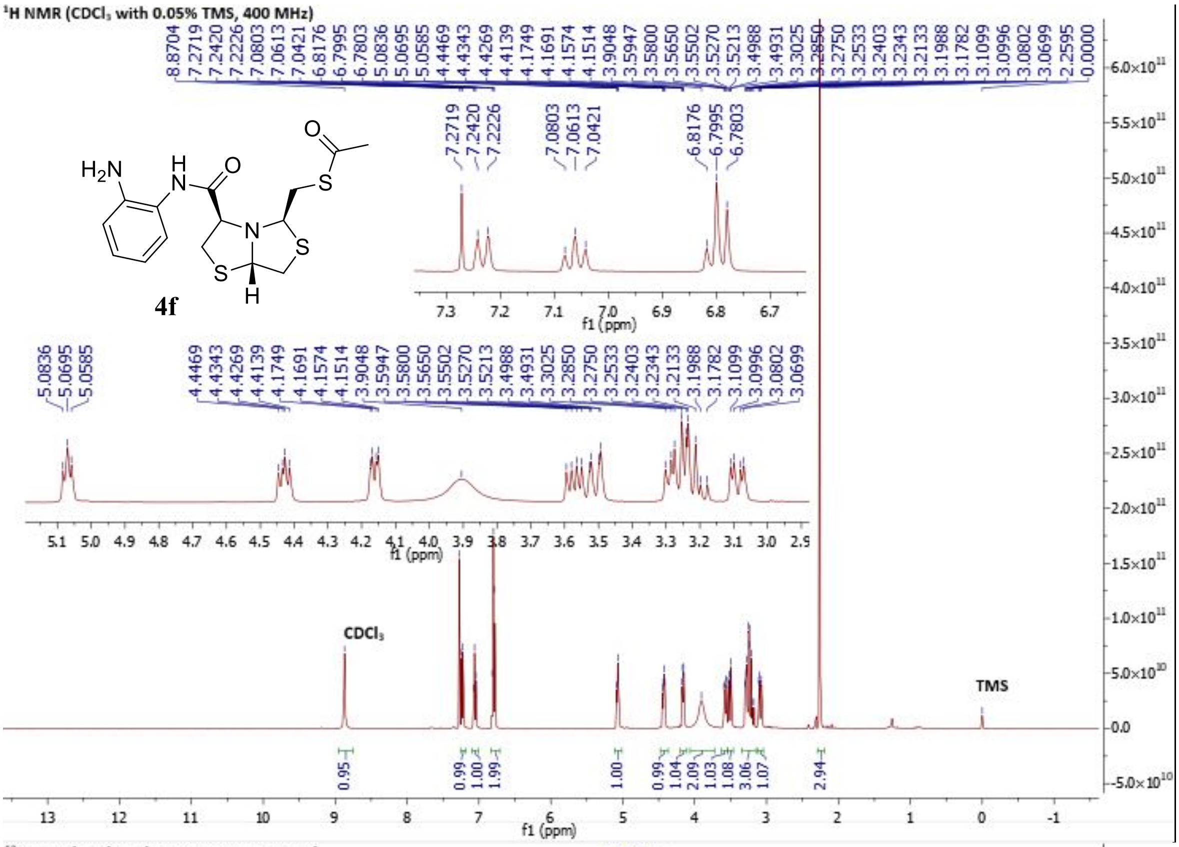

^{13}C NMR (CDCl$_3$ with 0.05% TMS, 100 MHz)

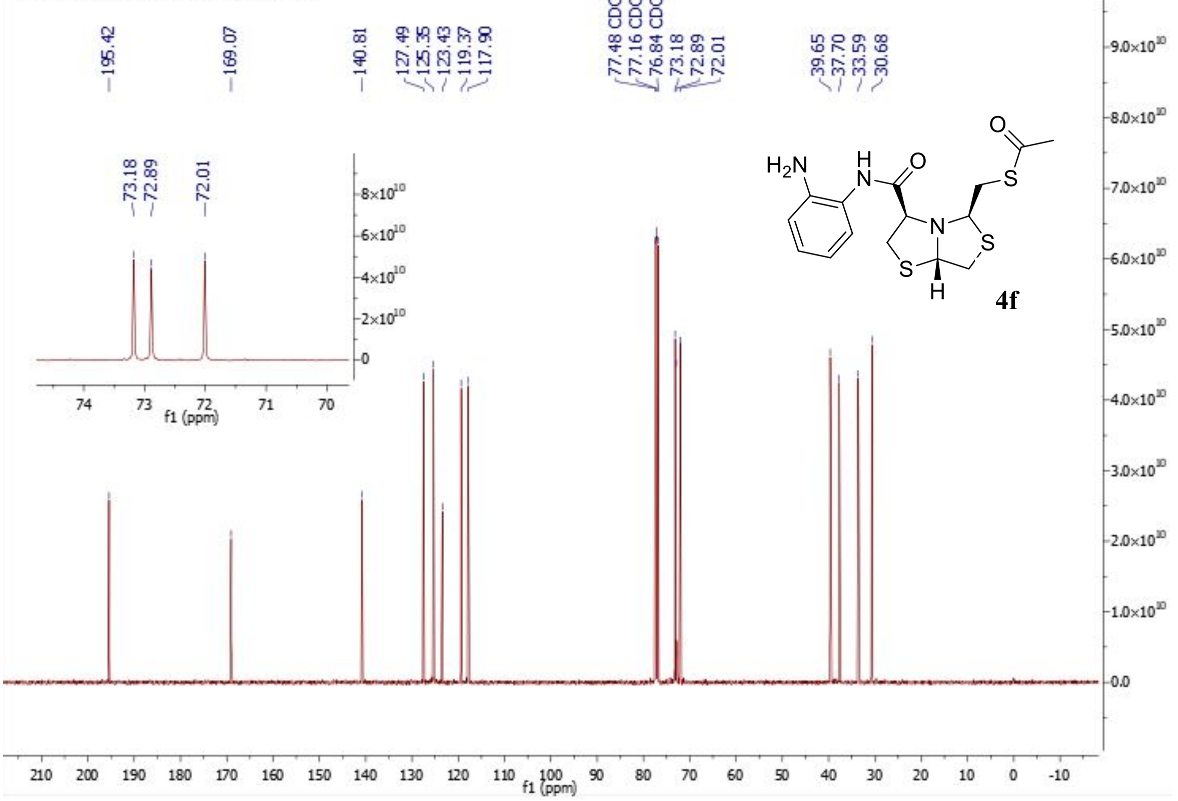

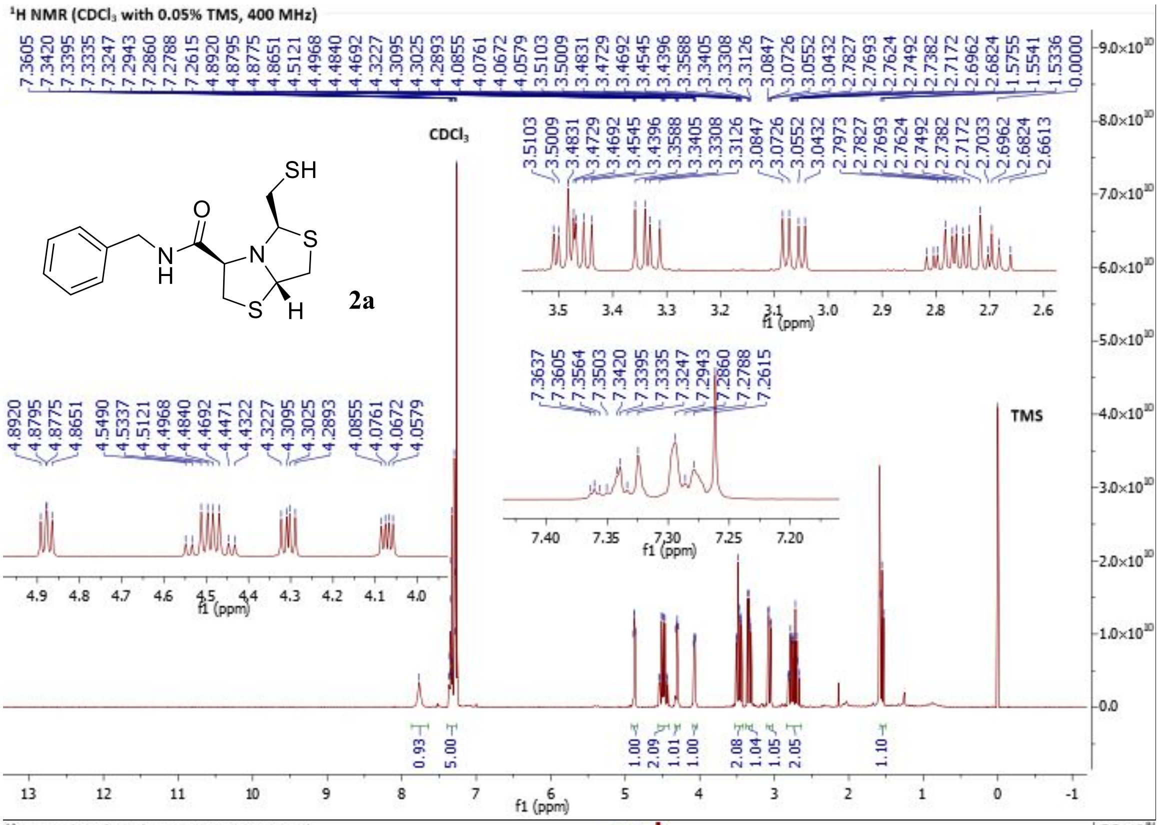

^{1}H NMR (CDCl$_3$ with 0.05% TMS, 400 MHz)
CDCl$_3$
TMS
2a
f1 (ppm)

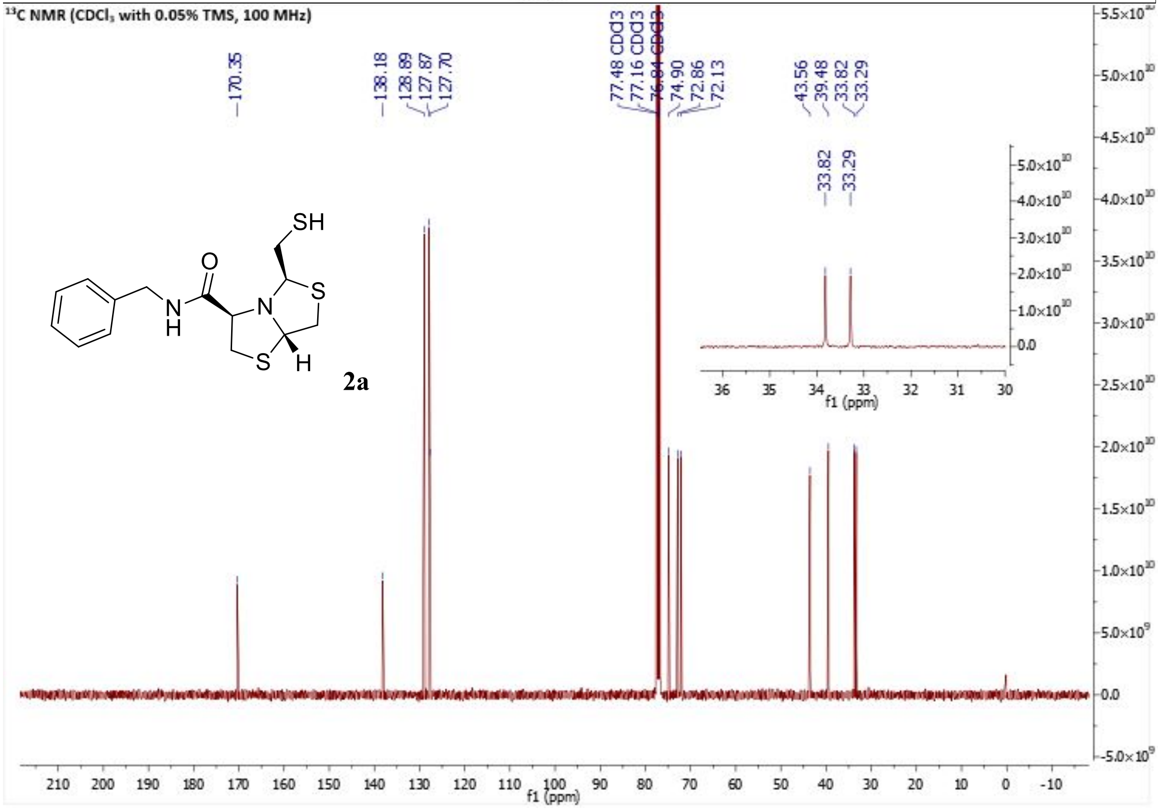

^{13}C NMR (CDCl$_3$ with 0.05% TMS, 100 MHz)
170.35
138.18
128.89
127.87
127.70
77.48 CDCl3
77.16 CDCl3
76.84 CDCl3
74.90
72.86
72.13
43.56
39.48
33.82
33.29
2a
f1 (ppm)

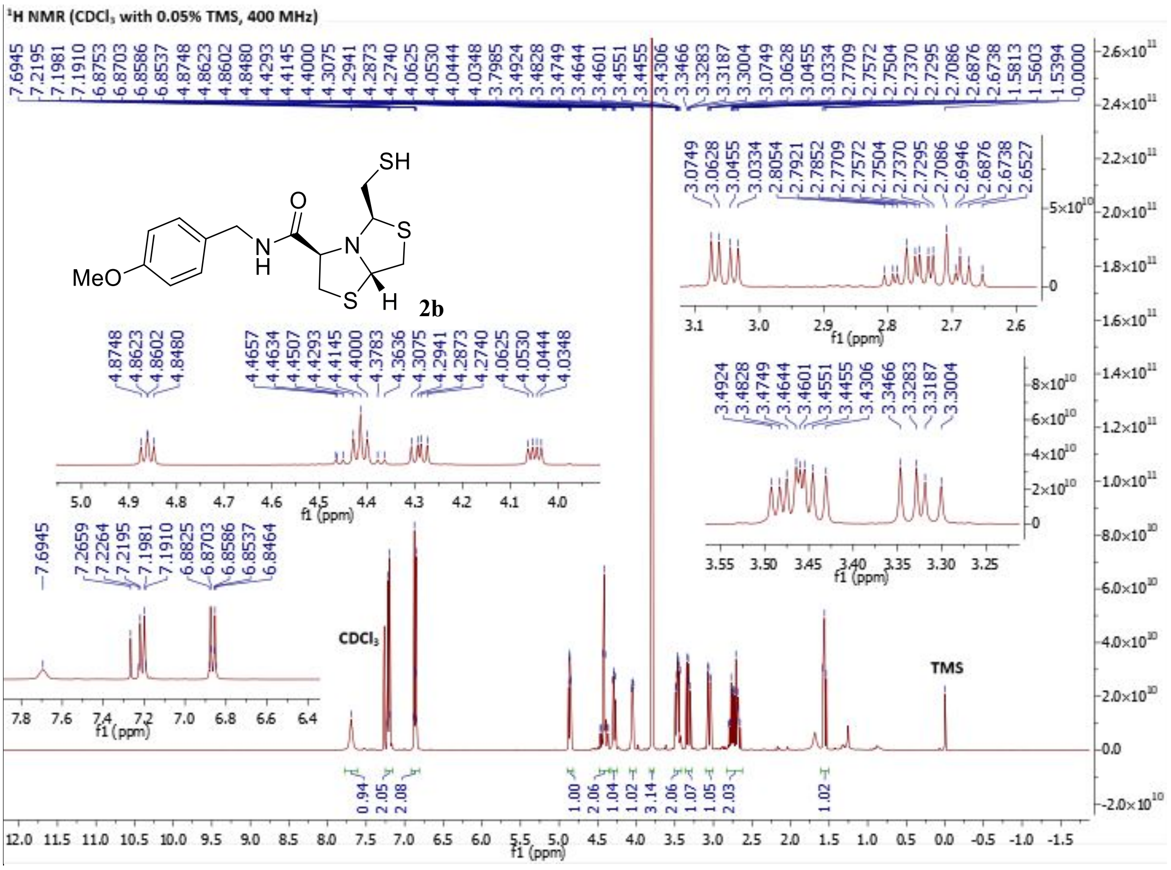
¹H NMR (CDCl₃ with 0.05% TMS, 400 MHz)
2b
CDCl₃
TMS
f1 (ppm)

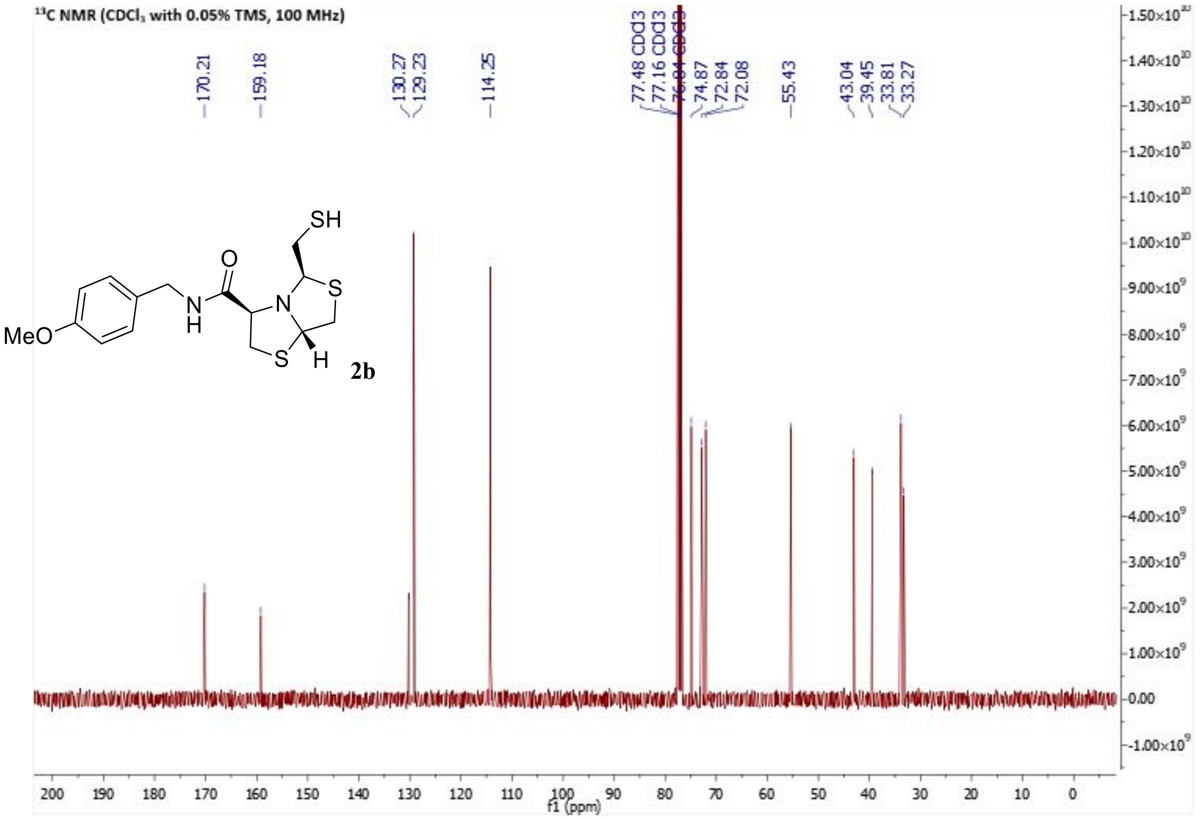
¹³C NMR (CDCl₃ with 0.05% TMS, 100 MHz)
170.21
159.18
130.27
129.23
114.25
77.48 CDCl3
77.16 CDCl3
74.87
72.84
72.08
55.43
43.04
39.45
33.81
33.27
2b
f1 (ppm)

^{1}H NMR ($CDCl_3$ with 0.05% TMS, 400 MHz)

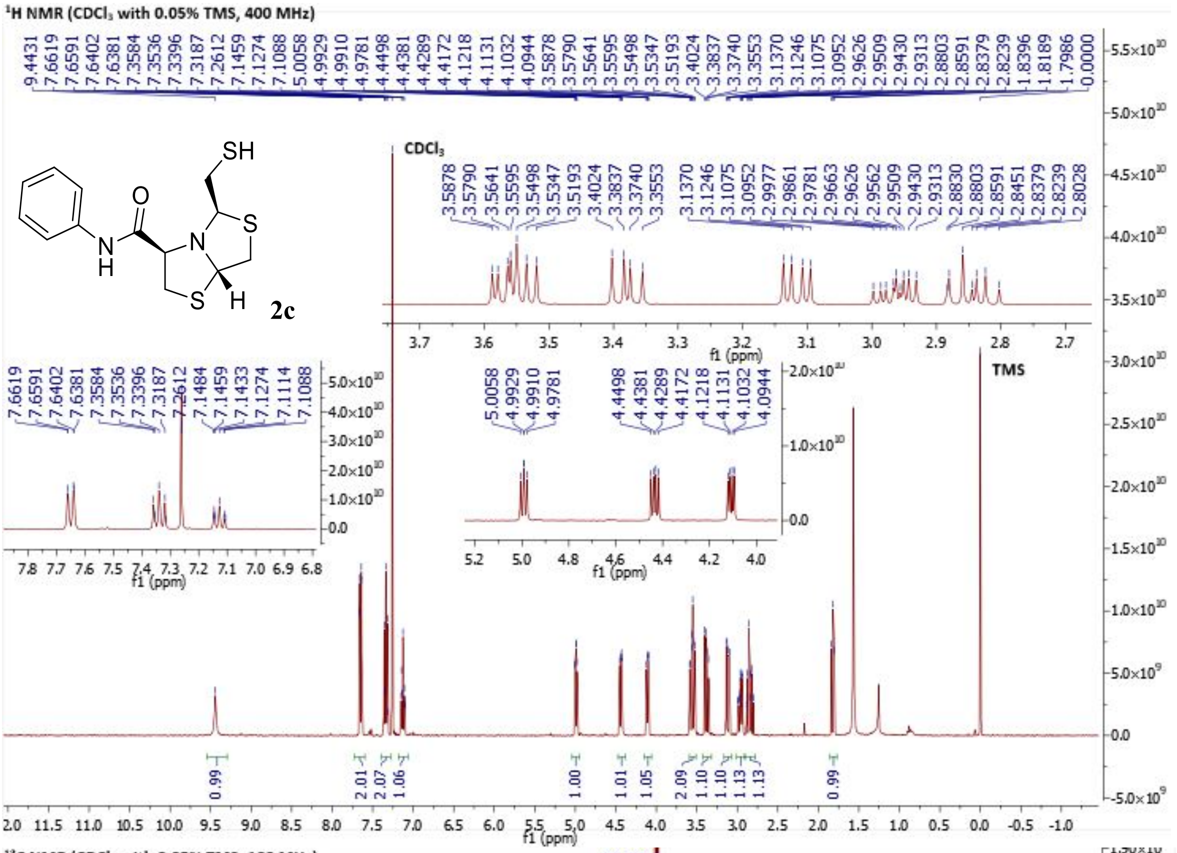

^{13}C NMR ($CDCl_3$ with 0.05% TMS, 100 MHz)

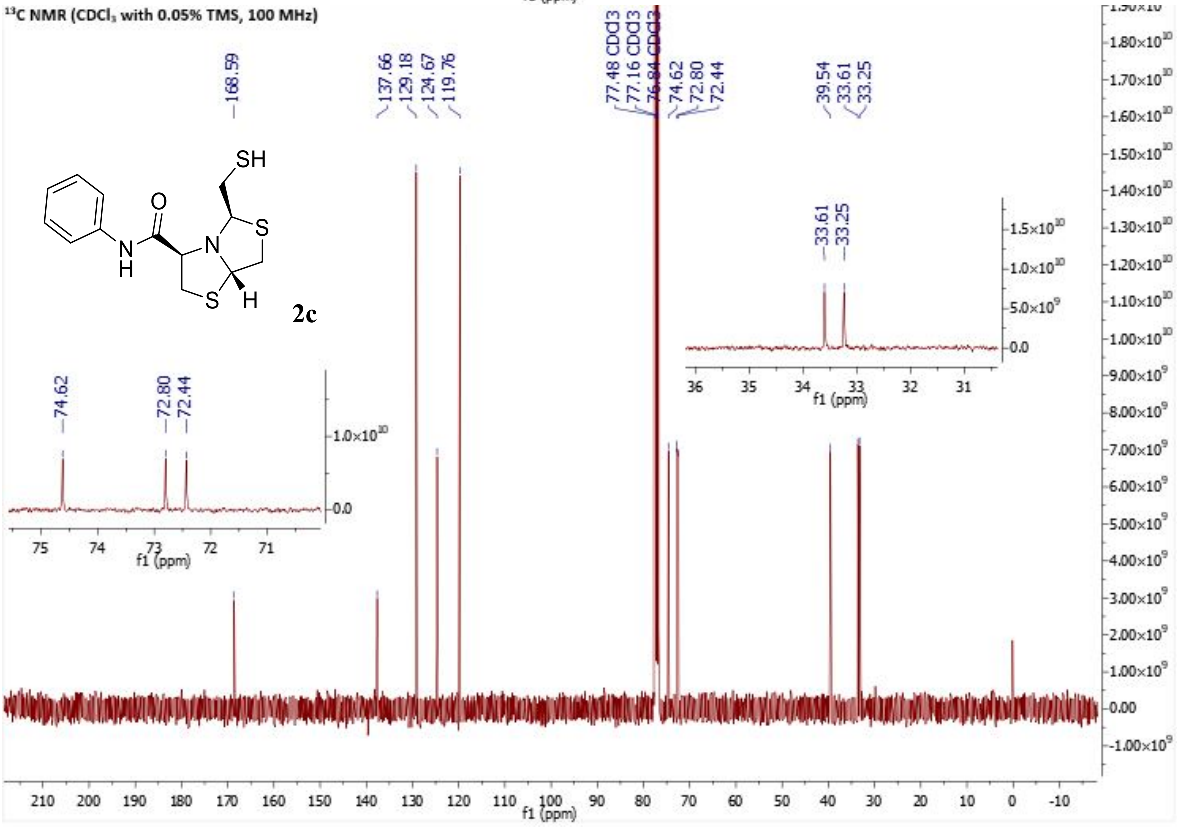

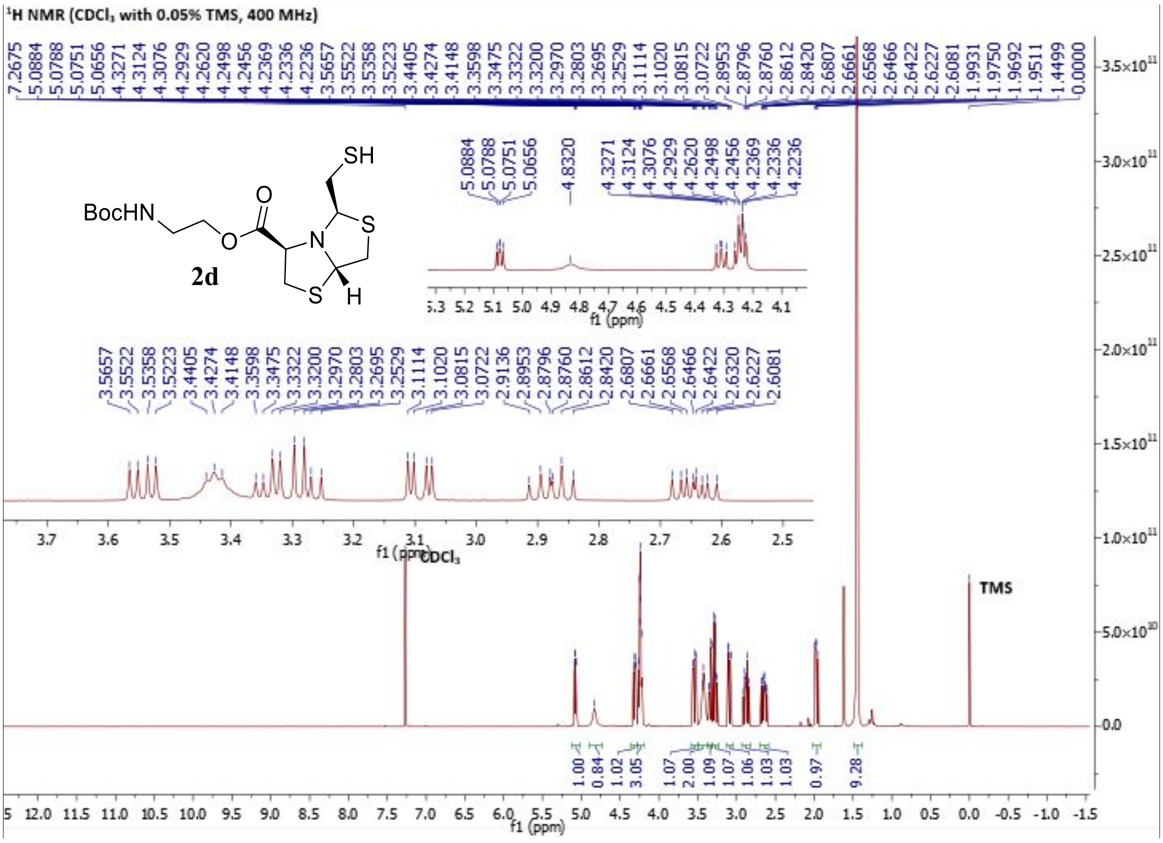
¹H NMR (CDCl₃ with 0.05% TMS, 400 MHz)
BocHN
SH
O
N
S
H
2d
CDCl₃
TMS
f1 (ppm)

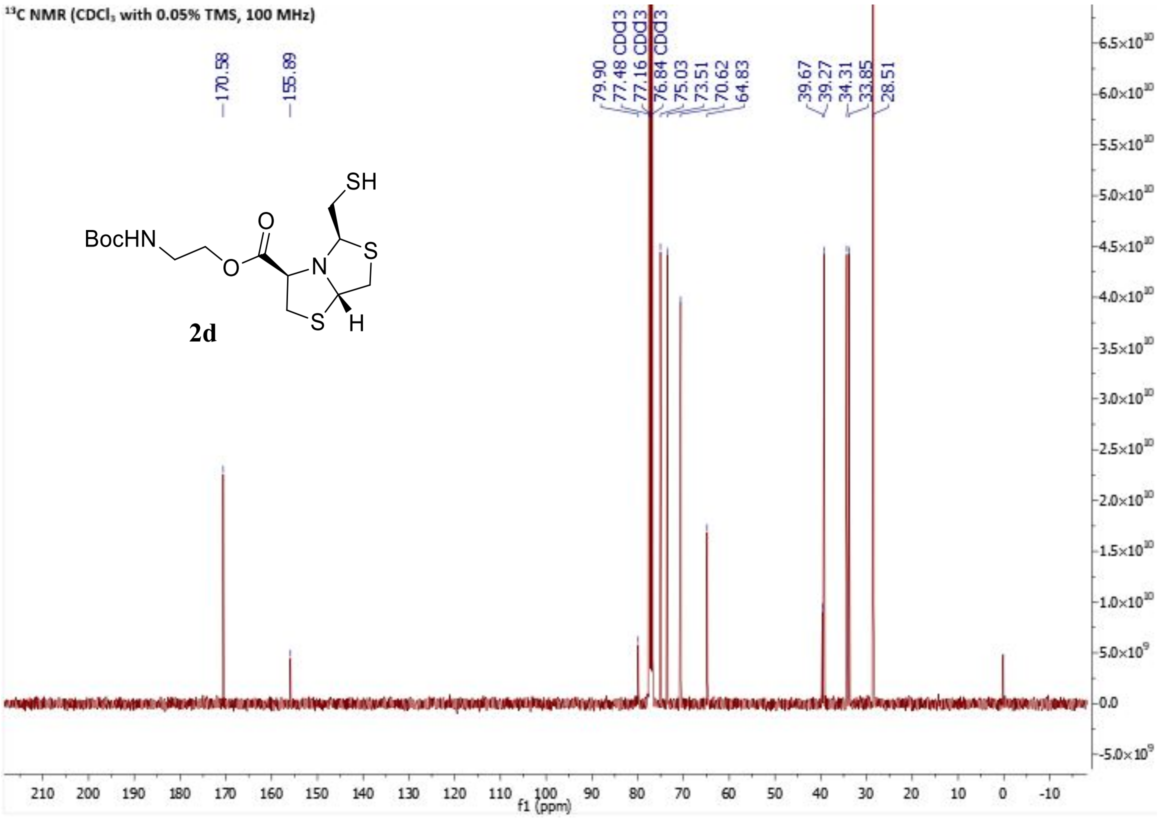
¹³C NMR (CDCl₃ with 0.05% TMS, 100 MHz)
170.58
155.89
79.90
77.48 CDCl3
77.16 CDCl3
76.84 CDCl3
75.03
73.51
70.62
64.83
39.67
39.27
34.31
33.85
28.51
BocHN
SH
O
N
S
H
2d
f1 (ppm)

^{1}H NMR ($CDCl_3$ with 0.05% TMS, 400 MHz)

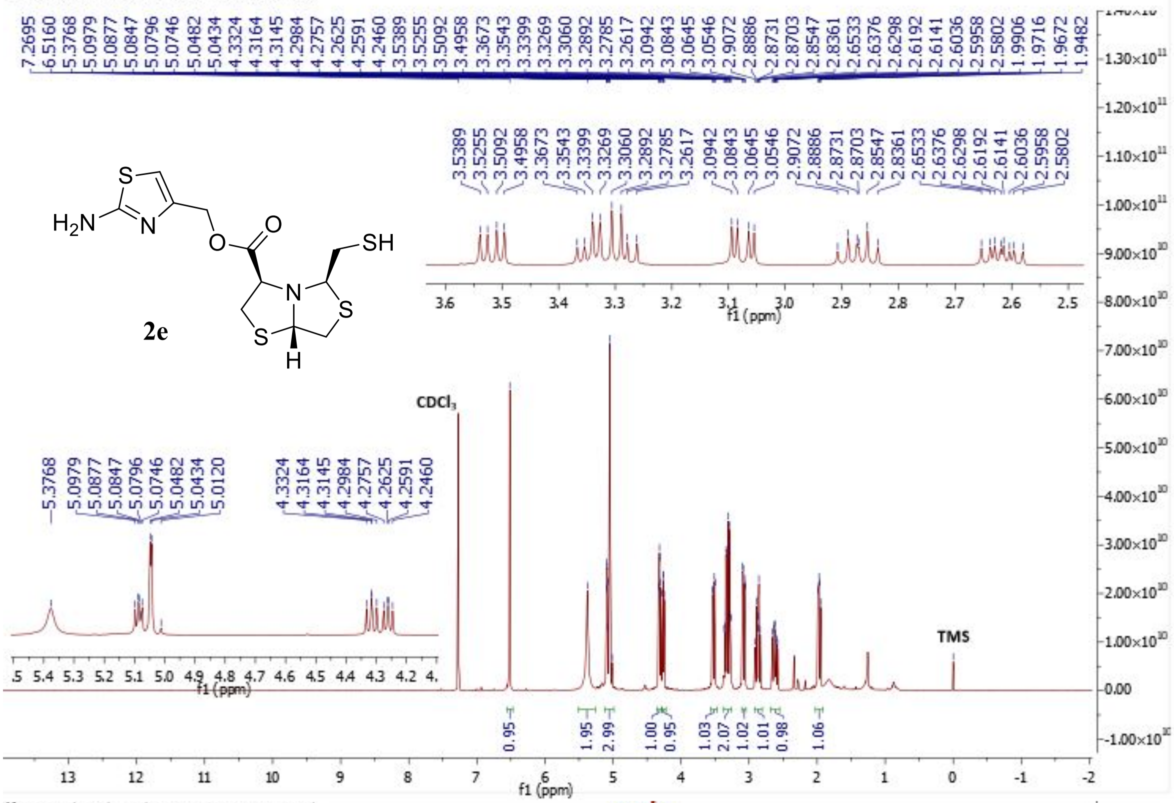

^{13}C NMR ($CDCl_3$ with 0.05% TMS, 100 MHz)

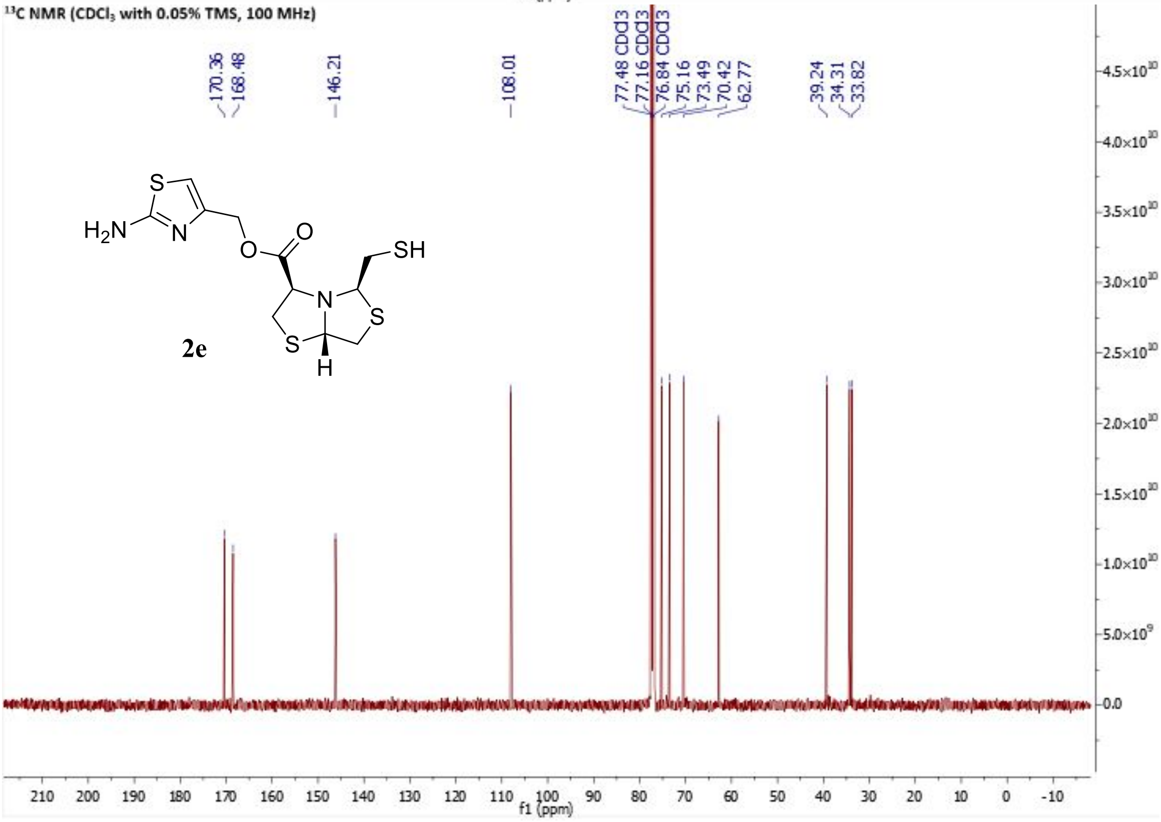

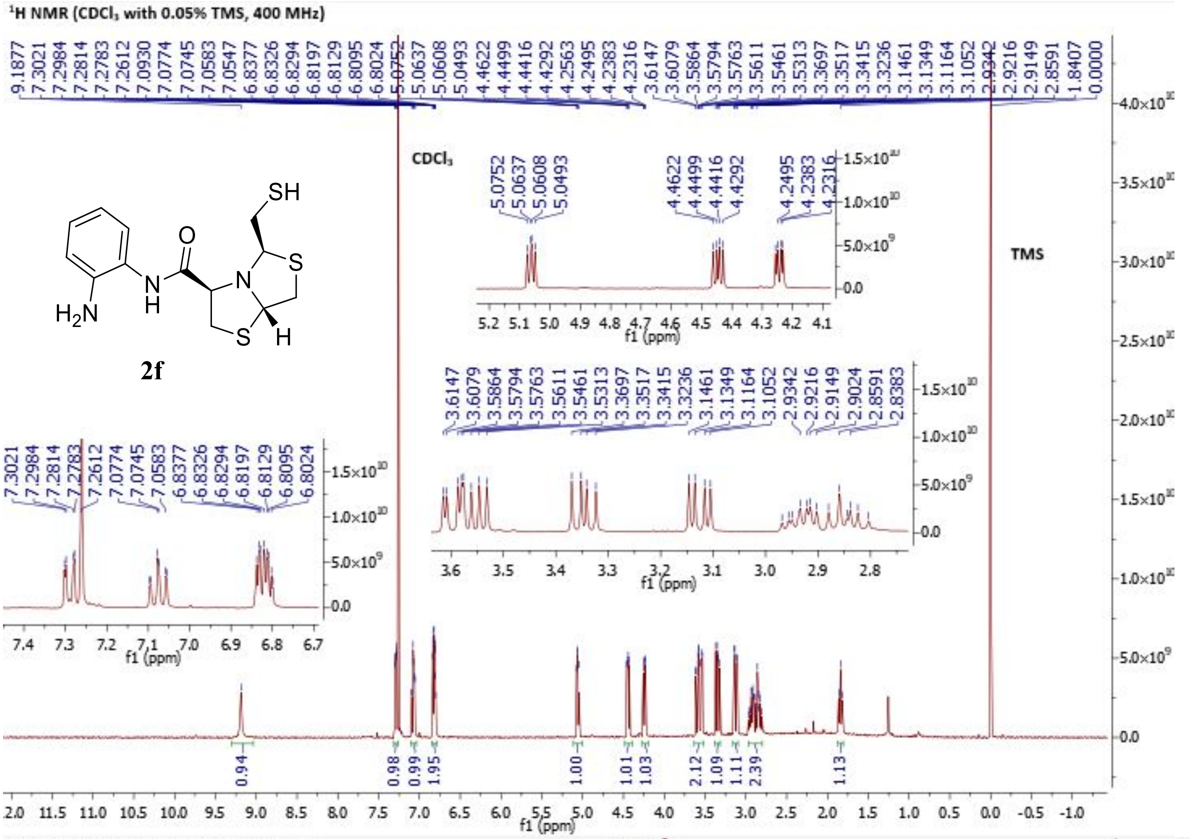
1H NMR (CDCl3 with 0.05% TMS, 400 MHz)
CDCl3
TMS
SH
O
N
S
H2N
H
2f
f1 (ppm)

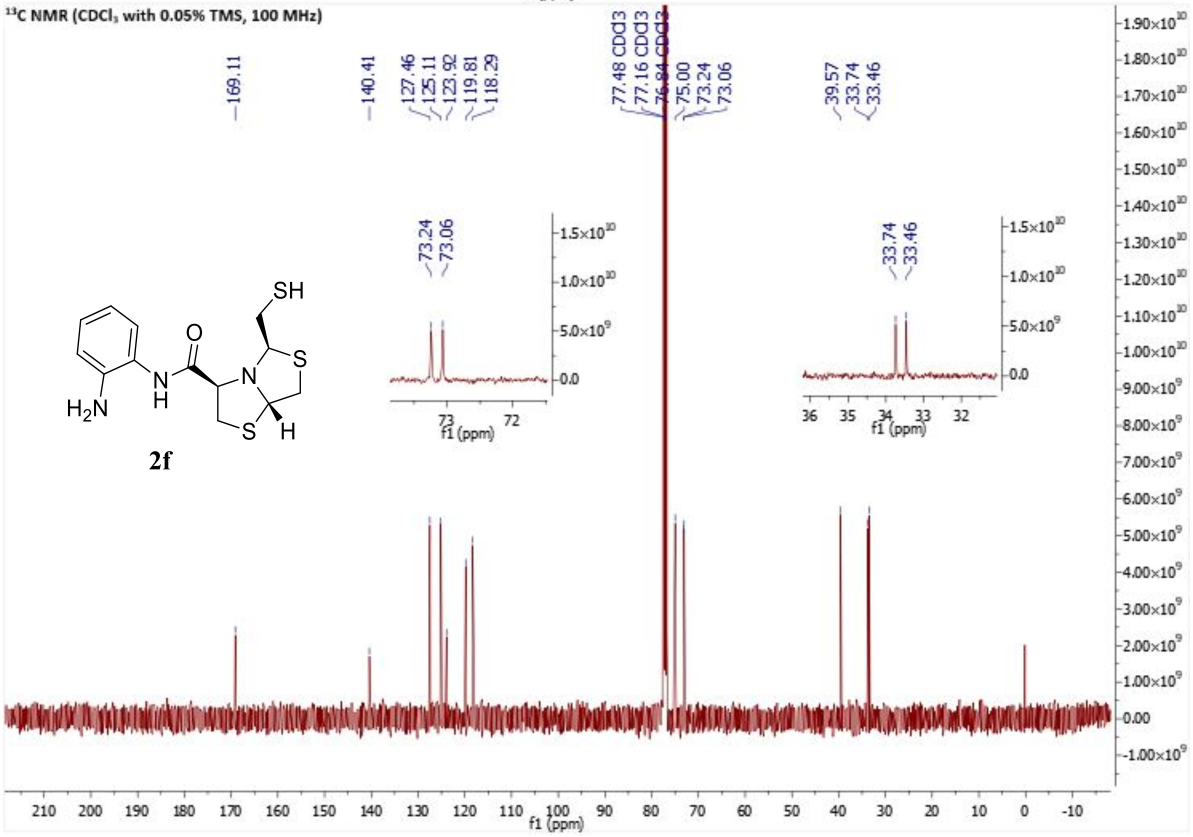
13C NMR (CDCl3 with 0.05% TMS, 100 MHz)
169.11
140.41
127.46
125.11
123.92
119.81
118.29
77.48 CDCl3
77.16 CDCl3
76.84 CDCl3
75.00
73.24
73.06
39.57
33.74
33.46
2f
f1 (ppm)

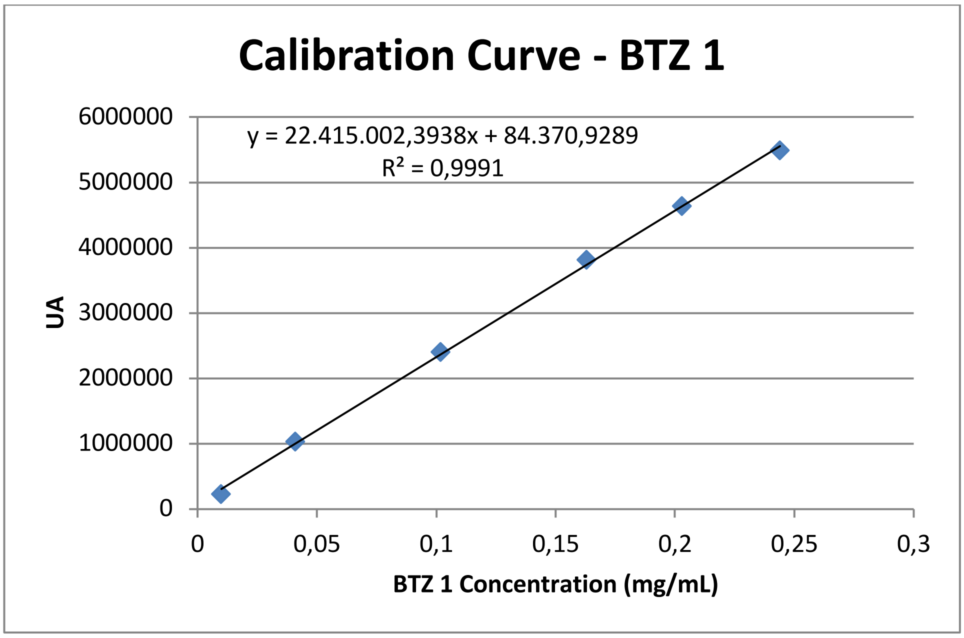

Calibration curve for the quantification of BTZ 1. Concentration range: 0.01-0.244 mg/mL.

HPLC chromatograms – Method validation of BTZ **1**

Concentration Level: 0.01 mg/mL

Empower 3 SOFTWARE

Purezas

SAMPLE INFORMATION			
Sample Name:	Std 5% inj 1, Std 5% inj 2	Acquired By:	System
Sample Type:		Sample Set Name:	
Vial:		Acq. Method Set:	Tioester exchange
Injection #:		Processing Method:	Tiolexchange
Injection Volume:		Channel Name:	205,0nm
Run Time:	9,0 Minutes	Proc. Chnl. Descr.:	PDA 205,0 nm (PDA 190.0 to 400.0 nm at 1.2 nm)
Date Acquired:	07/02/2020 17:25:52 UYST, 07/02/2020 17:35:44 UYST		
Date Processed:			

Auto-Scaled Chromatogram

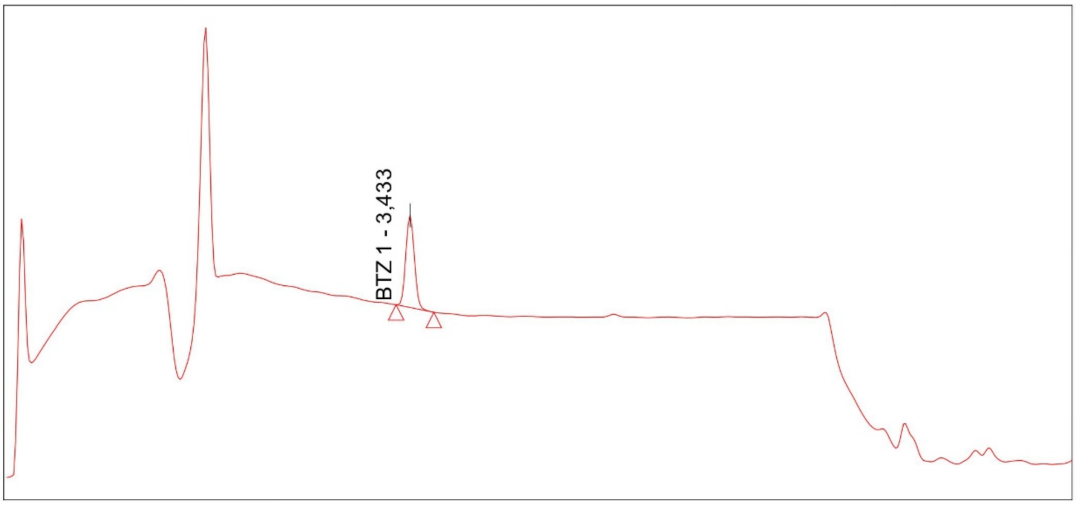

SampleName Std 5% inj 1; Date Acquired 07/02/2020 17:25:52 UYST

Reported by User: System
Report Method: Purezas
Report Method ID: [illegible]
Page: 1 of 3

Project Name: Valentina y Graciela
Date Printed:
19/05/2022
17:41:26 America/Montevideo

Auto-Scaled Chromatogram

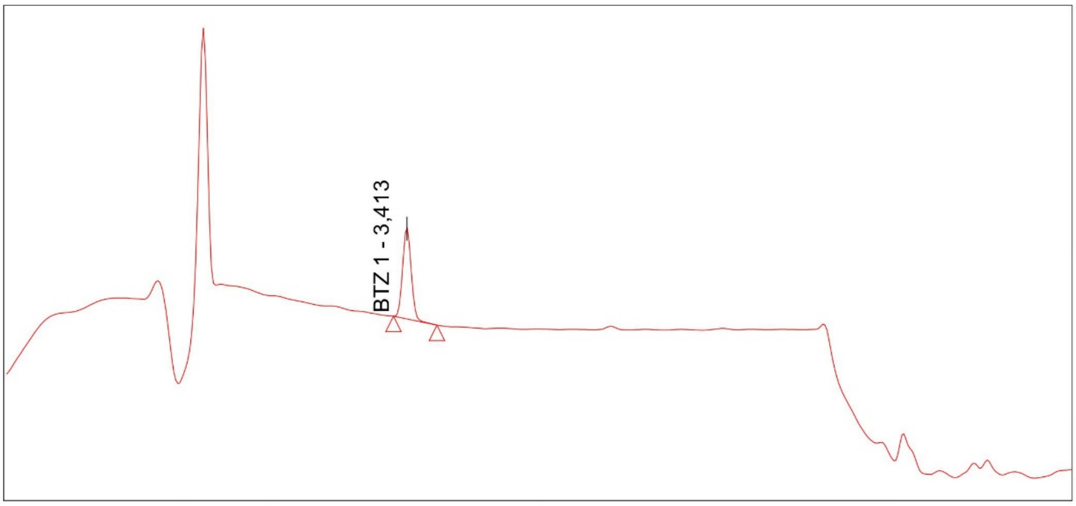

SampleName Std 5% inj 2; Date Acquired 07/02/2020 17:35:44 UYST

Peak Results

	Name	RT	Area	Height	Injection	% Area
1	BTZ 1	3,433	235238	46706	32	100,00
2	BTZ 1	3,413	230439	45692	33	100,00
Mean			232838,5			
% RSD			1,5			

Spectrum Index Fraction Plot

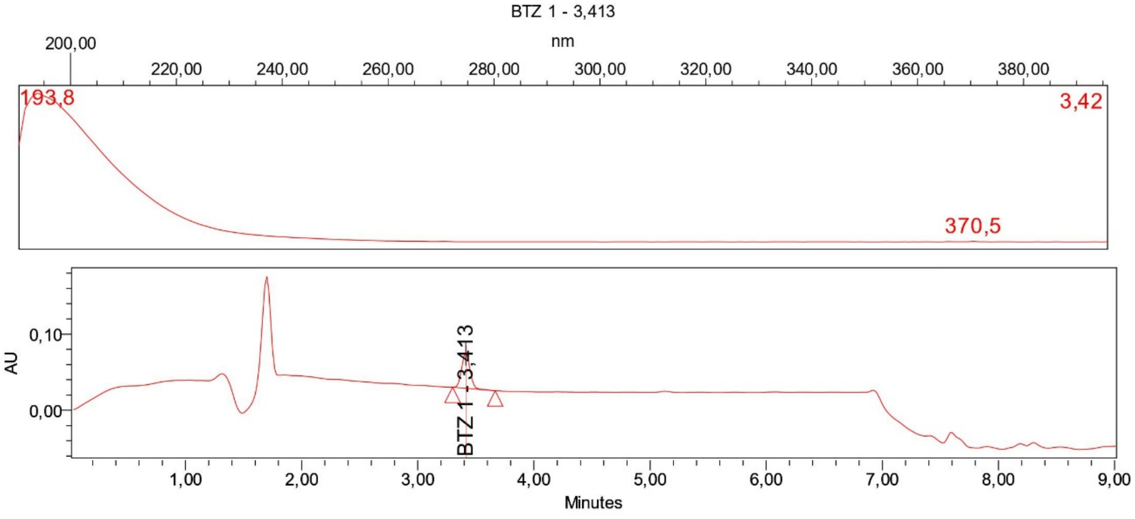

Spectrum Index Fraction Plot

BTZ 1 - 3,433

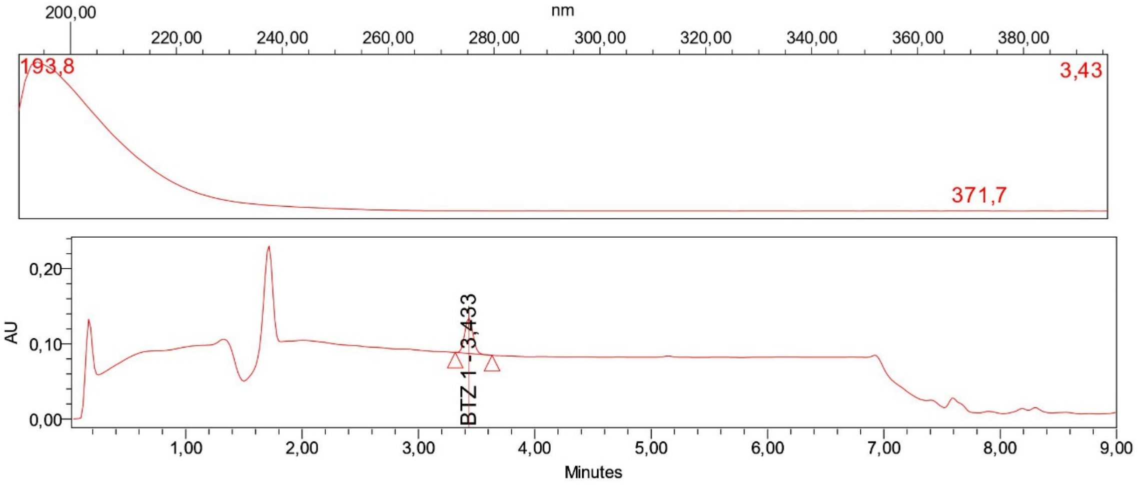

Reported by User: System
Report Method: Purezas
Report Method ID: [illegible]
Page: 3 of 3

Project Name: Valentina y Graciela
Date Printed:
19/05/2022
17:41:26 America/Montevideo

Concentration Level: 0.041 mg/mL

Purezas

SAMPLE INFORMATION

Sample Name:	Std 20% inj 2, Std 20% inj 1	Acquired By:	System
Sample Type:		Sample Set Name:	
Vial:		Acq. Method Set:	Tioester exchange
Injection #:		Processing Method:	Tiolexchange
Injection Volume:		Channel Name:	205,0nm
Run Time:	9,0 Minutes	Proc. Chnl. Descr.:	PDA 205,0 nm (PDA 190.0 to 400.0 nm at 1.2 nm)
Date Acquired:	07/02/2020 17:06:05 UYST, 07/02/2020 17:16:14 UYST		
Date Processed:			

Auto-Scaled Chromatogram

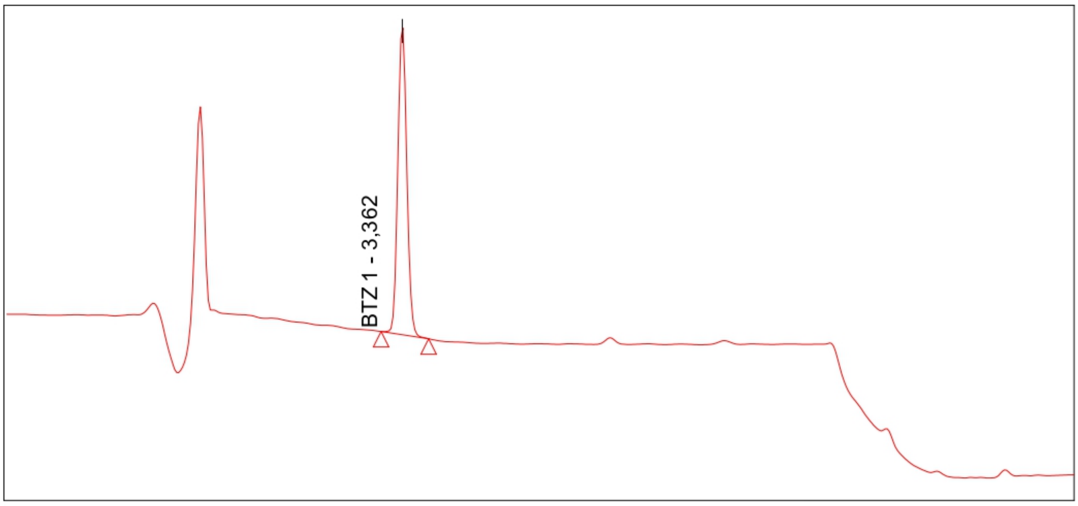

SampleName Std 20% inj 1; Date Acquired 07/02/2020 17:06:05 UYST

Reported by User: System
Report Method: Purezas
Report Method ID: 46618
Page: 1 of 3

Project Name: Valentina y Graciela
Date Printed:
19/05/2022
17:41:45 America/Montevideo

Auto-Scaled Chromatogram

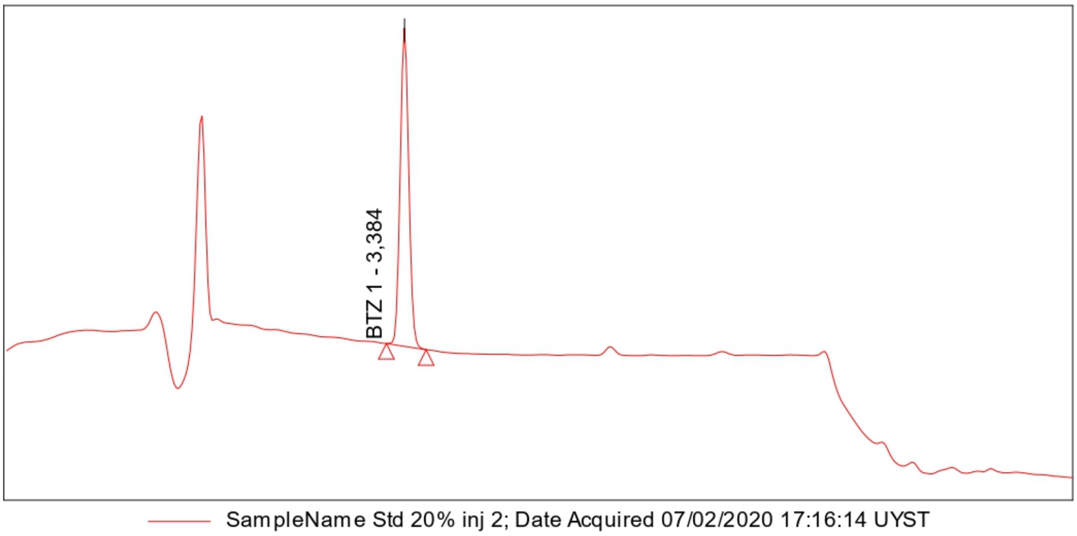

Peak Results

	Name	RT	Area	Height	Injection	% Area
1	BTZ 1	3,362	1037702	197003	30	100,00
2	BTZ 1	3,384	1034245	203520	31	100,00
Mean			1035973,4			
% RSD			0,2			

Spectrum Index Fraction Plot

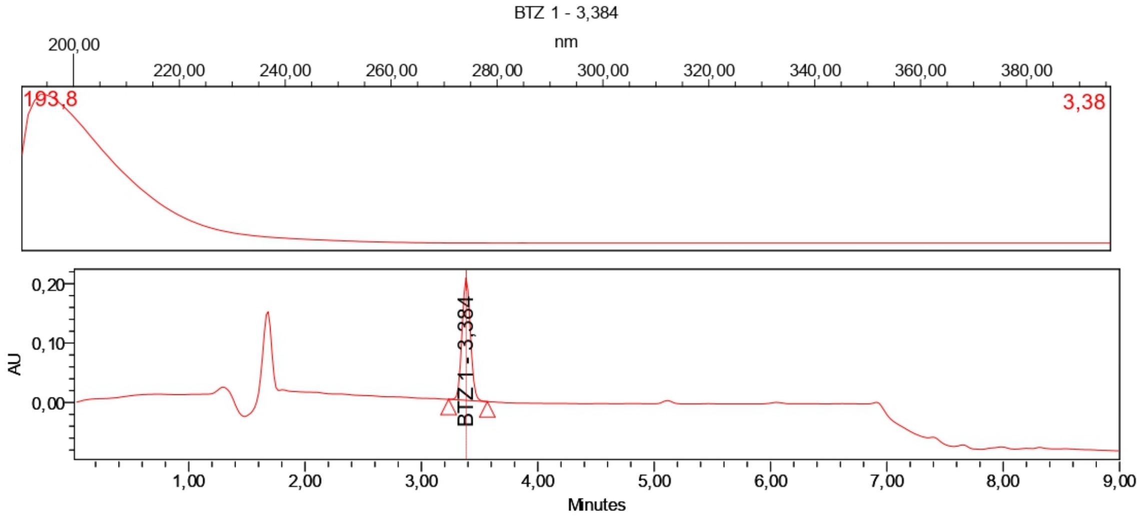

Reported by User: System
Report Method: Purezas
Report Method ID:46618
Page: 2 of 3

Project Name: Valentina y Graciela
Date Printed:
19/05/2022
17:41:45 America/Montevideo

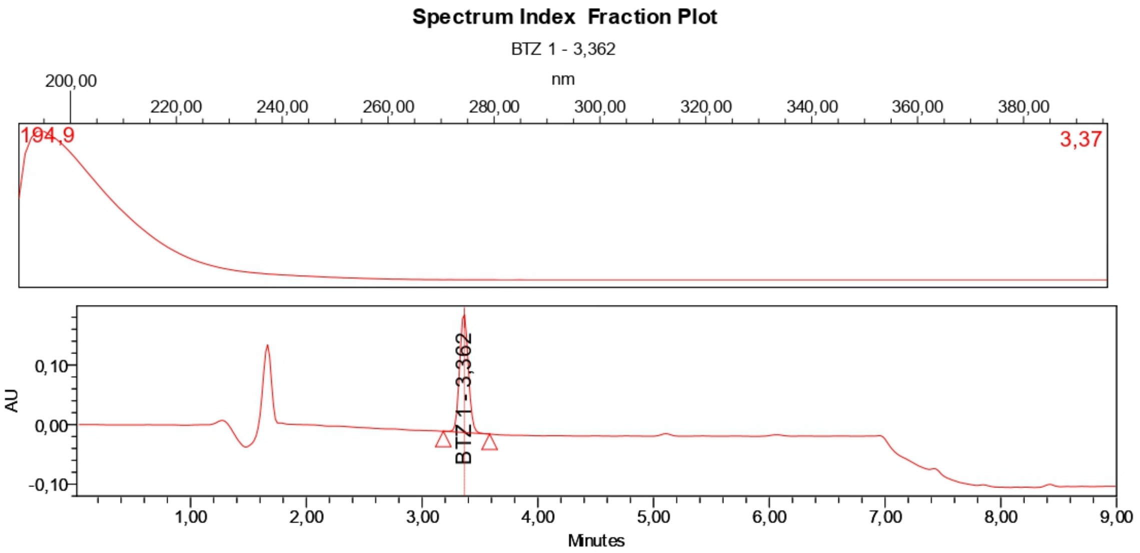

Reported by User: System
Report Method: Purezas
Report Method ID: [illegible]
Page: 3 of 3

Project Name: Valentina y Graciela
Date Printed:
19/05/2022
17:41:45 America/Montevideo

Concentration Level: 0.102 mg/mL

Empower 3 SOFTWARE

Purezas

SAMPLE INFORMATION

Sample Name:	Std 50% inj 1, Std 50% inj 2	Acquired By:	System
Sample Type:		Sample Set Name:	
Vial:		Acq. Method Set:	Tioester exchange
Injection #:		Processing Method:	Tiolexchange
Injection Volume:		Channel Name:	205,0nm
Run Time:	9,0 Minutes	Proc. Chnl. Descr.:	PDA 205,0 nm (PDA 190.0 to 400.0 nm at 1.2 nm)
Date Acquired:	07/02/2020 16:43:45 UYST, 07/02/2020 16:53:58 UYST		
Date Processed:			

Auto-Scaled Chromatogram

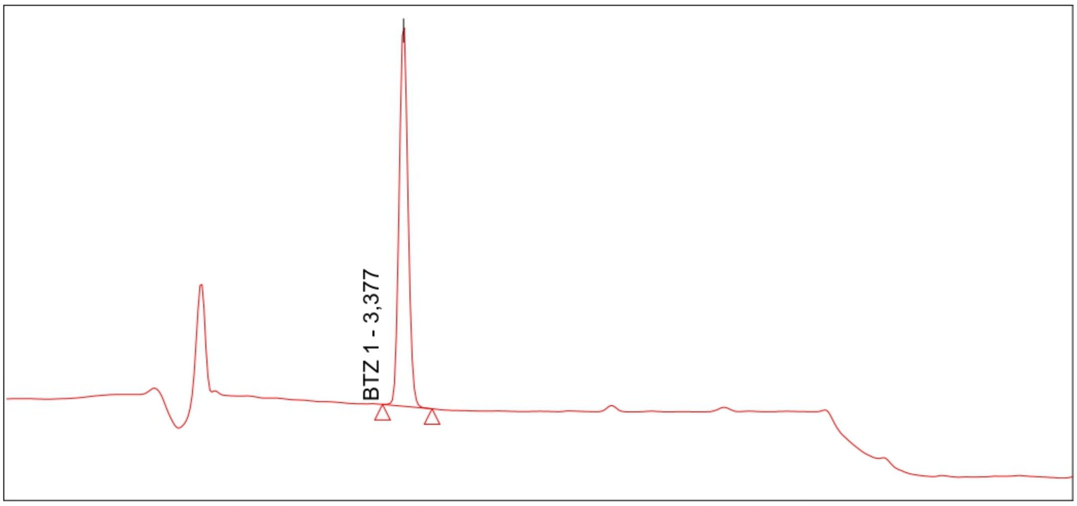

SampleName Std 50% inj 1; Date Acquired 07/02/2020 16:43:45 UYST

Reported by User: System
Report Method: Purezas
Report Method ID: 46618
Page: 1 of 3

Project Name: Valentina y Graciela
Date Printed:
19/05/2022
17:42:01 America/Montevideo

Auto-Scaled Chromatogram

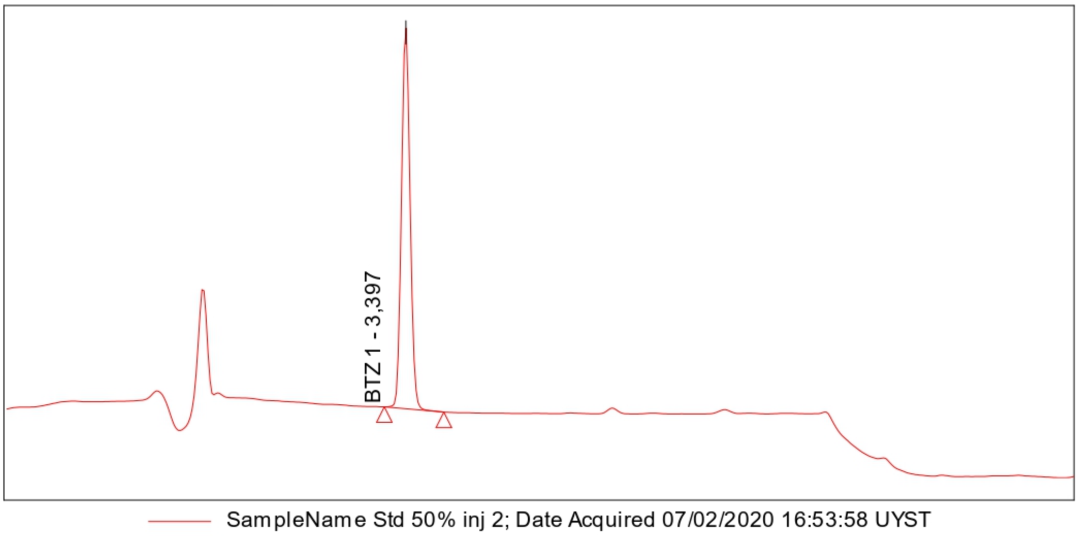

Peak Results

	Name	RT	Area	Height	Injection	% Area
1	BTZ 1	3,377	2396061	457250	28	100,00
2	BTZ 1	3,397	2416039	473769	29	100,00
Mean			2406049,9			
% RSD			0,6			

Spectrum Index Fraction Plot

BTZ 1 - 3,377

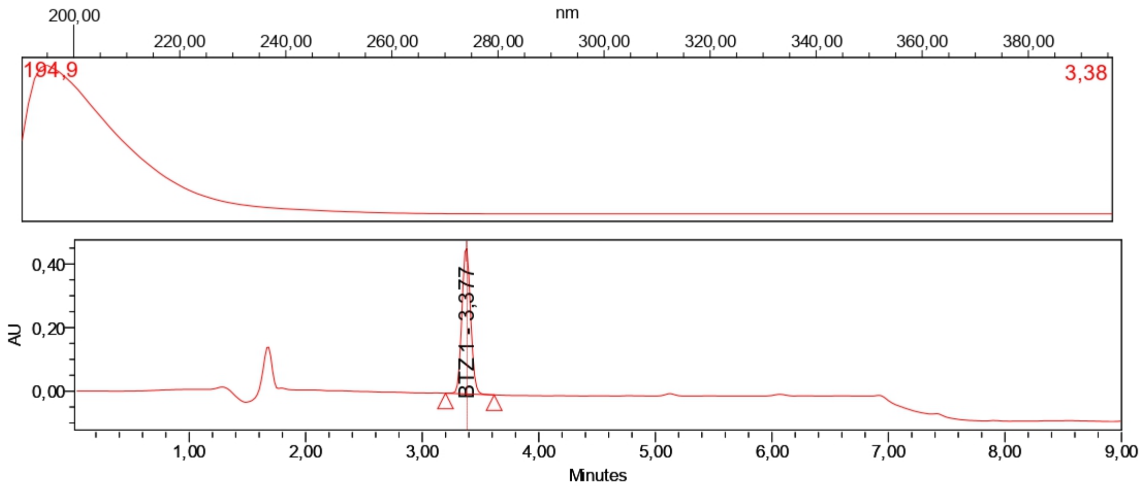

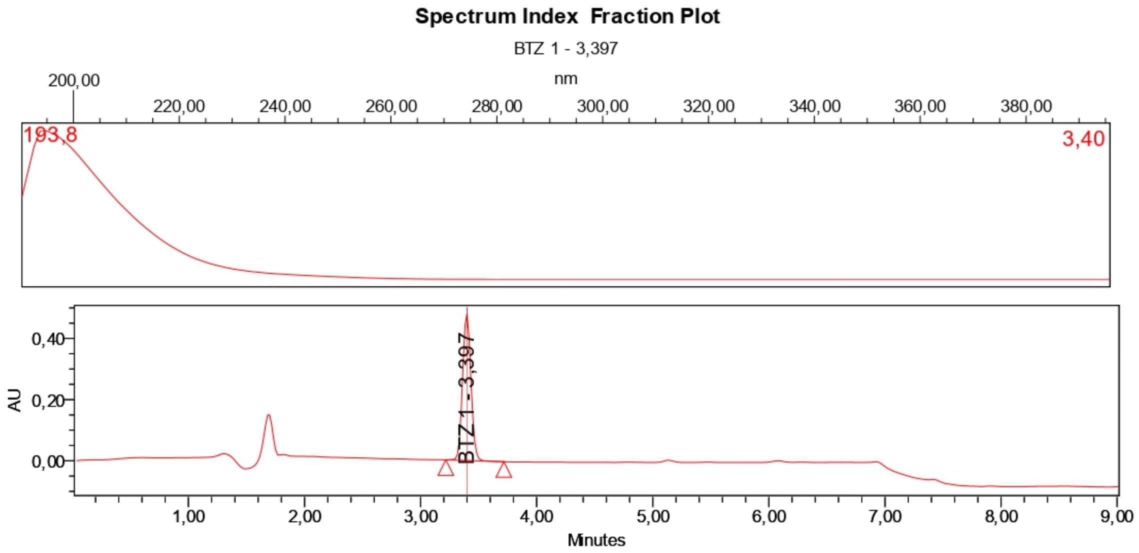

Reported by User: System
Report Method: Purezas
Report Method ID: 4618
Page: 3 of 3

Project Name: Valentina y Graciela
Date Printed:
19/05/2022
17:42:01 America/Montevideo

Concentration Level: 0.163 mg/mL

Purezas

SAMPLE INFORMATION

Sample Name:	Std 80% inj 1, Std 80% inj 2	Acquired By:	System
Sample Type:		Sample Set Name:	
Vial:		Acq. Method Set:	Tioester exchange
Injection #:		Processing Method:	Tiolexchange
Injection Volume:		Channel Name:	205,0nm
Run Time:	9,0 Minutes	Proc. Chnl. Descr.:	PDA 205,0 nm (PDA 190.0 to 400.0 nm at 1.2 nm)
Date Acquired:	07/02/2020 17:45:45 UYST, 07/02/2020 17:55:25 UYST		
Date Processed:			

Auto-Scaled Chromatogram

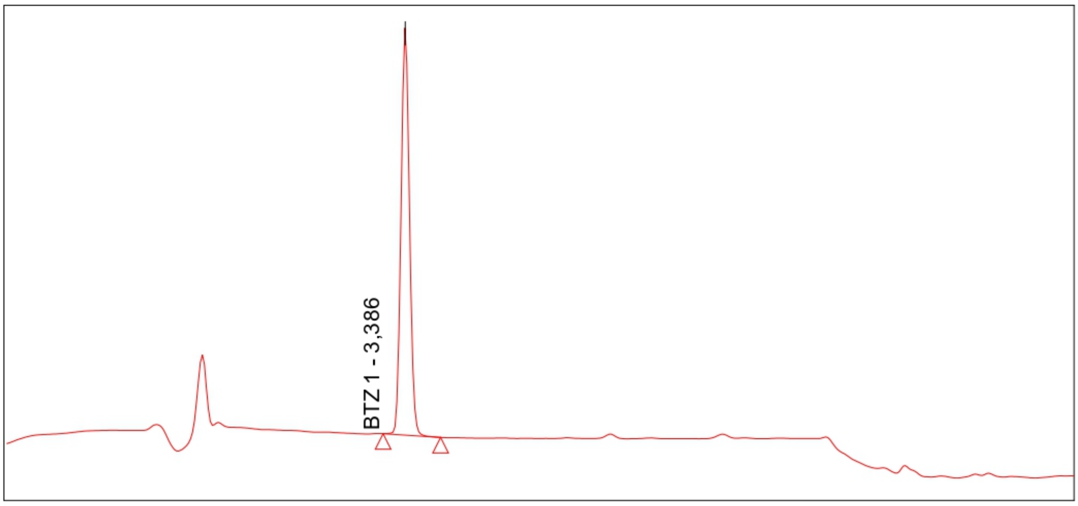

Auto-Scaled Chromatogram

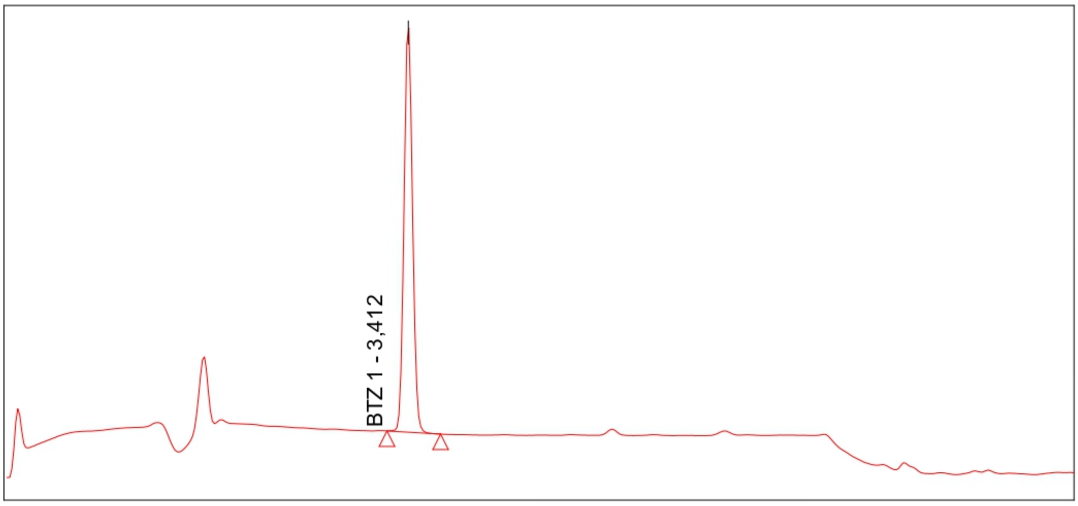

SampleName Std 80% inj 2; Date Acquired 07/02/2020 17:55:25 UYST

Peak Results

	Name	RT	Area	Height	Injection	% Area
1	BTZ 1	3,386	3800905	760611	34	100,00
2	BTZ 1	3,412	3824551	761124	35	100,00
Mean			3812727,6			
% RSD			0,4			

Spectrum Index Fraction Plot

BTZ 1 - 3,412

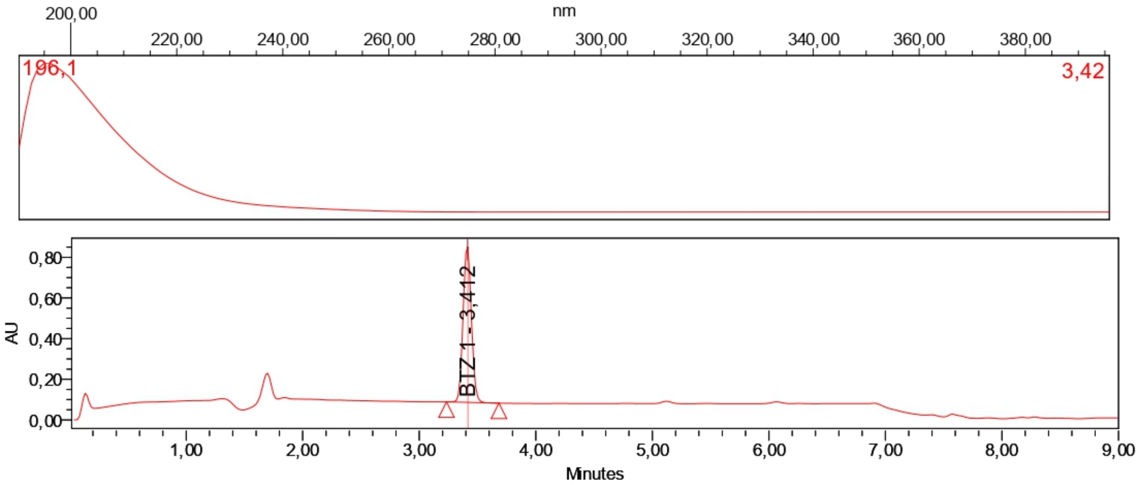

Reported by User: System
Report Method: Purezas
Report Method ID 46618
Page: 2 of 3

Project Name: Valentina y Graciela
Date Printed:
19/05/2022
17:40:53 America/Montevideo

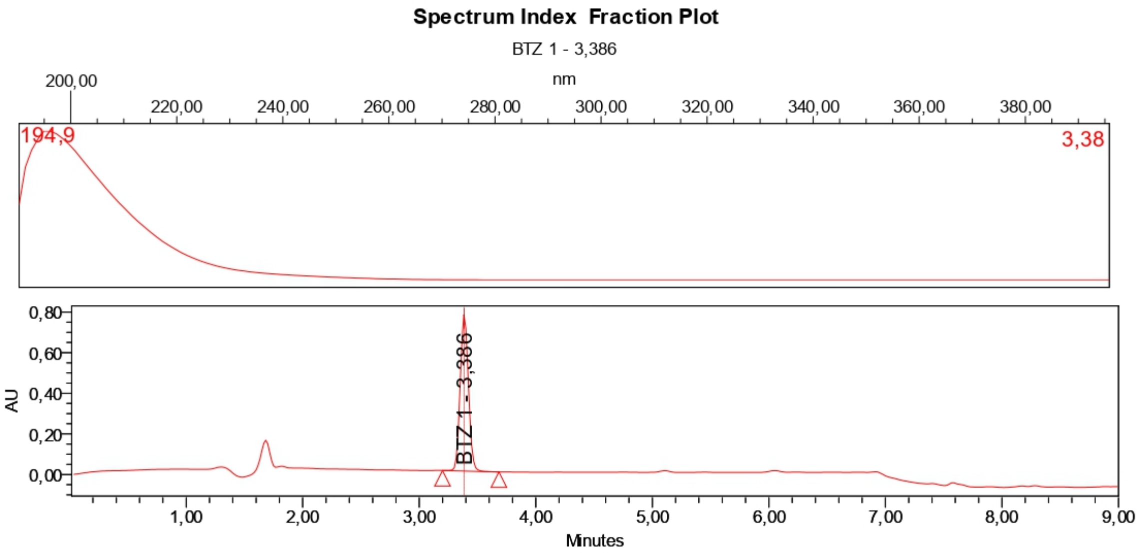

Reported by User: System
Report Method: Purezas
Report Method ID: [illegible]
Page: 3 of 3

Project Name: Valentina y Graciela
Date Printed:
19/05/2022
17:40:53 America/Montevideo

Concentration Level: 0.203 mg/mL

Empower 3 SOFTWARE

Purezas

SAMPLE INFORMATION

Sample Name:	Std 100% inj 5, Std 100% inj 3, Std 100% inj 1, Std 100% inj 4, Std 100% inj 2	Acquired By:	System
Sample Type:		Sample Set Name:	
Vial:		Acq. Method Set:	Tioester exchange
Injection #:		Processing Method:	Tiolexchange
Injection Volume:		Channel Name:	205,0nm
Run Time:	9,0 Minutes	Proc. Chnl. Descr.:	PDA 205,0 nm (PDA 190.0 to 400.0 nm at 1.2 nm)

Date Acquired: 07/02/2020 15:32:08 UYST, 07/02/2020 15:44:05 UYST, 07/02/2020 15:53:41 UYST,
Date Processed: 07/02/2020 16:03:19 UYST, 07/02/2020 16:13:29 UYST

Auto-Scaled Chromatogram

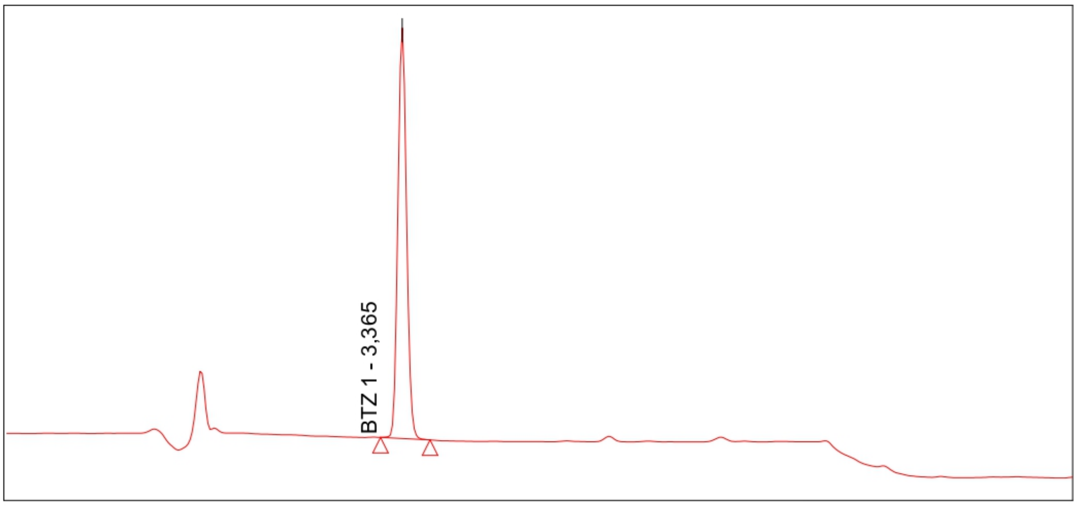

SampleName Std 100% inj 1; Date Acquired 07/02/2020 15:32:08 UYST

Reported by User: System
Report Method: Purezas
Report Method ID: [illegible]
Page: 1 of 6

Project Name: Valentina y Graciela
Date Printed:
19/05/2022
17:42:18 America/Montevideo

Auto-Scaled Chromatogram

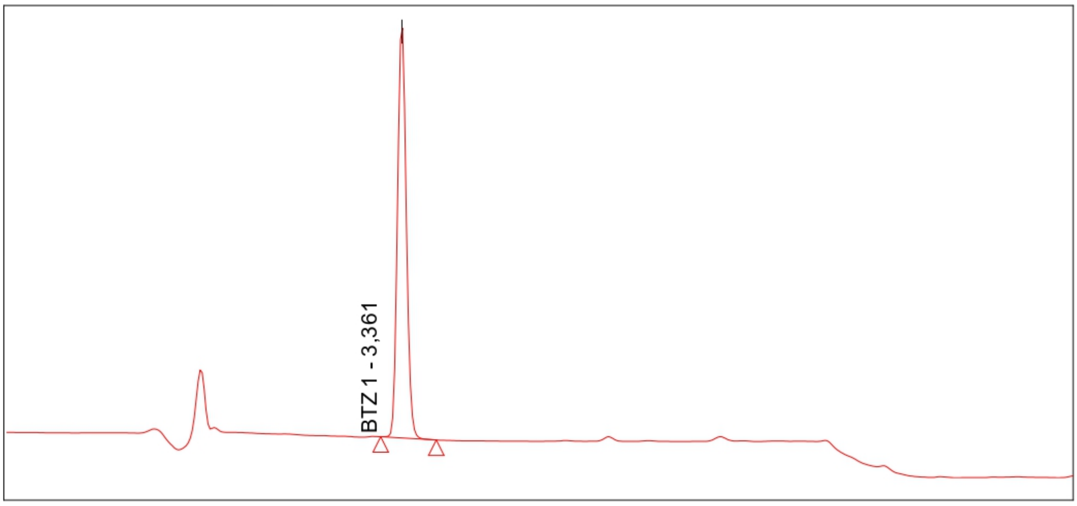

SampleName Std 100% inj 2; Date Acquired 07/02/2020 15:44:05 UYST

Auto-Scaled Chromatogram

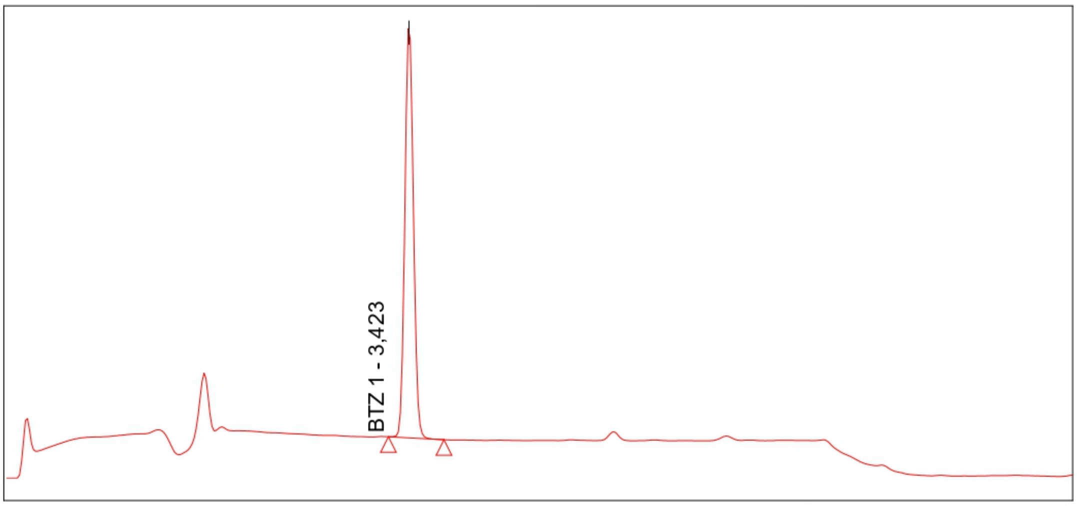

SampleName Std 100% inj 3; Date Acquired 07/02/2020 15:53:41 UYST

Auto-Scaled Chromatogram

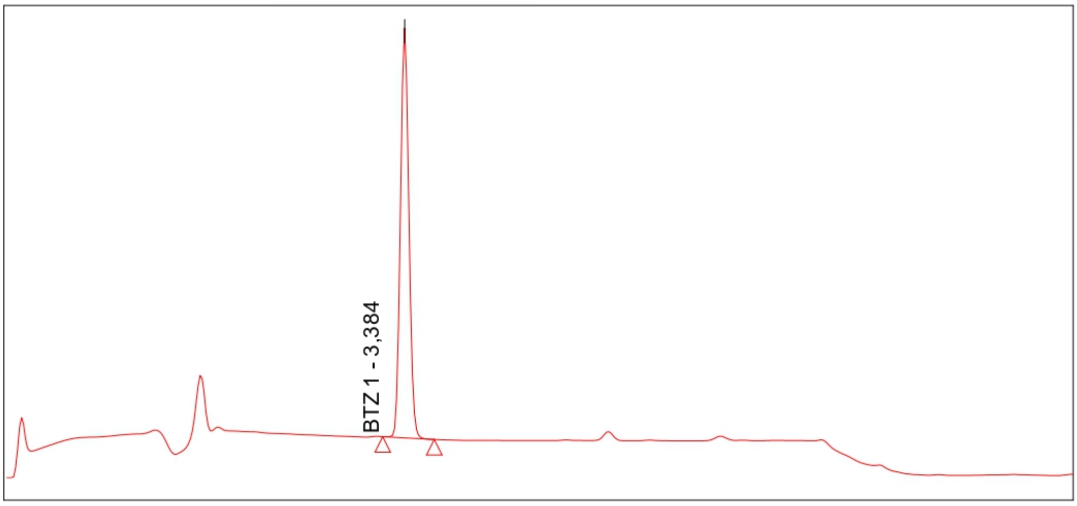

SampleName Std 100% inj 4; Date Acquired 07/02/2020 16:03:19 UYST

Auto-Scaled Chromatogram

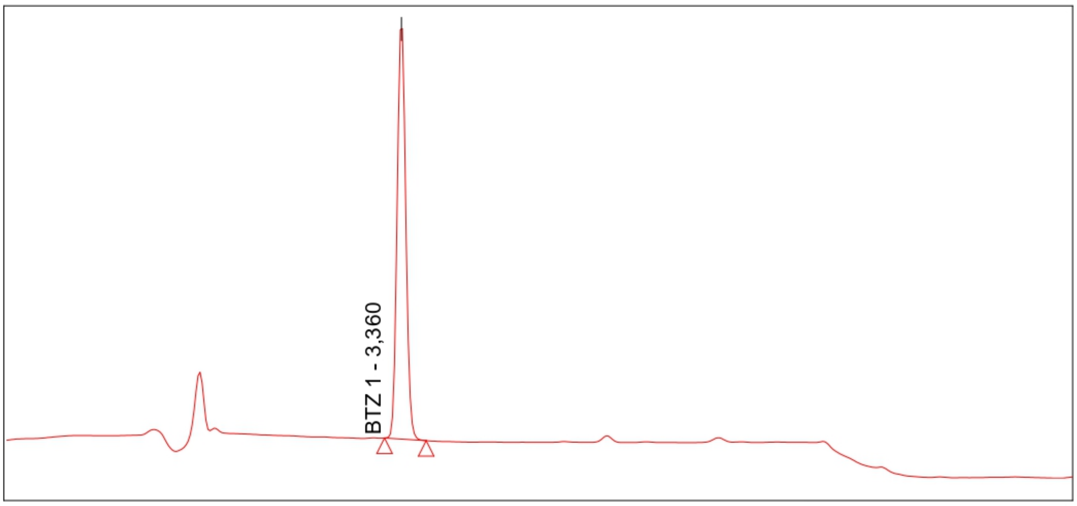

SampleName Std 100% inj 5; Date Acquired 07/02/2020 16:13:29 UYST

Peak Results

	Name	RT	Area	Height	Injection	% Area
1	BTZ 1	3,365	4603537	896885	21	100,00
2	BTZ 1	3,361	4636979	890907	22	100,00
3	BTZ 1	3,423	4607190	914049	23	100,00
4	BTZ 1	3,384	4617655	921642	24	100,00
5	BTZ 1	3,360	4710566	924548	25	100,00

Reported by User: System
Report Method: Purezas
Report Method ID: 46618
Page: 3 of 6

Project Name: Valentina y Graciela
Date Printed:
19/05/2022
17:42:18 America/Montevideo

Peak Results

	Name	RT	Area	Height	Injection	% Area
Mean			4635185,3			
% RSD			1,0			

Spectrum Index Fraction Plot

BTZ 1 - 3,360

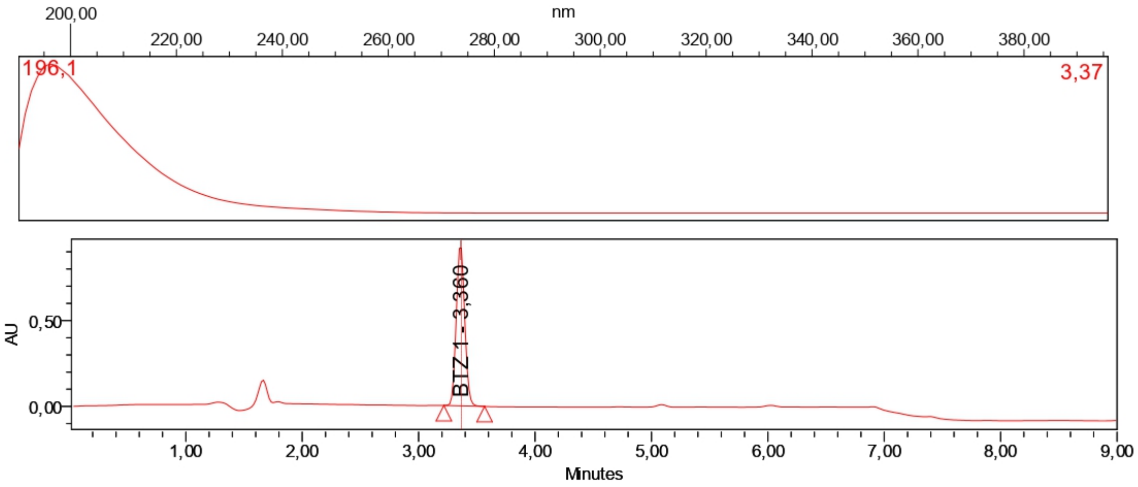

Spectrum Index Fraction Plot

BTZ 1 - 3,384

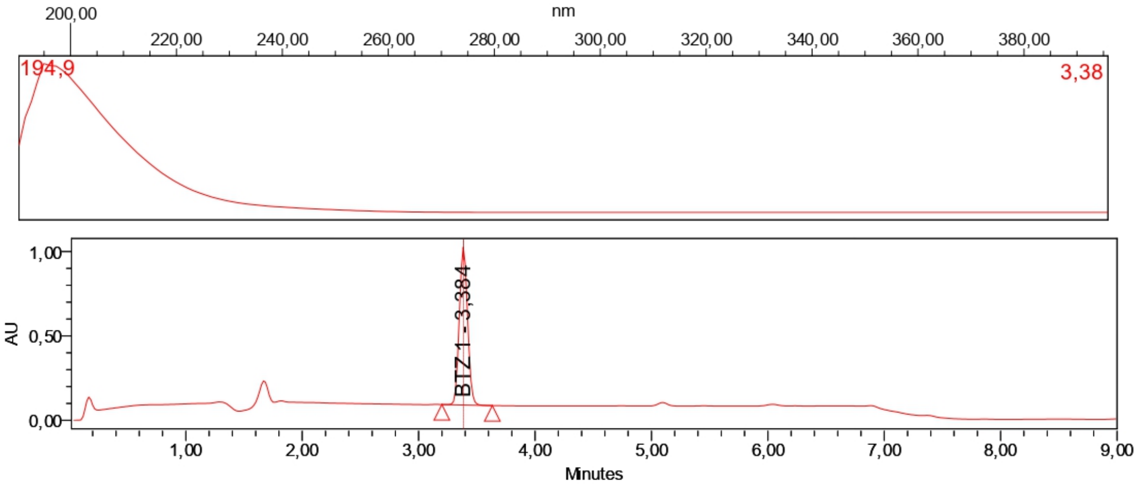

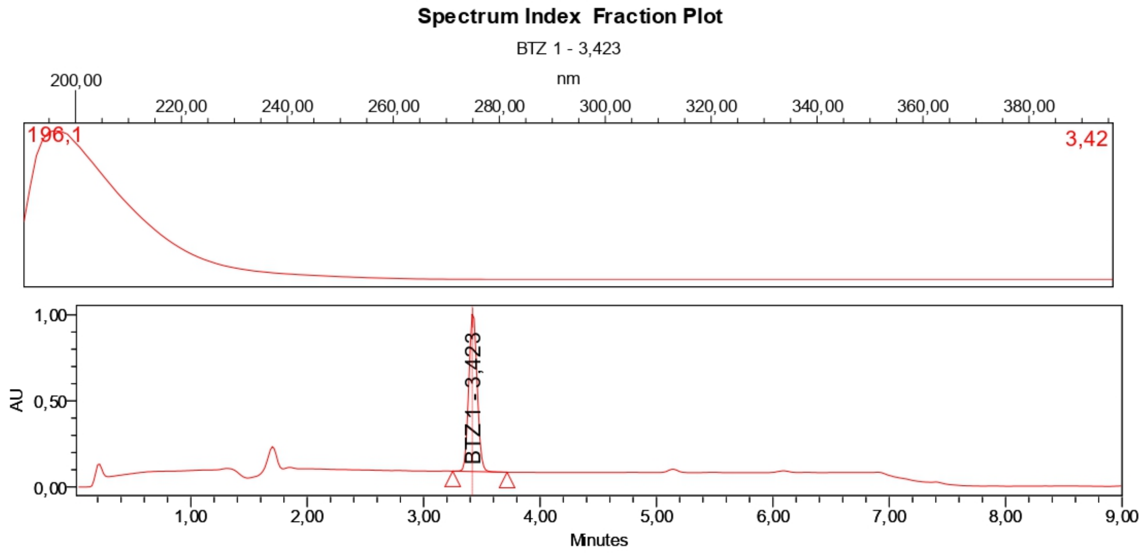
Spectrum Index Fraction Plot
BTZ 1 - 3,423
nm
196,1
3,42
AU
BTZ 1 - 3,423
Minutes

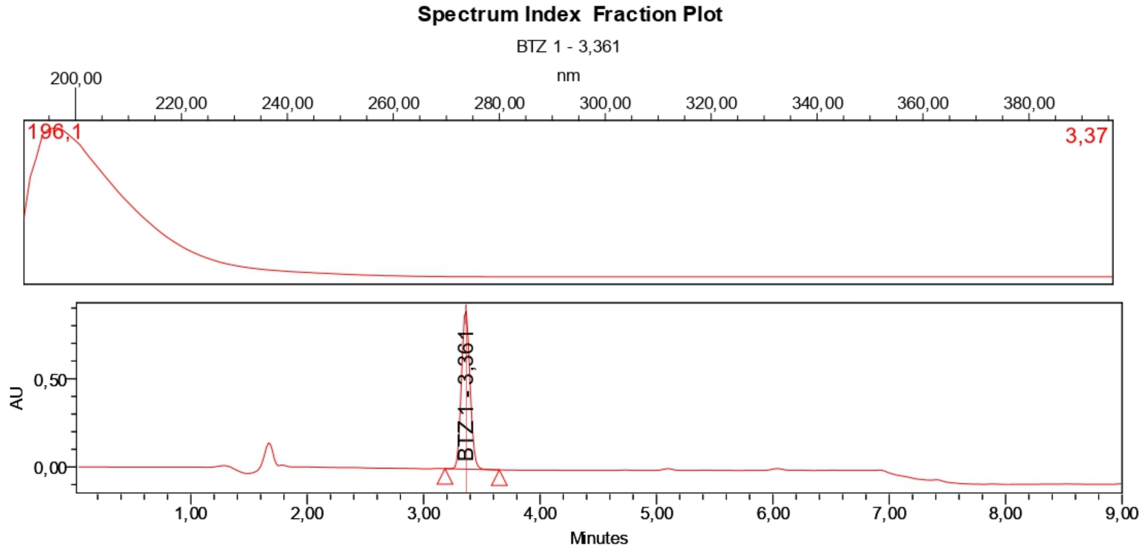
Spectrum Index Fraction Plot
BTZ 1 - 3,361
nm
196,1
3,37
AU
BTZ 1 - 3,361
Minutes

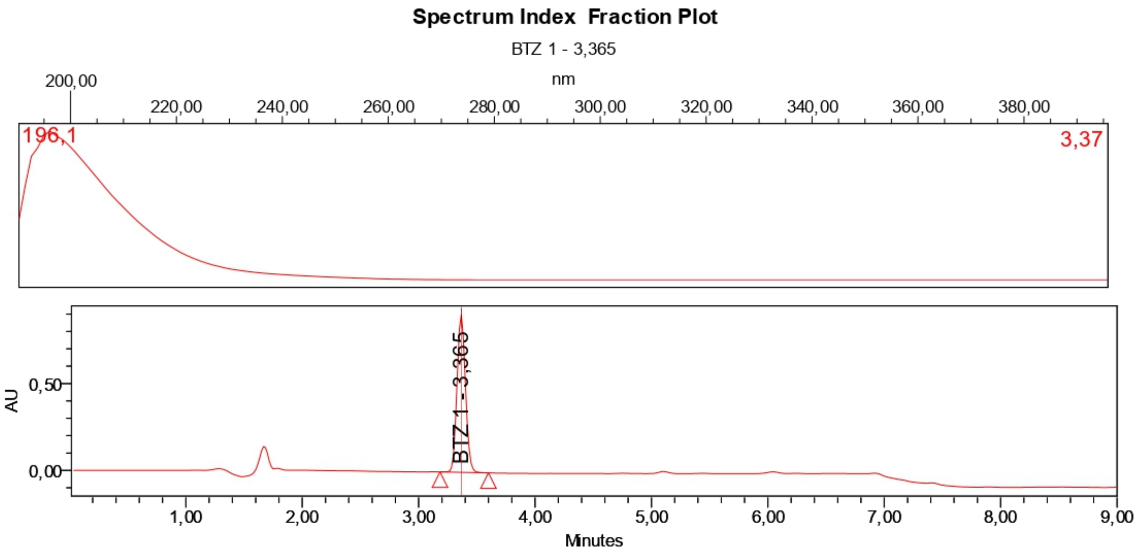
Spectrum Index Fraction Plot
BTZ 1 - 3,365
nm
200,00
220,00
240,00
260,00
280,00
300,00
320,00
340,00
360,00
380,00
196,1
3,37
AU
0,50
0,00
BTZ 1 - 3,365
1,00
2,00
3,00
4,00
5,00
6,00
7,00
8,00
9,00
Minutes

Concentration Level: 0.244 mg/mL

Empower 3 SOFTWARE

Purezas

SAMPLE INFORMATION

Sample Name:	Std 120% inj 1, Std 120% inj 2	Acquired By:	System
Sample Type:		Sample Set Name:	
Vial:		Acq. Method Set:	Tioester exchange
Injection #:		Processing Method:	Tiolexchange
Injection Volume:		Channel Name:	205,0nm
Run Time:	9,0 Minutes	Proc. Chnl. Descr.:	PDA 205,0 nm (PDA 190.0 to 400.0 nm at 1.2 nm)
Date Acquired:	07/02/2020 15:09:38 UYST, 07/02/2020 15:19:21 UYST		
Date Processed:			

Auto-Scaled Chromatogram

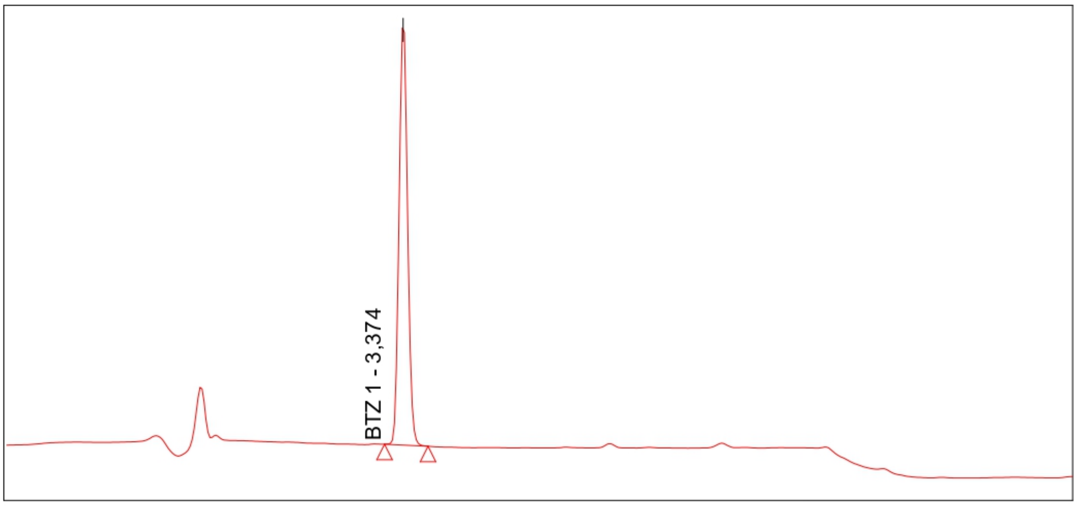

SampleName Std 120% inj 1; Date Acquired 07/02/2020 15:09:38 UYST

Auto-Scaled Chromatogram

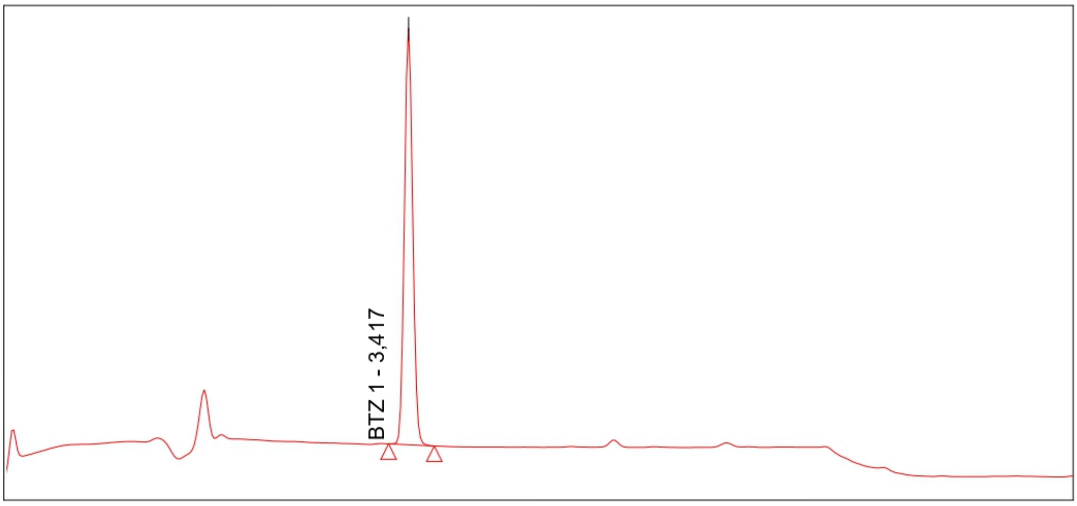

Peak Results

	Name	RT	Area	Height	Injection	% Area
1	BTZ 1	3,374	5549610	1101974	19	100,00
2	BTZ 1	3,417	5422585	1106200	20	100,00
Mean			5486097,7			
% RSD			1,6			

Spectrum Index Fraction Plot

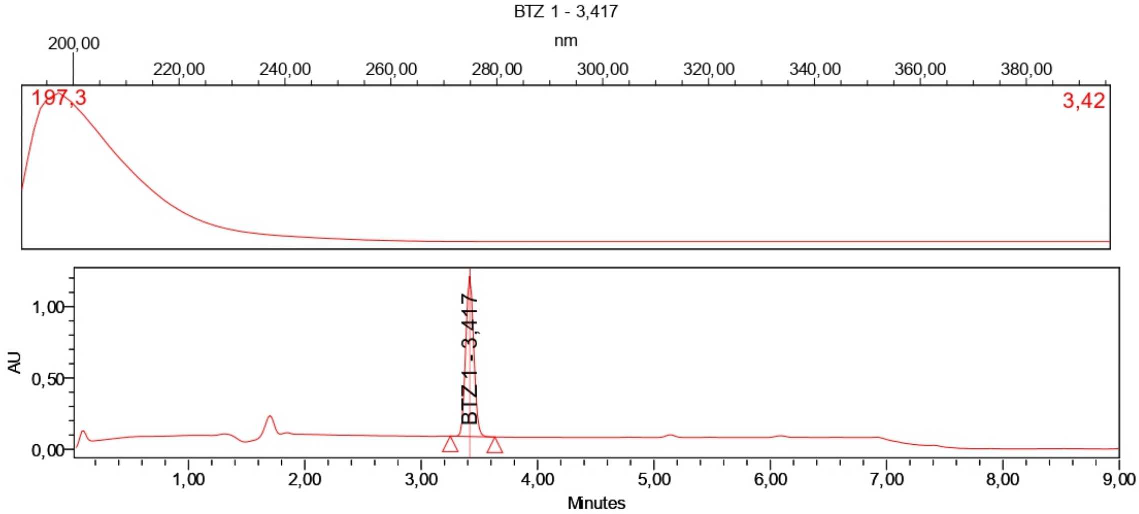

Reported by User: System
Report Method: Purezas
Report Method ID: 46188
Page: 2 of 3

Project Name: Valentina y Graciela
Date Printed:
19/05/2022
17:42:37 America/Montevideo

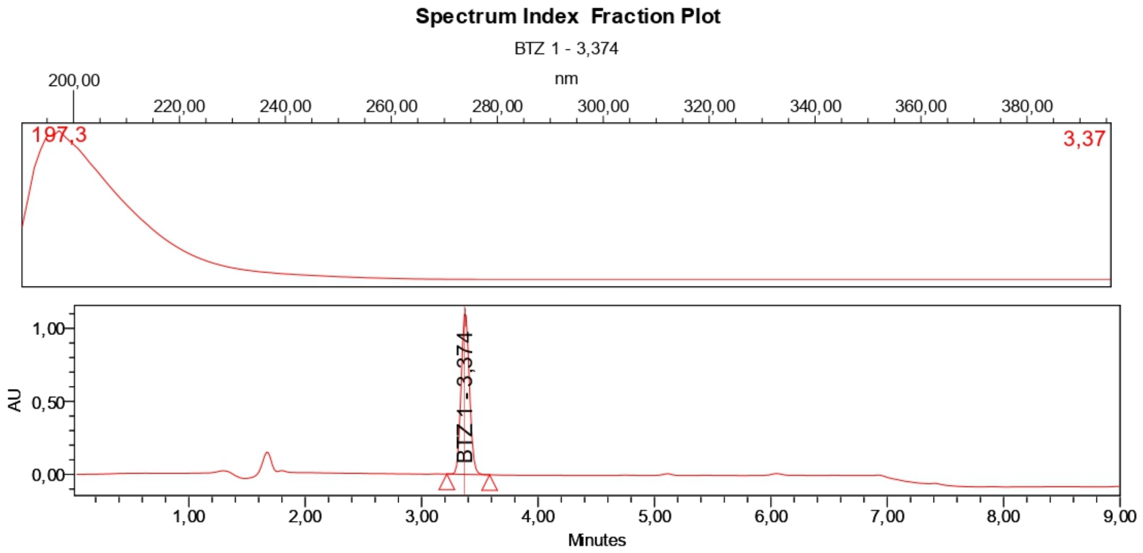

Reported by User: System
Report Method: Purezas
Report Method ID: [illegible]
Page: 3 of 3

Project Name: Valentina y Graciela
Date Printed:
19/05/2022
17:42:37 America/Montevideo

HPLC chromatogram of S-acetyl BTZ **3**

ABZ gradiente 4

SAMPLE INFORMATION

Sample Name:	Bisthiazolidine 14a	Acquired By:	System
Sample Type:	Unknown	Sample Set Name:	
Vial:	1	Acq. Method Set:	Tioester exchange
Injection #:	17	Processing Method:	Tiolexchange
Injection Volume:	20,00 ul	Channel Name:	205,0nm
Run Time:	9,0 Minutes	Proc. Chnl. Descr.:	PDA 205,0 nm (PDA 190.0 to 400.0 nm at 1.2 nm)
Date Acquired:	05/02/2020 15:28:17 UYST		
Date Processed:	19/05/2022 17:36:13 UYT		

Auto-Scaled Chromatogram

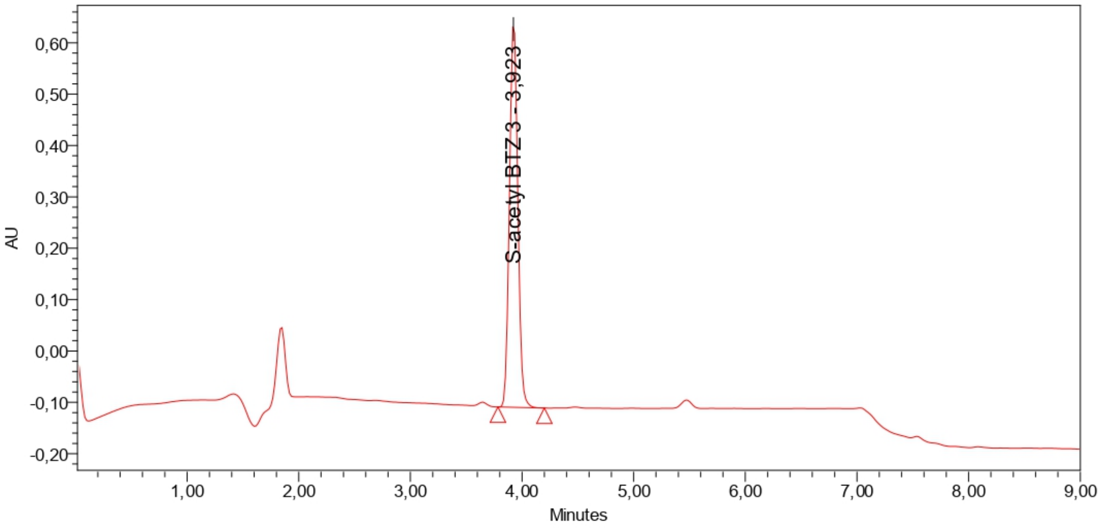

Peak Results

	Name	RT	Area	Height	Amount	Units
1	BTZ 1	3,374				
2	S-acetyl BTZ 3	3,923	3911275	743660		

Reported by User: System
Report Method: ABZ gradiente 4
Report Method ID:10331
Page: 1 of 2

Project Name: Valentina y Graciela
Date Printed:
19/05/2022
17:36:26 America/Montevideo

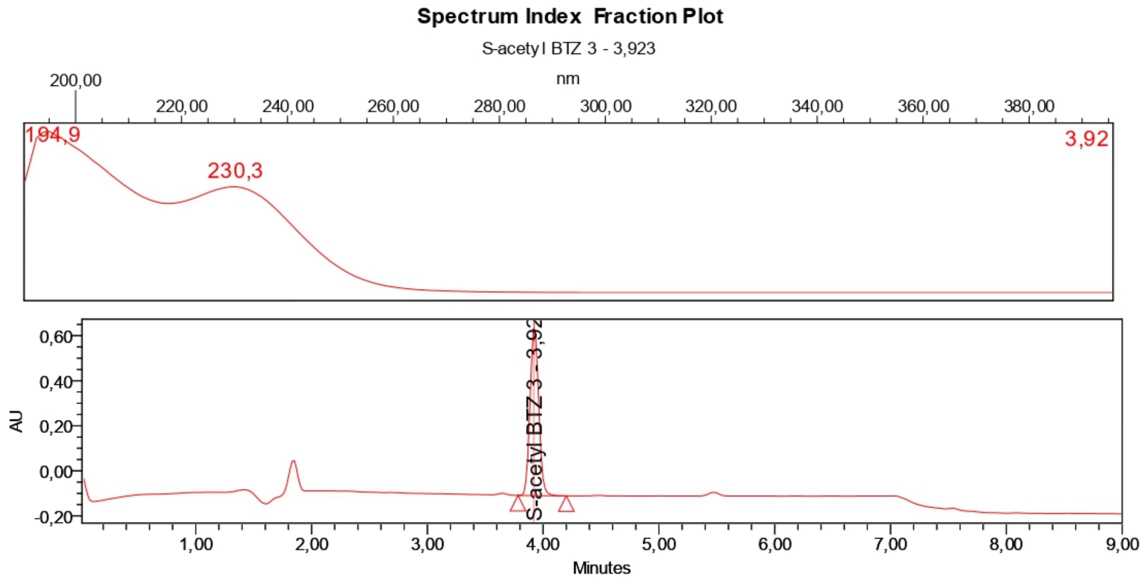

Reported by User: System
Report Method: ABZ gradiente 4
Report Method ID:10331
Page: 2 of 2

Project Name: Valentina y Graciela
Date Printed:
19/05/2022
17:36:26 America/Montevideo
